# Immunogenicity and Safety of a Combined Intramuscular/Intranasal Recombinant Spike Protein COVID-19 Vaccine (RCP) in Healthy Adults Aged 18 to 55 Years Old: A Randomized, Double-Blind, Placebo-Controlled, Phase I Trial

**DOI:** 10.3390/vaccines11020455

**Published:** 2023-02-16

**Authors:** Masoud Solaymani Dodaran, Seyed Reza Banihashemi, Ali Es-haghi, Mohammad Hossein Fallah Mehrabadi, Mojtaba Nofeli, Ali Rezaei Mokarram, Ladan Mokhberalsafa, Fariba Sadeghi, Alireza Ranjbar, Akram Ansarifar, Arash Mohazzab, Seyed Amin Setarehdan, Fahimeh Bagheri Amiri, Vahideh Mohseni, Monireh Hajimoradi, Neda Ghahremanzadeh, Seyed Hossein Razzaz, Safdar Masoomi, Maryam Taghdiri, Mohsen Bagheri, Mohsen Lofti, Akbar Khorasani, Masoud Ghader, Shiva Safari, Masumeh Shahsavn, Saeed Kalantari

**Affiliations:** 1 Clinical Trial Center, Iran University of Medical Science, Tehran 1449614535, Iran; 2 Division of Epidemiology and Public Health, University of Nottingham, Nottingham NG7 2UH, UK; 3 Minimally Invasive Surgery Research Center, Hazrat-e-Rasool Hospital, Iran University of Medical Science, Tehran 1445613113, Iran; 4 Department of Immunology, Razi Vaccine and Serum Research Institute, Agricultural Research, Education and Extension Organization (AREEO), Karaj P.O. Box 31975/148, Iran; 5 Department of Physico Chemistry, Razi Vaccine and Serum Research Institute, Agricultural Research, Education and Extension Organization (AREEO), Karaj P.O. Box 31975/148, Iran; 6 Department of Epidemiology, Razi Vaccine and Serum Research Institute, Agricultural Research, Education and Extension Organization (AREEO), Karaj P.O. Box 31975/148, Iran; 7 Department of Research and Development, Razi Vaccine and Serum Research Institute, Agricultural Research, Education and Extension Organization (AREEO), Karaj P.O. Box 31975/148, Iran; 8 Department of QA, Razi Vaccine and Serum Research Institute, Agricultural Research, Education and Extension Organization (AREEO), Karaj P.O. Box 31975/148, Iran; 9 Department of Health, Razi Vaccine and Serum Research Institute, Agricultural Research, Education and Extension Organization, Karaj P.O. Box 31975/148, Iran; 10 Institute of Interventional Allergology and Immunology, Bonn/Cologne, 53115 Bonn, Germany; 11 School of Public Health, Iran University of Medical Science, Tehran 1449614535, Iran; 12 Reproductive Biotechnology Research Center, Avicenna Research Institute Tehran, ACECR, Tehran 1983969412, Iran; 13 Department of Epidemiology and Biostatistics, Research Centre for Emerging and Reemerging Infectious Diseases, Pasteur Institute of Iran, Tehran 1316943551, Iran; 14 Department of Biostatistics, Faculty of Medical Sciences, Tarbiat Modares University, Tehran P.O. Box 14115/111, Iran; 15 Departments of Infectious Diseases and Tropical Medicine, Iran University of Medical Sciences, Tehran 1449614535, Iran

**Keywords:** SARS-CoV-2, recombinant vaccine, immune response, immunogenicity, intranasal vaccine

## Abstract

**Objectives**: This study aimed to determine the safety and immunogenicity of a combined intramuscular/intranasal recombinant spike protein COVID-19 vaccine (RCP). **Methods**: We conducted a randomized, double-blind, placebo-controlled, phase I trial. Three vaccine strengths were compared with an adjuvant-only preparation. It included two intramuscular and a third intranasal dose. Eligible participants were followed for adverse reactions. Specific IgG, secretory IgA, neutralizing antibodies, and cell-mediated immunity were assessed. **Results**: A total of 153 participants were enrolled (13 sentinels, 120 randomized, 20 non-randomized open-labeled for IgA assessment). No related serious adverse event was observed. The geometric mean ratios (GMRs) and 95% CI for serum neutralizing antibodies compared with placebo two weeks after the second injection were 5.82 (1.46–23.13), 11.12 (2.74–45.09), and 20.70 (5.05–84.76) in 5, 10, and 20 µg vaccine groups, respectively. The GMR for anti-RBD IgA in mucosal fluid two weeks after the intranasal dose was 23.27 (21.27–25.45) in the 10 µg vaccine group. The humoral responses were sustained for up to five months. All vaccine strengths indicated a strong T-helper 1 response. **Conclusion**: RCP is safe and creates strong and durable humoral and cellular immunity and good mucosal immune response in its 10 µg /200 µL vaccine strengths. **Trial registration**: IRCT20201214049709N1.

## 1. Introduction 

The protein subunit platform is one of the oldest and most widely used vaccine platforms [1]. Vaccines using this platform have fewer potential safety concerns and lower production costs [1,2] They need an adjuvant to induce the required immune response and using an appropriate one, they are a potent inducer of cellular immunity [3,4]. A third COVID-19 vaccine in the clinical development phase has used this platform (COVID-19 vaccine tracker and landscape: https://www.who.int/publications/m/item/draft-landscape-of-COVID-19-candidate-vaccines accessed on 1 June 2022). They all target COVID-19 spike protein [2,5]. The glycosylated spike (s) is a trimetric protein, the main viral antigenic target, essential for viral binding, fusion, and uptake into mammalian cells [3]. The S protein consists of two subunits, namely the S1 domain for viral attachment to host cells and the S2 domain for virus–cell membrane fusion [6].

Deep muscular injections are the usual way of delivering COVID-19 vaccines. However, it is ineffective in inducing mucosal immunity [7]. Mucosal defense is an essential part of our body’s defense against COVID-19 [8]. Typically, the virus first comes into contact with the nasopharyngeal mucosa. Mucosal primary target cells need secretory IgA to effectively prevent viral replication within the nasopharynx, which generally requires a mucosal route of vaccination [9]. Intranasal vaccination has been reported as an effective strategy for reducing virus concentration and virus shedding in various animal models (chimpanzees [10], mice [11,12], and rhesus macaques [13]) and humans alike [8]. Therefore, many research teams are currently working on developing a COVID-19 vaccine with an effective intranasal route of delivery [14].

RCP is a combined intramuscular/intranasal recombinant S protein COVID-19 vaccine, functioning through a cocktail of spike antigens (S1, S2, and S-Trimer using the sequence ofSARS-CoV-2 variant in Wuhan) formulated using the oil-in-water adjuvant system RAS-01 (Razi Adjuvant System-01) [15,16]. Its particular design structure allows it to be used on mucosal surfaces. It includes two injections on days 0 and 21 and one intranasal dose on day 51 using an intranasal mucosal atomization device. The Razi Adjuvant System-01 (RAS-01) (Razi Vaccine and Serum Research Institute, Karaj, Iran) is an Iranian FDO (Food and Drug Organization) approved oil-in-water emulsion composed of sesame, olive, and soybean oils and the non-ionic surfactant Tween 80. A detailed description of its preparation has been published before [15].

The rationale for including S1 and S2 subdomains alongside the trimeric S protein in the vaccine formulation is based on the following two points: First, conventional use of the amount of S antigen may induce harmful immune responses that cause liver damage. The use of antigen fragments could reduce side effects and increase immunogenicity [17]. Second, as S1 and S2 are the main subdomains of the S protein, using S1 and S2 instead of S provides the opportunity to use different concentrations of these two in the formulation of the vaccine. Given that S2 accommodates more preserved parts of the spike genome, its inclusion in the formulation of the vaccine results in higher resistance/better performance against new strains of the virus [18,19,20].

Preclinical studies showed promising safety, humoral, and cellular immunogenicity results in Syrian hamsters, BALB/c mice, Pirbright guinea pigs, and NZW rabbits. Specific anti-RBD IgA antibodies peaked following the administration of the intranasal dose in guinea pigs and persisted for several months in the saliva and serum [15]. In this study, we examined the safety and immunogenicity, including the mucosal secretory IgA response, of the combined intramuscular/intranasal recombinant spike protein COVID-19 vaccine (RCP) in healthy adults aged 18 to 55 years old.

## 2. Materials and Methods

### 2.1. Study Design

We began the trial with a small group of participants (first step) as sentinels. We then completed a single-center, randomized, double-blind controlled, dose-finding trial with four arms (three strengths of the vaccine at 5, 10, and 20 μg/200 μL and an adjuvant-only group) to assess the safety and immunogenicity of the RCP recombinant spike protein vaccine (second step). Once the first interim analysis results were known and a decision on the selected vaccine strength for the phase II trial was made, we recruited an additional group of open-labeled and non-randomized subjects to evaluate mucosal immunity. They received the selected vaccine strength for their first two intramuscular doses and either 10 µg intranasal vaccine or placebo for their third dose (third step). The clinical Trial Center of the Iran University of Medical Sciences (CTC-IUMS), as the academic CRO (contract research organization), conducted this study.

### 2.2. Participants

Healthy Iranian men and non-pregnant women aged 18 to 55 and with a BMI of 17 to 35, with no current or previous history of COVID-19 infection (negative PCR and anti-nucleocapsid (N) antibodies) and no history of COVID-19 vaccination, were eligible to enter this study. We obtained written informed consent from all participants before the screening. The main inclusion and exclusion criteria were as follows: being healthy based on the clinical, psychological, and laboratory criteria; signing a written informed consent form; not having any ongoing, symptomatic, acute, or chronic illness requiring medical or surgical care on the day of vaccination; negative ELISA test for HIV, HCV, and HBV; negative pregnancy test based on βHCG on the day of screening and the day of vaccination; consenting to continue on one effective method of contraception up to three months after the last vaccine dose; not working in an occupation with a high risk of exposure to COVID-19; no history of long-term use of immunosuppressive medication from four months before up to the screening day; no history of allergic diseases, such as angioedema or anaphylactic reactions, or any allergy to the drug or vaccine.

### 2.3. Randomization and Masking

We used block randomization with variable block sizes to allocate the study participants into the three groups receiving three different strengths of the vaccine and a placebo (adjuvant-only) group, all in single-dose preparations. Blood specimens collected to assess immunogenicity were coded to blind the immunology lab operators during this research (for a detailed description, see the study protocol).

### 2.4. Procedures

The four study groups were used to compare the strengths of 5, 10, and 20 μg/200 μL of the recombinant spike protein COVID-19 vaccine in an oil-in-water adjuvant and the adjuvant-only preparation. The eligible participants were randomly allocated to receive 0.2 mL of vaccine/placebo via intramuscular injection into the deltoid muscle on days 0 and 21, followed by a 0.2 mL of the specially prepared intranasal spray of 10 μg vaccine/placebo on day 51.

Volunteers who passed the initial psychological assessment step were invited to the study site for a face-to-face interview, signing the informed consent form, physical examination, and giving blood and nasopharyngeal samples for an additional screening and PCR test for COVID-19 infection. The eligible individuals were invited to receive the vaccine or placebo.

A small group of sentinel subjects received either a placebo or one of three strengths of the vaccine in an open-labeled and non-randomized manner. The data and safety monitoring board (DSMB) reviewed the safety information of this small group before granting permission to start the main recruitment phase of the study.

We put the participants under close observation for 3 h and monitored their vital signs after each vaccine dose. Local and systemic reactions were assessed through daily calls for six days, and the relevant forms were filled. A 24 h call center with a resident physician was established to provide medical consultation support for the participants. Each participant was invited based on a predefined schedule for physical examination and sampling (Table 1). Any outpatient or inpatient visit to the health professionals was recorded and followed to detect the medically attended adverse events (MAAEs) (Table 1).

IgG antibody responses against specific S, S1, S2, RBD (Receptor Binding Domain), and NTD (N-Terminal Domain) antigens and IgA antibodies for the RBD antigen were measured using house ELISA kits and specific COVID-19 antigens (Native Antigen, Oxford, UK). We tested six serum dilutions (0.1, 0.01, 0.001, 0.0001, 0.00001, and 0.000001) for each serum specimen and calculated the area under the curve (AUC). Resistance against changing variants was explored using S1 and S-Trimer antigenic components (Native Antigen, Oxford, UK) of the Wuhan, Delta, and Omicron SARS-CoV-2 variants. Serum neutralizing antibody responses were assessed using a conventional virus neutralization test (cVNT) conducted in a biosafety level 3 laboratory facility. The original live SARS-CoV-2 virus, isolated from Iranian COVID-19 patients, was used for cVNT. We regarded a four-fold increase in antibody titer as seroconversion [21]. We did not perform conventional VNT on nasopharyngeal mucosal fluid samples because saliva contains different microorganisms that contaminate the cell culture. It also contains a variety of enzymes that can damage the cells in the culture. Nasopharyngeal mucosal fluid-neutralizing antibody activity was evaluated using a human ACE2 protein (hACE-2) binding assay. We performed a range of dilutions in the test design, and when we reached the 1:100 dilution, we were able to omit the background signals. In this dilution, we were able to detect highly sensitive and specific responses from the targeted serum.

Peripheral blood mononuclear cells were used to assess the cellular immunity response. We tested them for specific interleukin-secreting T cells before and after the stimulation using a specific COVID-19 S1 antigen and inactive virus. Lymphocyte proliferation was evaluated using the CFSE method. The expression of Gamma interferon (IFNγ), tumor necrosis factor (TNFα), and interleukin IL-2, 4, 6, and 17 were detected using an Enzyme-Linked Immunosorbent Assay (ELISA) (R&D, Boston, MA, USA). The number of CD3, CD4, CD8, CD3/CD8, and CD3/CD4 cells was counted using flow cytometers (Monoclonal antibody, BD, Richmond, CA, USA) (Supplementary File S1).

### 2.5. Outcomes

The primary safety outcomes were the frequency and percentage of the participants with anaphylactic reactions or abnormal vital signs within three hours of receiving each vaccine dose, solicited local (pain, tenderness, redness/erythema, itching, and swelling/induration), and systemic (fever, nausea or vomiting, diarrhea, headache, fatigue, and myalgia) reactions up to a week after receiving the vaccine and abnormal laboratory findings one week following each vaccine dose. We used modified FDA toxicity scoring (Tables S1–S3) to classify severity (Guidance for industry: toxicity grading scale for healthy adult and adolescent volunteers enrolled in preventive vaccine clinical trials. In: Food and Drug Administration, US Department of Health and Human Services. 2007).

The secondary safety outcomes were the number and percentage of serious adverse events (SAEs), suspected unexpected serious adverse reactions (SUSARs), and medically attended adverse events (MAAEs) collected during each visit and the monthly follow-up calls for up to six months. The secondary immunogenicity outcomes were the specific IgG antibody responses to S, S1, S2, RBD, and NTD antigens in the vaccine groups two weeks after the second dose (day 35), IgA antibody response to RBD antigen in nasopharyngeal mucosal fluid (on days 65, 120, and 150), the proportion of participants with a four-fold increase in neutralizing antibody response to live SARS-CoV-2 virus antigen on day 35, and specific T cell (Th1 and Th2 specific cellular immunity) response to the SARS-CoV-2 S1 antigen and inactivated virus.

### 2.6. Statistical Analysis

The sample size was not based on a test of statistical hypotheses. After each vaccine dose, we used descriptive summary statistics to evaluate the primary safety objectives. Secondary immunization outcomes were summarized as the geometric mean (GM) and geometric mean ratio (GMR), in which a 95% confidence interval was observed at different points in time. The geometric mean and 95% CI were calculated based on t and log-transformed distributions. The GMR index (95% CI) was calculated based on Dunnett’s test, which was used to adjust multiple comparisons with one control. Seroconversion, measured using ELISA, was defined as a 4-fold increase in the antibody area under the curve (AUC) compared with the baseline. The safety and immunogenicity data on the sentinels were analyzed separately and combined with the rest of the data from the four main study groups. We included all the participants with available data in the safety and immunogenicity analyses.

A nine-member data and safety monitoring board supervised the conduct, data collection, and safety outcomes. Assigned representatives from the National Ethics Committee (NEC), the Ministry of Health’s Food and Drug Organization (FDO), and the Communicable Disease Control (CDC) department attended all the sessions. The four independent DSMB members included an internist, an infectious disease specialist, a pharmacotherapist, and a specialist in immunology and allergy. Two other non-voting members were representing the sponsor. Permission to enter the main recruitment phase was granted by the committee upon reviewing the safety data from the sentinel group. This study is registered in www.irct.ir (IRCT20201214049709N1).

## 3. Results

**Figure 1:**
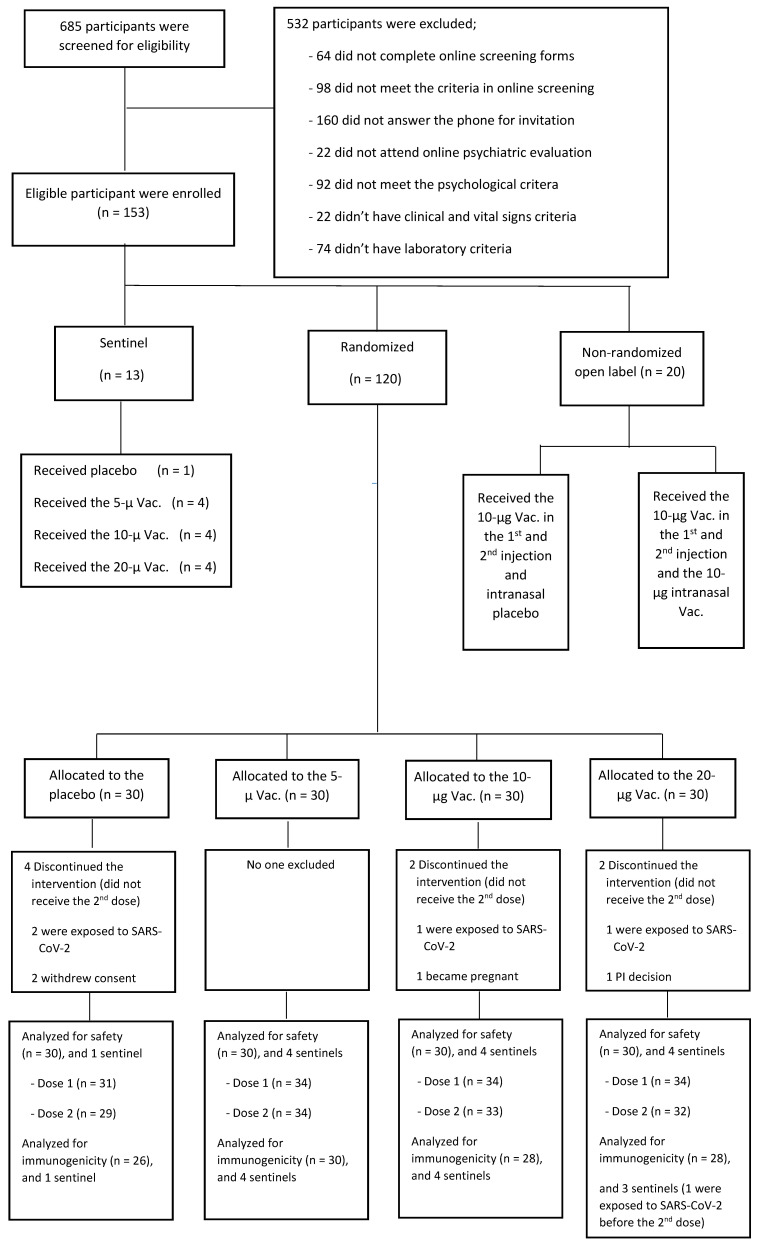
Participant flow chart.

Between 28 February and 10 April 2021, 685 individuals underwent screening, and 153 eligible participants entered the study in three steps. In the first step, one person received a placebo (adjuvant only), and three groups of four received the vaccine with a strength of 5, 10, and 20 µg/200 µL in 48 h intervals, respectively (a sentinel group of thirteen). In the second step, 120 individuals were randomly assigned into 4 groups of 30, receiving a placebo and the 3 strengths of the vaccine. In the third step, two groups of ten participants received either an intranasal vaccine or a placebo. Out of the 133 subjects enrolled in the first two steps, 124 were analyzed in four study groups, including 27, 34, 32, and 31 in the placebo and the 5, 10, and 20 µg/200 µL vaccine groups, respectively (see Figure 1). The baseline characteristics of all the groups were similar (Table 2 and Table S4).

**Figure 2:**
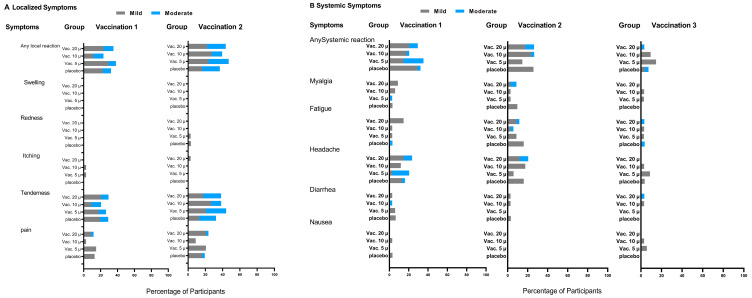
Solicited local and systemic adverse reactions. The percentage of the participants with adverse reactions in each intervention group according to the FDA toxicity grades (mild, moderate, severe, and life-threatening) during the seven-day period after each dose. There were no grade 3 (severe) or grade 4 (life-threatening) events.

We did not observe any immediate allergic reactions. The participants reported only mild to moderate (grade II or lower) solicited local and systemic reactions during the first week after each vaccine dose, which was similar in all four study groups (Figure 2). The most common local adverse reaction was tenderness at the injection site. At least one local grade II reaction was seen in seven (22.6%), nine (26.5%), eight (23.5%), and eight (23.5%) participants following the two injections in the placebo, 5, 10, and 20 µg vaccine groups, respectively. Systemic grade II adverse reactions were seen in one (3.2%), seven (20.6%), two (5.9%), and six (17.5%) subjects in each of the study groups, respectively. The most prevalent systemic adverse reaction was headache (see Table S6). Adverse reactions following the intranasal dose were rare (Table S7 and Figure 2).

We detected 87 unsolicited adverse events in 63 individuals over a 6-month follow-up. No vaccine/placebo-related serious adverse event was seen. Medical attention was required on six occasions: two cases of exacerbation of migraine, one case of dizziness diagnosed as a vestibular disorder, one case of cellulitis, which was treated by antibiotics, one case of generalized urticaria following intranasal dose, and one case of radial nerve injury caused by the injection (see Supplementary Material, Table S8). All the cases received the necessary outpatient treatments and were followed until complete recovery. A chemical pregnancy case was a report of menstrual retardation after receiving the first dose in the 10 µg vaccine group in a 35-year-old woman with a history of fertility problems. She did not receive the second dose of the vaccine and reported a positive pregnancy test (β-HCG = 107) and vaginal bleeding around the same time four weeks after receiving the first dose. This pregnancy did not lead to para-clinical detection of the fetal pole in ultrasound and was unlikely to be related to the vaccine. Abnormal laboratory findings were generally mild (grade II or lower), equally distributed among study groups (see Supplementary Material, Table S11), and ultimately resolved within a few weeks. We observed only one case of grade III increase in liver enzymes (ALT = 187 IU/dL) that returned to the normal range within 30 days.

**Figure 3:**
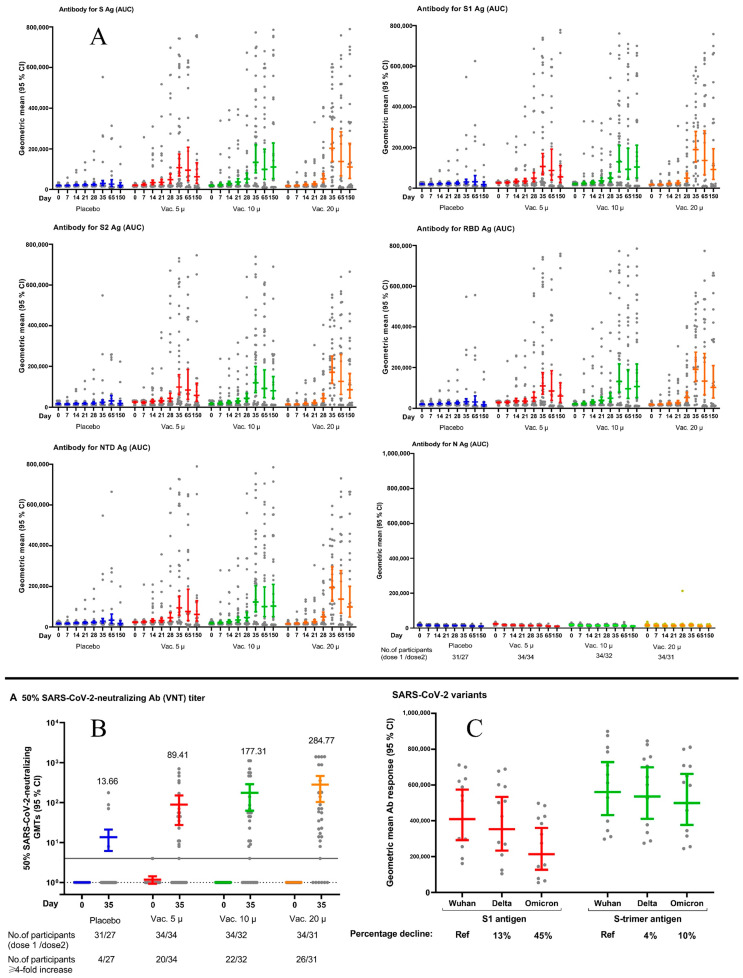
(**A**) Scatter diagram of specific IgG antibody responses (AUC) against S, S1, S2, RBD, NTD, and N (Nucleocapsoid) antigen (SARS-CoV-2 variant in Wuhan) in the intervention groups over the predefined study time schedule. Geometric means and 95% confidence intervals have been shown on the diagram. (**B**) Scatter diagram of serum neutralizing antibody titers (cVNT with the SARS-CoV-2 variant in Wuhan) in the intervention groups over the predefined study time schedule. Geometric mean titers giving 50% serum neutralization and 95% confidence intervals are shown in the diagram. The horizontal solid line is the cut-off titer of 1:4. The horizontal dotted line is the baseline titer. (**C**) ELISA-specific IgG antibody responses against s1 and s-trimer antigenic components by changing SARS-CoV-2 variants over time (SARS-CoV-2 variant in Wuhan, Delta, and Omicron) in the 10 µg vaccine group.

All vaccine groups induced significant humoral antibody responses (Figure 3A and Supplement Tables S12–S16). The geometric mean ratios (GMRs) and their 95% confidence interval, compared with placebo on day 35 for S antigen, were 3.44 (1.66–7.14), 4.19 (2.00–8.79), and 6.12 (2.90–12.89) in the 5, 10, and 20 µg vaccine groups, respectively (see Table 3 and Table S12). The corresponding figures for RBD (see Table 3 and Table S15) were 3.40 (1.63–7.07), 4.07 (1.94–8.55), and 5.88 (2.78–12.44). They remained relatively unchanged over the rest of the study period. The GMRs for the S antigen on day 150 in the three vaccine groups were 3.20 (1.70–9.03), 5.60 (1.68–27.39), and 5.61 (1.70–25.28), respectively. The other specific antibodies (S1, S2, RBD, and NTD) except N also showed similar patterns of increase (see Figure 3 and Supplement Tables S13, S14 and S16). We observed a small decline in ELISA specific antibody responses against S-Trimer by changing SARS-CoV-2 variants over time (see Figure 3C). The decline was more marked against the S1 antigenic component.

**Figure 4:**
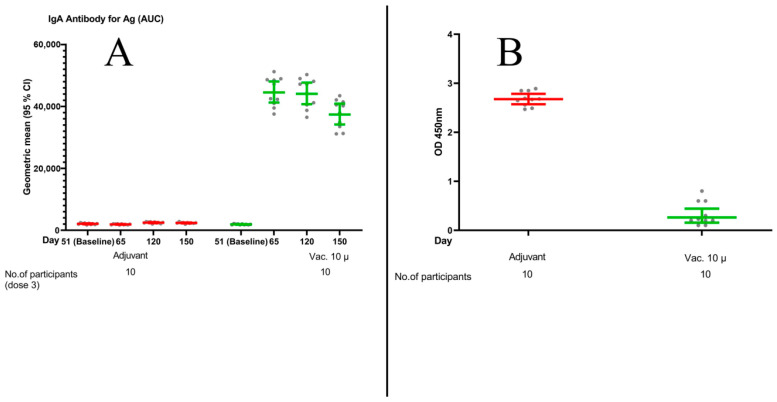
(**A**) Scatter diagram of specific secretory IgA response in mucosal fluid (AUC) against RBD antigen in the nonrandomized open-labeled groups (those who received an intranasal dose of the 10 µg vaccine versus the placebo) over the predefined study time schedule. Geometric means and 95% confidence intervals are shown in the diagram. (**B**) Scatter diagram of human ACE2 protein (hACE-2) adhesion inhibition assay results two weeks after receiving an intranasal dose in the 10 µg vaccine group vs. the placebo group. Percent of binding inhibition was calculated [1 − (∑OD450vaccine/∑OD450adjuvant)] × 100 as 87.6%.

The GMR for anti-RBD secretory IgA in mucosal fluid, at two weeks after the intranasal dose, was 23.27 (21.27–25.45) in the participants receiving the 10 µg vaccine compared to placebo, indicating a robust mucosal immunity response. The corresponding figures for 70 and 100 days after the intranasal dose were 17.85 (16.13–19.77) and 15.76 (14.23–17.45) (see Figure 4A). We observed significant inhibition of ACE2 adhesion in the mucosal fluid of the participants receiving the intranasal 10 µg vaccine compared with the lack of inhibition in the recipients of the intranasal placebo (*p* < 0.001) (see Figure 4B).

We observed a four-fold rise in serum-neutralizing antibody titers on day 35 in 56%, 66%, and 77% of participants in 5, 10, and 20 µg vaccine groups, respectively (Figure 3B). The geometric mean ratios (GMR) and their 95% confidence interval compared with the placebo on day 35 for neutralizing antibody titers were 5.82 (1.46–23.13), 11.12 (2.74–45.09), and 20.70 (5.05–84.76) in study groups, respectively. Neutralizing antibody responses were tightly correlated with anti-S and anti-RBD in all three vaccine groups (see Figure S6 and Table S18).

**Figure 5:**
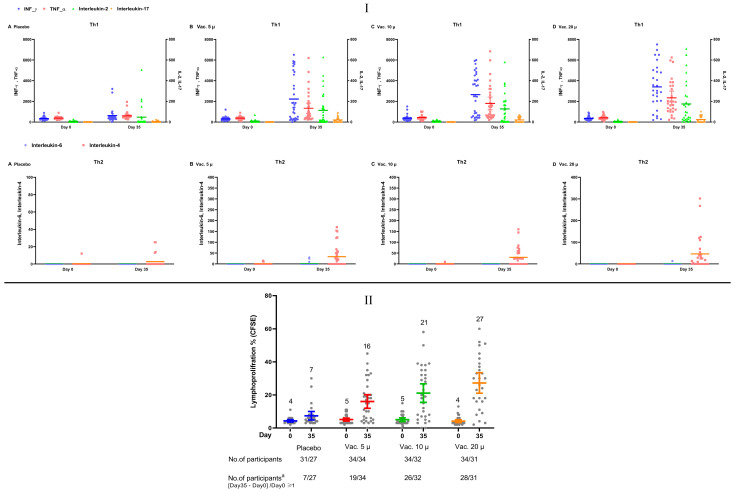
(**I)** Scatter diagram of the ELISA levels (pg/mL) of IFNɣ, TNF-α, IL-2, IL-17, IL-4, and IL-6 (comparing T-helper-1 and -2 pathways) in peripheral blood mononuclear cell (PBMC) extract following stimulation with the S antigen in the intervention groups on day 35 compared to the baseline. Geometric means have been shown on the diagram. (**II**) Scatter diagram of the percentage of lymphoproliferative response in peripheral blood mononuclear cell (PBMC) extract following stimulation with the S1 antigen and inactivated virus (CFSE) in the intervention groups on day 35 compared to the baseline percentage. Means, 95% confidence intervals, and the number of participants in each group with more than 100% change in the percentage of lymphoproliferative response from the baseline are shown in the diagram.

The increase in the percentage of lymphocyte proliferation (measured using the CFSE method) in response to stimulation by the S1 antigen and inactivated virus on day 35 compared with that of day 0 was 3.3%, 11.2%, 17.9%, and 22.9% in the placebo, 5, 10, and 20 µg study groups, respectively (Figure 5B). Following stimulation with the S1 antigen and inactivated virus, IFNɣ, TNF-α, IL-2, and IL-17 (Th1) increased on day 35 compared with those on day 0 in all the vaccine groups (see Figure 5A). The rate of increase was almost similar in the 10 and 20 µg vaccine groups, but it was higher than that of the 5 µg vaccine group. The IL-4 response (Th2) revealed an increase on day 35 compared with day 0 in all the vaccine groups (see Figure A). IL-6 did not change in any of the study groups. The percentage of CD3/CD4 cells remained relatively the same on days 35 and 0 in all the vaccine groups; meanwhile, the percentage of CD3/CD8 cells showed a noticeable increase. Overall, our findings indicated a strong T cell response to all the investigated vaccine strengths with Th1 dominance and an increase in cytotoxic cells (see Figure S7).

## 4. Discussion

We found that the RCP recombinant spike protein COVID-19 vaccine is safe and well-tolerated. Moreover, it does not cause serious adverse reactions in healthy adult volunteers. The incidence of solicited adverse reactions in all the study groups was similar, and no vaccine-related SAEs were seen. The most frequent adverse reaction was a headache, followed by injection site tenderness. We observed antibody responses against both S and RBD antigens in the 10 and 20 µg vaccine groups on day 35, which were four and six times higher than the placebo. These responses were sustained until the end of the study period. Similarly, on day 35, the serum neutralizing antibody response in the 10 and 20 µg vaccine groups was 11 and 21 times higher than that in the placebo group. A strong secretory IgA response to the RBD antigen was observed in the participants two weeks after receiving the intranasal dose, which remained high for over three months. We showed inhibition of ACE2 adhesion using nasopharyngeal mucosal fluid of the recipients of intranasal 10 µg vaccine on day 65. The vaccine also evoked cellular immunity detected with increased lymphocyte proliferation and secretion of IFNγ, TNFα, IL-2, and I-L4 in response to stimulation by the S1 antigen and inactivated virus. The T-helper-1 response was predominant.

The prevalence of local and systemic adverse reactions to the RCP recombinant spike protein COVID-19 vaccine was relatively low, generally mild, and resolved spontaneously. Similar to many other subunit vaccines, local pain and tenderness were at the top of the local reactions list [3,22,23,24]. No case of fever following the injections was detected. This observation was in accordance with the reports in other protein subunit adjuvanted vaccines [24]. The case of chemical pregnancy was thoroughly discussed in the data and by the safety monitoring board. An independent non-member gynecologist advised the DSMB in this regard.

The RCP recombinant spike protein COVID-19 vaccine contains a third intranasal dose. The nano-sized structure of this vaccine [15] allows its direct application on mucosal surfaces, infiltration to epithelial cells, and subsequent stimulation of the relevant cells of the immune system. Therefore, the RCP can switch the antibody class to secretory IgA and provide mucosal immunity, which could potentially block the transmission of COVID-19 [8,14] and was our primary purpose in integrating the intranasal dose into the vaccination program. Given the SARS-CoV-2 pandemic, the prime/boost combination of the two intramuscular (days 0 and 21) and one intranasal dose (day 51) could elicit rapid systemic followed by mucosal immune responses [25,26]. The minimum one-month interval between the intranasal dose and the second intramuscular dose was needed to avoid confronting the immune system with an antigenic load while the specific antibodies were on the rise following the previous load of the same antigen (the two intramuscular doses) [25,27]. We detected a high level of COVID-19-specific anti-RBD IgA antibodies in nasopharyngeal mucosal fluid, which remained high throughout the study. Furthermore, we demonstrated a significant ACE2 adhesion inhibition as a result of intranasal vaccination with the 10 µg vaccine, indicating a strong secretory IgA antibody response with neutralizing capability. Currently, there are 11 other vaccine candidates containing intranasal doses at different phases of clinical trials [7]. This report is the first on the findings from intranasal vaccines in humans.

Furthermore, there were strong antibody responses two weeks after the second injection against various specific COVID-19 antigens (S, S1, S2, NTD, RBD, and N). Antibody against nucleocapsid (N) was measured to differentiate between the immune system responses to wild virus and the vaccine, which showed a negligible contribution of the former to the total antibody response. Despite the negative serological anti-N antibody results, a number of the subjects from the placebo and other vaccinated groups were infected at different time points post-vaccination. However, using a randomized control group receiving a placebo enabled us to account for the contribution of subclinical infections with wild virus in the overall observed antibody responses in either of the vaccine groups by calculating the geometric mean ratio (GMR). Masking the immunology lab operators to the immunogenicity results provided some assurance regarding the unbiased assessment of the immune response. Although comparing the ELISA antibody responses in vaccine groups to the placebo offered some estimates of their magnitude, clinical interpretation of their efficacy remains to be seen. Neutralizing antibody activity was assessed after exposing sera from the participants to the wild virus isolated from the Iranian population at the start of the pandemic in early 2021. The strong and positive correlation between neutralizing antibody titer and ELISA S and RBD-specific antibodies suggested that these two mainly mediate the neutralization.

Specific cellular immunity plays an essential role in COVID-19 inhibition. Interleukins are the first-level cell mediators for the activation of cellular immunity. The results obtained herein proved the development of prominent specific cellular immunity against COVID-19. In this study, Th1 was higher than Th2. Our observed strong specific high-affinity IgG antibody response could be explained by the increase in IL4 secretion (Th2 pathway activation) and transformation of B-cells to plasma cells [28]. Long-lasting immunity as shown by sustained specific antibody response up to 150 days (See Table 3) is the result of the Th1 pathway activation [29,30]. The in vitro increase in lymphocyte proliferation against antigen S1 and inactivated virus showed activation of memory T cells, which were formed with vaccination. The in vitro challenge of peripheral blood mononuclear cells with antigen S1 and inactivated viruses showed that CD8 cells responded more than CD4 cells. This is another reason for immune activation via cytotoxic T cells. Considering lymphocyte proliferation and other results, such as the Th1/Th2 ratio, we can claim that the vaccination system favorably activated cellular immunity. Some slight increases in the level of cytokines in the placebo group could be explained by a few occurrences of subclinical infections as described above.

The study started when some vaccines had received emergency use authorization; however, none of them were available for the Iranian population. Therefore, the national ethics committee allowed us to use a placebo as the comparator in this study. We used a separate group of non-randomized open-label participants for the evaluation of mucosal immunity and unfortunately, appropriate serum specimens were not taken simultaneously from this group.

The RCP vaccine uses recombinant SARS-CoV-2 spike protein in its monomeric (S1 and S2 subunits) and trimeric form (S-Trimer) formulated in the oil-in-water adjuvant [15]. Additional testing of stored serum samples using S1 and S-Trimer antigens showed a negligible (10% Omicron vs. variant in Wuhan) decline in ELISA-specific antibody responses against S-Trimer by changing the SARS-CoV-2 variants over time (see Figure 3C) while the percentage of decline against monomeric S1 was remarkably high (45% Omicron vs. variant in Wuhan). This finding suggests that the inclusion of monomeric S2, a more preserved component of spike protein, and S-Trimer in the structure of the RCP vaccine has made it more resistant to the changing variants of SARS-CoV-2 [15,16,18,19,20]. Furthermore, multiple spike antigens used in building the vaccine allow integration of new antigens from future variants much easier.

We considered the following two points to select one of the three strengths of the RCP vaccine to enter the phase II trial. Firstly, any increase in the antigen should not over-increase the inflammatory factors, such as IL-6. Secondly, given the pandemic situation, cost-effectiveness, and ethical issues, the least amount of antigen providing sufficient immune response is desirable. The results revealed that both the 10 and 20 µg vaccines were safe and could contribute to strong and sustained immune response over the study period. Therefore, we selected the 10 µg vaccine to enter the next trial phases.

The RCP vaccine is safe and creates strong and durable humoral and cellular immune responses in its 10 and 20 µg /200 µL vaccine strengths. The 10 µg intranasal dose results in a robust secretory IgA response with neutralizing capabilities that is necessary for good mucosal protection.

## 5. Conclusions

RCP vaccine is safe and induces robust and long-lasting humoral and cellular immunity and good mucosal immune response in its 10 µg /200 µL vaccine strengths.

## Figures and Tables

**Table 1:** Predefined time schedule of this study.

	Screening	0	7	14	21	28	35	51	58	65	150
Nasal swab for COVID-19 PCR test ^a^	×										
Visit to the study center and physical examination	×	×	×	×	×	×	×	×	×	×	×
Psychological assessment	×										
Blood sample: screening ^b^	×										
Vaccination		×			×			×			
Blood sample: safety ^c^			×			×			×		
Blood sample: humoral immunogenicity		×	×	×	×	×	×			×	×
Blood sample: cellular immunogenicity		×					×			×	×
Blood sample: VNT		×					×			×	×
Immediate and solicited local and systemic reactions		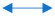			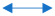			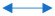			
Unsolicited adverse events		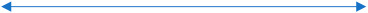									
Medically attended adverse events		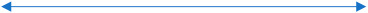									

^a^ Participants underwent nasopharyngeal swab testing for SARS-CoV-2 whenever they reported symptoms suggestive of possible infection. ^b^ Tests included CBC, ESR, CRP, sodium, potassium, magnesium, phosphorous, albumin, total protein, BUN, creatinine, PT, PTT, alkaline phosphatase, ALT, AST, total bilirubin, LDH, urine protein, urine glucose, U/A RBC, HbA1c, IgM and IgG for SARS-CoV-2, HBsAg, HBcAb, Anti HCVAb, HIV, and beta HCG for women. ^c^ Tests included CBC, ESR, CRP, alkaline phosphatase, ALT, AST, total bilirubin, LDH, urine protein, urine glucose, and U/A RBC.

**Table 2:** Baseline comparison of the 120 randomized participants.

	Placebo *n* = 30	Vac. 5 µ *n* = 30	Vac. 10 µ *n* = 30	Vac. 20 µ *n* = 30	Total *n* = 120
Gender, *n* (%)					
Male	20 (66.67)	26 (86.67)	24 (80.0)	21 (70.0)	91 (75.83)
Female	10 (33.33)	4 (13.33)	6 (20.0)	9 (30.0)	29 (24.17)
Age					
Mean (SD)	35.76 (6.69)	36.16 (8.03)	37.3 (6.94)	35.03 (6.65)	36.06 (7.06)
Median (min–max)	36.5 (21–54)	36 (21–49)	36.5 (23–55)	35.5 (23–55)	36.0 (21–55)
Body-mass index					
Mean (SD)	26.18(3.52)	25.99 (3.81)	25.89 (3.46)	25.72 (4.65)	25.94 (3.84)
Median (min–max)	26 (18.5–33)	25.5 (19.2–34.2)	25.2 (18.7–32.4)	25.85 (18.7–34.7)	25.7 (18.5–34.7)
Smoking, *n* (%)					
Current Smoking	5 (16.67)	8 (26.67)	8 (26.67)	8 (26.67)	29 (24.17)
Past Smoking	3 (10.0)	2 (6.67)	6 (20.07)	2 (6.67)	13 (10.83)
Never Smoking	22 (73.33)	20 (66.67)	16 (53.33)	20 (66.67)	78 (65.0)
Education, *n* (%)					
Diploma	5 (16.67)	5 (16.67)	4 (13.33)	4 (13.33)	18 (15.0)
Diploma plus	3 (10.0)	2 (6.67)	3 (10.0)	0 (0.0)	8 (6.67)
Bachelor	8 (26.67)	11 (36.67)	10 (33.33)	12 (40.0)	41 (34.17)
Master	9 (30.0)	10 (33.33)	10 (33.33)	10 (33.3)	39 (32.50)
Doctoral and above	5 (16.67)	2 (6.67)	3 (10.0)	4 (13.33)	14 (11.67)
Job, *n* (%)					
Unemployed/Retired	2 (6.67)	4 (13.33)	0 (0.0)	1 (3.33)	7 (5.83)
Government employee	9 (30.0)	9 (30.0)	9 (30.0)	10 (33.33)	37 (30.83)
Private employee	7 (23.33)	7 (23.33)	12 (40.0)	9 (30.0)	35 (29.17)
Private work	7 (23.33)	8 (26.67)	7 (23.33)	8 (26.67)	30 (25.0)
Housewife	5 (16.67)	2 (6.67)	2 (6.67)	2 (6.67)	11 (9.17)

**Table 3:** Geometric mean ratio and 95% CI of specific antibody responses (AUC) to S, S1, S2, RBD, and NTD antigens (measured using ELIZA) and neutralizing antibody response (measured using cVNT) in the intervention groups over the predefined study time schedule.

		Baseline ^a^	Day 7	Day 14	Day 21 ^a^	Day 28	Day 35	Day 65	Day 150	Seroconversion Rate ^b^ on Day 35 (%) and 95% CI ^c^
GMR_AUC_ (95% CI)										
Anti S antibody	Placebo	1	1	1	1	1	1	1	1	14.8 (4.2–33.7)
	Vac. 5 µg	1.28 (1.08–1.52)	1.36 (1.00–1.84)	1.50 (0.99–2.28)	1.50 (0.92–2.45)	1.96 (1.03–3.73)	3.44 (1.66–7.14)	3.55 (1.69–11.25)	3.20 (1.70–9.03)	55.9 (37.9–72.8)
	Vac. 10 µg	1.01 (0.85–1.20)	1.21 (0.90–1.64)	1.29 (0.85–1.96)	1.57 (0.96–2.57)	1.97 (1.03–3.78)	4.19 (2.00–8.79)	3.68 (1.61–15.33)	5.60 (1.68–27.39)	65.6 (46.8–81.4)
	Vac. 20 µg	0.90 (0.75–1.07)	0.93 (0.69–1.26)	1.03 (0.68–1.56)	1.15 (0.70–1.89)	1.96 (1.02–3.78)	6.12 (2.90–12.89)	5.15 (1.66–25.79)	5.61 (1.70–25.28)	80.0 (61.4–92.3)
Anti S1 antibody	Placebo	1	1	1	1	1	1	1	1	14.8 (4.2–33.7)
	Vac. 5 µg	1.28 (1.08–1.52)	1.36 (1.0–1.84)	1.50 (0.99–2.28)	1.50 (0.92–2.45)	1.96 (1.03–3.73)	3.46 (1.67–7.18)	2.70 (0.73–9.97)	3.04 (0.87–10.59)	47.0 (29.8–64.9)
	Vac. 10 µg	1.01 (1.08–1.20)	1.21 (1.14–1.64)	1.24 (0.82–1.88)	1.57 (0.96–2.57)	1.97 (1.03–3.78)	4.22 (2.01–8.84)	2.87 (0.87–9.52)	5.61 (1.64–19.17)	62.5 (43.7–78.9)
	Vac. 20 µg	0.90 (0.75 -1.07)	0.93 (0.69–1.26)	1.03 (0.68–1.56)	1.15 (0.70–1.89)	1.96 (1.02–3.78)	6.15 (2.92–12.97)	4.20 (1.19–14.84)	4.95 (1.40–17.45)	80.0 (61.4–92.3)
Anti S2 antibody	Placebo	1	1	1	1	1	1	1	1	11.1 (2.3–29.1)
	Vac. 5 µg	1.46 (1.09–1.95)	1.49 (1.05–2.10)	1.65 (1.05–2.61)	1.73 (1.02–2.93)	2.17 (1.10–4.30)	4.04 (1.90–8.59)	2.69 (1.67–6.82)	3.17 (1.62–10.91)	47.0 (29.8–64.9)
	Vac. 10 µg	1.07 (0.80–1.44)	1.26 (0.89–1.77)	1.39 (0.88–2.20)	1.67 (0.99–2.84)	2.17 (1.09–4.34)	4.89 (2.28–10.50)	2.94 (1.60–9.78)	4.40 (1.61–22.65)	65.6 (46.8–81.4)
	Vac. 20 µg	0.87 (0.65–1.16)	0.95 (0.67–1.34)	1.07 (0.68–1.68)	1.25 (0.73–2.12)	2.14 (1.06–4.30)	6.98 (3.23–15.08)	4.05 (1.64–16.61)	4.68 (1.63–23.81)	83.3 (65.3–94.3)
Anti RBD antibody	Placebo	1	1	1	1	1	1	1	1	14.8 (4.2–33.7)
	Vac. 5 µg	1.38 (1.13–1.68)	1.45 (1.06–1.98)	1.57 (1.00–2.47)	1.43 (0.87–2.34)	1.99 (1.06–3.76)	3.40 (1.63–7.07)	2.69 (1.68–6.82)	3.38 (1.68–10.38)	55.9 (37.9–72.8)
	Vac. 10 µg	1.05 (0.86–1.27)	1.21 (0.89–1.64)	1.30 (0.83–2.05)	1.57 (0.96–2.57)	1.88 (0.99–3.58)	4.07 (1.94–8.55)	3.04 (1.60–10.49)	5.98 (1.67–32.79)	62.5 (43.7–78.9)
	Vac. 20 µg	0.91 (0.75–1.11)	1.01 (0.74–1.37)	1.05 (0.67–1.65)	1.13 (0.69–1.86)	2.01 (1.05–3.84)	5.88 (2.78–12.44)	4.22 (1.65–17.99)	5.75 (1.69–27.94)	83.3 (65.3–94.3)
Anti NTD antibody	Placebo	1	1	1	1	1	1	1	1	14.8 (4.2–33.7)
	Vac. 5 µg	1.31 (1.08–1.60)	1.40 (1.03–1.91)	1.54 (1.01–2.37)	1.50 (0.91–2.47)	1.93 (1.01–3.67)	3.24 (1.51–6.93)	2.30 (1.71–4.71)	3.44 (1.68–10.70)	50.0 (32.4–67.6)
	Vac. 10 µg	1.06 (0.87–1.29)	1.21 (0.89–1.64)	1.31 (0.85–2.00)	1.62 (0.98–2.66)	1.98 (1.03–3.81)	4.23 (1.95–9.15)	3.01 (1.63–9.49)	5.75 (1.67–30.27)	65.6 (46.8–81.4)
	Vac. 20 µg	0.93 (0.76–1.13)	1.01 (0.74–1.37)	1.12 (0.73–1.71)	1.24 (0.75–2.05)	2.11 (1.09–4.09)	6.67 (3.07–14.52)	4.12 (1.68–15.49)	5.52 (1.69–25.79)	83.3 (65.3–94.3)
Neutralyzing antibody cVNT	Placebo	1	-	-	-	-	1	1	1	14.8 (4.2–33.7)
	Vac. 5 µg	1.08 (0.98–1.20)	-	-	-	-	5.82 (1.46–23.13)	0.95 (0.03–27.05)	0.84 (0.02–28.22)	58.8 (40.7–75.3)
	Vac. 10 µg	1.00 (0.91–1.10)	-	-	-	-	11.12 (2.74–45.09)	31.11 (1.46–663.67)	6.58 (0.17–259.11)	68.7 (49.9–83.9)
	Vac. 20 µg	1.00 (0.91–1.10)	-	-	-	-	20.70 (5.05–84.76)	17.46 (0.47–652.38)	1.68 (0.03–82.75)	83.3 (65.3–94.3)

^a^ Participants received intramuscular vaccine/placebo injections on days 0 and 21, followed by an intranasal spray of vaccine/placebo on day 51 (please also see Table 1). ^b^ The seroconversion rate in each placebo/vaccine group was calculated by dividing the number of participants experiencing a four-fold increase in antibody titer by the total number of participants tested. ^c^ The 95% confidence intervals for proportions were calculated using the Clopper–Pearson method. Comparing the confidence intervals of seroconversion rates in the vaccine groups with the placebo group shows a statistically significant difference between the responses in the groups. (The difference is statistically significant when the confidence intervals do not cross).

## Data Availability

We support sharing of the individual participant data. The individual participant data that underlie the results reported in this article after de-identification (text, tables, figures, and appendixes) will be shared. Because this is the study’s final report, individual participant data and supporting clinical documents, including study protocol, statistical analysis plan, and the informed consent form, will be available immediately following publication for at least one year. Researchers who provide a scientifically sound proposal will be allowed access to the individual participant data. Proposals should be sent to the corresponding author. Based on scientific merit, these proposals will be reviewed and approved by the funder, investigator, and collaborators. Data requesters will need to sign a data access agreement to gain access.

## Supplementary Materials

The following supporting information can be downloaded at: https://www.mdpi.com/article/10.3390/vaccines11020455/s1, Supplementary File S1: Protocol Phase I, II [3,7,9,10,11,12,31,32,33,34,35,36]; Table S1: FDA toxicity grading scale for vital sign abnormalities; Table S2: FDA toxicity grading scales for solicited local and systemic adverse events; Table S3: FDA toxicity grading scales for clinical laboratory abnormalities; Table S4: World Health Organisation-Uppsala Monitoring Centre causality assessment scale; Table S5: Predefined time schedule of the study; Table S6: Demographic characteristics of the sentinel participants; Table S7: Geometric mean ratio and 95% CI of specific antibody responses (AUC) to S, S1, S2, RBD and NTD antigens (variant in Wuhan) in the intervention groups over the predefined study time schedule; Table S8: Serum levels of specific antibodies against S, S1, S2, RBD, NTD and N antigens (variant in Wuhan) in trial participants at enrollment; Table S9: Number and percentages of subjects experiencing solicited local and systemic adverse events vaccination dose, by FDA toxicity grade; Table S10: Number and percentages of subjects experiencing solicited systemic adverse events after third dose of vaccine, by FDA toxicity grade; Table S11: List of adverse events, their grades and causal relationship with the intervention received (sorted by grade) during the six-month follow-up period; Table S12: List of patients with positive COVID-19 PCR test result during the follow up period; Table S13: Abnormal vital signs during 3 h after dose 1 and 2; Table S14: Number and percentages of subjects experiencing laboratory abnormalities by FDA toxicity grade; Table S15: Geometric means of IgG antibody responses (presented as area under the curve, AUC) against S antigen (variant in Wuhan) in the intervention groups over the predefined study time schedule; Table S16: Geometric means of IgG antibody responses (presented as area under the curve, AUC) against S1 antigen (variant in Wuhan) in the intervention groups over the predefined study time schedule; Table S17: Geometric means of IgG antibody responses (presented as area under the curve, AUC) against S2 antigen (variant in Wuhan) in the intervention groups over the predefined study time schedule; Table S18: Geometric means of IgG antibody responses (presented as area under the curve, AUC) against RBD antigen (variant in Wuhan) in the intervention groups over the predefined study time schedule; Table S19: Geometric means of IgG antibody responses (presented as area under the curve, AUC) against NTD antigen (variant in Wuhan) in the intervention groups over the predefined study time schedule; Table S20: Geometric means of IgG antibody responses (presented as area under the curve, AUC) against N antigen (variant in Wuhan) in the intervention groups over the predefined study time schedule; Table S21: Geometric mean titer for neutralizing antibody titer the over the predefined study time schedule; Figure S1: Geometric means of IgG antibody responses (presented as area under the curve, AUC) against S antigen in the intervention groups over the predefined study time schedule; Figure S2: Geometric means of IgG antibody responses (presented as area under the curve, AUC) against S1 antigen in the intervention groups over the predefined study time schedule; Figure S3: Geometric means of IgG antibody responses (presented as area under the curve, AUC) against S2 antigen in the intervention groups over the predefined study time schedule; Figure S4: Geometric means of IgG antibody responses (presented as area under the curve, AUC) against RBD antigen in the intervention groups over the predefined study time schedule; Figure S5: Geometric means of IgG antibody responses (presented as area under the curve, AUC) against NTD antigen in the intervention groups over the predefined study time schedule; Figure S6: Scatter plots illustrating the correlation between neutralizing antibody responses and specific IgG ELISA antibody responses (AUC) at 2 weeks after the second dose (day 35) in the intervention groups. Nonparametric Spearman correlation estimates have been shown on the diagrams; Figure S7: Scatter diagram of changes in percentage of CD3, CD4 and CD3, CD8 (cytotoxic) T cells in response to stimulation by S antigen measured by flow cytometry in peripheral blood mononuclear cell (PBMC) extract in the intervention groups at the day 35 compared to the baseline. Percentage means have been shown on the diagram. [33,34,35]
