# Supplementary material for: Immunogenicity and Safety of a Combined Intramuscular/Intranasal Recombinant Spike Protein COVID-19 Vaccine (RCP) in Healthy Adults Aged 18 to 55 Years Old: A Randomized, Double-Blind, Placebo-Controlled, Phase I Trial
Source: Vaccines (Basel). 2023 Feb 16;11(2):455. doi: 10.3390/vaccines11020455 (PMC9961243; doi:10.3390/vaccines11020455)

Immunogenicity and safety of a recombinant spike protein COVID-19 vaccine (RCP) in healthy adults aged 18 to 55 years old: a randomized, double-blind, placebo controlled, phase 1 trial and Phase II in Healthy Adults Aged 18-70 Years; Parallel 2 Arms Design (Adjuvant Only and Selected Dose of Vaccine): A Randomized, Double Blind, Clinical Trial

Table of Contents

1. PROTOCOL SUMMERY 6

1.1. SYNOPSIS 6
1.1.1. Protocol Registration 6
1.1.2. Protocol amend 6
1.1.3. Short Title 6
1.1.4. Rational 6
1.1.5. Objectives, Estimands, and Endpoints 6
1.1.6. Overall Design 7
1.1.7. Number of Participants 8
1.1.8. Intervention Groups and Duration 8
1.1.9. Data Monitoring Committee or Other Independent Oversight Committee 8
1.1.10. Statistical method 8

1.2. Schedule of activity 9
Phase I 9
Phase II 9

2. INTRODUCTION 9

2.1. Study Rationale 9

2.2. Background 10

2.3. Benefit/ Risk assessment 10
2.3.1. Risk assessment 10
2.3.2. Benefit Assessment 11
2.3.3. Overall Benefit/Risk Conclusion 11

2.4. Protocol Amendments 11

3. OBJECTIVES, ESTIMANDS, AND ENDPOINTS 14
For Phase I 14
For Phase II 15

4. STUDY DESIGN: 15

4.1. Overall Design 15

4.2. Scientific Rationale for Study Design 16

.5 STUDY POPULATION 16

5.1. Inclusion Criteria 16
5.1.1. Inclusion Criteria for Phase I 16

5.1.2. Inclusion Criteria in Phase II 17

5.2. Exclusion Criteria 17
5.2.1. Exclusion Criteria in Phase I 17
5.2.2. Exclusion Criteria in Phase II 18

6. STUDY INTERVENTION 18

6.1. Storage, handling and preparation 18
6.1.1. Administration route 18
6.1.2. Adherence monitoring strategies 19

6.2. Measures to Minimize Bias: Randomization and Blinding 19
6.2.1. Allocation to Study Intervention 19
6.2.2. Blinding of Site Personnel and Sponsor 19
6.2.3. Un-blinding 19

6.3. Study Intervention Compliance 19

7. WITHDRAWAL CRITERIA.... 19

7.1. Withdrawal criteria in Phase I 19

7.2. Withdrawal criteria in Phase II 20

8. PAUSE RULES.... 20

9. STUDY PROCEDURES.... 20

9.1. Study conduct site 20

9.2. Recruitment Strategy....20

9.3. Screening....20
9.3.1. Digital screening 21

9.4. Lab Assessments....21

9.5. Sentinel participants....22

9.6. Participant visit plans....22
9.6.1. Participant visit plan in Phase I 22
9.6.2. Participant visit plans in Phase II 25

10. DATA COLLECTION, MANAGEMENT, AND ANALYSIS.... 28

10.1. Data collection methods 28

10.2. CRF booklet for screening **28**
10.3. CRF booklet for assigned participants 28
10.4. Diary card 29
10.5. Data management **29**
10.6. Data monitoring **29**
11. MONITORING 29
11.1. Harms **29**
11.2. Safety Reporting Guidelines **29**
11.2.1. Adverse Event (AE) 29
11.2.2. Serious Adverse Event / Reaction (SAE) 30
11.2.3. Suspected Unexpected Serious Adverse Reaction (SUSAR) 30
.11.2.4 Medically Attended Adverse Events (MAAEs) 31
12. ETHICS AND DISSEMINATION 31
12.1. Research Ethics Approval **31**
12.2. Protocol Amendments **31**
12.3. Informed Consent **31**
12.4. Compensation **31**
12.5. Confidentiality **31**
12.6. Dissemination policy **31**
12.7. Additional Considerations **31**
12.7.1. Vaccine/IMP- related complications 31
13. STATISTICAL METHODS 32
13.1. Sample Size **32**
13.2. Definition **32**
13.2.1. Safety population 32
13.2.2. Immunogenicity population 32
13.3. Analysis approach **32**
13.4. Analysis plan **32**
13.5. Missing Data **33**

13.6. Interim analysis **33**

REFERENCES 34

14. APPENDIX 35

1. Protocol Summery

1.1. Synopsis

1.1.1. Protocol Registration

This title was approved by the National Ethics Committee and then registered in Iranian Registry of Clinical Trials (IRCT) with the code IRCT20201214049709N1 and IRCT20201214049709N2.

1.1.2. Protocol amend

Amendment on April 6, 2021.
Amendment on September 6, 2021.
Amendment on January 16, 2022

1.1.3. Short Title

A Phase I and II Study to Evaluate Safety and Immunogenicity of Razi SARS-CoV-2 recombinant Spike Protein (rSARS-COV-2) Vaccine in Healthy Adults, a Randomized, Double Blind, Clinical Trial

1.1.4. Rational

This world has experienced three horrible emerging diseases, so far in 21 century which are related to novel coronavirus: Severe acute respiratory syndrome (SARS), Middle East respiratory syndrome (MERS) ,and coronavirus disease 2019 (COVID-19). COVID-19 emerged at the end of 2019 in Wuhan, China. On 11 March 2020, WHO characterized the COVID-19 situation as a pandemic. COVID-19 is spread almost all over the world.
Global collaborations and research efforts among the scientific community have led to 141 vaccines, in clinical phases. The most used method (34%) is the Protein subunit among all vaccines in the clinical phase. Recombinant subunit vaccines have advantages of fewer potential safety concerns and lower production cost and they need an adjuvant to induce required immune response. Currently licensed COVID-19 vaccines are all designed for intramuscular (IM) immunization. Mucosal primary target cells need secretory IgA (S IgA) to effectively prevent viral replication within air ways, which generally requires a mucosal route of vaccination. Presently, although not licensed, various intranasal vaccines against SARS-CoV-2 are under intensive investigation, with 12 candidates reaching clinical trials at different phases.
Razi Vaccine and Serum Research Institute with 95 years experience in research and biological production has developed a recombinant vaccine candidate using a platform of subunit protein. This vaccine candidate is based on subunit protein of a cocktail of spike receptor antigens. This vaccine is one of 12 candidate vaccines with an intranasal dose, in that it has two IM doses on days 0 and 21 and another intranasal dose on day 51.

1.1.5. Objectives, Estimands, and Endpoints

For Phase I

**Objective**	**Estimand**	**Endpoints**
Primary		
To describe the safety profile of solicited adverse events of RCP in healthy adults after the first, second, and the third dose of RCP recombinant spike protein COVID-19 vaccine.	In participants who received at least one dose of study intervention, the percentage of participants reported:<br>• Abnormal vital signs and anaphylactic reactions in the first 3 hours after vaccination<br>• Local adverse events within the first week after vaccination<br>• Systemic adverse events within the first week after vaccination<br>• Abnormal results of laboratory findings(on days 7, 28, and 58)	• Immediate adverse reaction<br>• Local reactions (pain, tenderness, erythema/redness, swelling/induration, itching)<br>• Systemic events (fever, nausea and vomiting, diarrhea, headache, fatigue, muscle pain)<br>• Biochemistry, hematology, and urine tests
**Secondary**		
To describe the unsolicited AE elicited by RCP	• Number and percentage of:<br>• Severe Adverse event (SAEs) up to six months after first dose	• SAE<br>• SUSAR<br>• MAAEs

recombinant spike protein COVID-19 vaccine.	• Suspected Unexpected Serious Adverse Reaction up to six month<br>• Medically Attended Adverse Events	
To describe the immune responses	• Geometric Mean Area Under the Curve (GM AUC) of specific IgG antibodies.<br>• Geometric Mean Ratio (GMR)<br>• Geometric Mean Fold Increase (GMFI)<br>• Geometric Mean Fold Ratio (GMFR)<br>• Seroconversion rate (the proportion of the individuals whose serum IgG levels have two fold or more using the ELISA method)	• Specific IgG antibodies against S<br>• Specific IgG antibodies against S1<br>• Specific IgG antibodies against S2<br>• Specific IgG antibodies against RBD<br>• Specific IgG antibodies against NTD
	• GM AUC of specific IgA<br>• GMR of IgA Secretory activity levels	• Specific IgA antibodies against RBD
	• Geometric Mean Titers (GMTs) at each time point<br>• Geometric Mean Ratio (GMR) of<br>• The proportion of participants achieving ≥4-fold rise from before vaccination to each subsequent time point after vaccination	• Neutralizing antibody activity
	• Counting the number of CD cells<br>• Level of Gamma interferon (IFNγ), tumor necrosis factor (TNFα), and interleukin IL-2, 4, 6, and 17	• CD3, CD4, and CD8 cells and joint calculation of CD3 and CD4 and CD3 and CD8<br>• Gamma interferon (IFNγ), tumor necrosis factor (TNFα), and interleukin IL-2, 4, 6, and 17 were assessed by ELISA
	• Number and percentage of Covid-19 disease occurrence two weeks after second vaccine dose	• Occurrence of clinical Covid-19

For Phase II

**Objective**	**Estimand**	**Endpoints**
Primary		
To describe the safety profile of solicited adverse events of RCP in healthy adults after the first, second, and the third dose of RCP recombinant spike protein COVID-19 vaccine.	In participants who received at least one dose of study intervention, the percentage of participants reporting:<br>• Abnormal vital signs and anaphylactic reactions in first 2 hours after vaccination<br>• Local adverse events within the first week after vaccination<br>• Systemic adverse events within the first week after vaccination	• Immediate adverse reaction<br>• Local reactions (pain, tenderness, erythema / redness, swelling / induration, itching)<br>• Systemic events (fever, nausea and vomiting, diarrhea, headache, fatigue, muscle pain)
To describe the immune response	• Geometric Mean Area Under the Curve (GM AUC) of specific IgG antibodies.<br>• Geometric Mean Ratio (GMR)<br>• Geometric Mean Fold Increase (GMFI)<br>• Geometric Mean Fold Ratio (GMFR)<br>• Seroconversion rate (the proportion of the individuals whose serum IgG levels have two fold or more using the ELISA method)	• Specific IgG antibodies against S<br>• Specific IgG antibodies against S1<br>• Specific IgG antibodies against S2<br>• Specific IgG antibodies against RBD<br>• Specific IgG antibodies against NTD
**Secondary**		
To describe the unsolicited AE<br>elicited by RCP recombinant spike protein COVID-19 vaccine	Number and percentage of:<br>• Abnormal results of laboratory findings<br>• Severe Adverse event (SAEs) up to six months after first dose<br>• Suspected Unexpected Serious Adverse Reaction up to six month<br>• Medically Attended Adverse Events	• Biochemistry, hematology, and urine tests<br>• SAE<br>• SUSAR<br>• MAAEs
	• Number and percentage of COVID-19 disease occurrence two weeks after second vaccine dose	• Occurrence of clinical Covid-19
To describe the immune responses	• Geometric Mean Titers (GMTs) at each time point<br>• Geometric Mean Ratio (GMR)<br>• Proportion of participants achieving ≥4-fold rise from before vaccination to each subsequent time point after vaccination	• Neutralizing antibody activity
	• Counting the number of CD cells<br>• Level of Gamma interferon (IFNγ), tumor necrosis factor (TNFα), and interleukin IL-2, 4, 6, and 17	• CD3, CD4 and CD8 cells and joint calculation of CD3 and CD4 and CD3 and CD8<br>• Gamma interferon (IFNγ), tumor necrosis factor (TNFα), and interleukin IL-2, 4, 6, and 17 assessed by ELISA

1.1.6. Overall Design

This study is a Phase 1/2, randomized, placebo-controlled, double-blind, dose-finding study in healthy individuals.

The study consists of 2 parts: Phase 1: identifying vaccine safety profile and dose-finding; Phase 2: parallel RCT for assessing the immunogenicity and safety of RCP recombinant spike protein COVID-19 vaccine.

- In Phase I, three different doses of vaccine and placebo (all in 3 dose : days 0, 21 and 51) will be compared with each other, and in Phase II, the selected dose will be compared with the placebo (both in 3 dose : days 0, 21 and 51)
- Included ae groups are 18 to 55 years old in Phase I and 18 to 70 in Phase II

1.1.7. Number of Participants

In Phase I, 153 participants will be recruited in three stages.

- Thirteen people as the sentinel group (one for adjuvant only and three for each vaccine dose)
- One hundred and twenty individuals will be randomly assigned into four groups of 30, receiving a placebo and the three strengths of the vaccine
- Twenty participants will receive 2 IM doses of the selected vaccine and either an intranasal vaccine (10-µg/200µl) or placebo.

In Phase II, 500 participants will randomly receive the selected dose of vaccine or placebo.

1.1.8. Intervention Groups and Duration

In Phase I, the study will evaluate the 3-dose (day 0, 21, and 51) schedule of vaccines of doses 5, 10, and 20-µg/200µl of a recombinant spike protein COVID-19 vaccine (RCP). Furthermore, In Phase II, a comparison of the selected dose and placebo with the same schedule as Phase I will consider.

1.1.9. Data Monitoring Committee or Other Independent Oversight Committee

A data and safety monitoring board (DSMB) will supervise the conduct, data collection, and safety outcomes. This committee will be included members from the National Ethics Committee (NEC), the Ministry of Health's Food and Drug Organization (FDO), the Communicable Disease Control (CDC) department, and several independent members, including specialists in internal medicine, infectious disease, pharmacotherapist, and immunology and allergy. There will be at least two non-voting members representing the sponsor.

1.1.10. Statistical method

The sample size is based on practical considerations and expert opinion and not standard formulas and statistical power calculations. In the present study, there are four different vaccine / IMP groups (n= 30 people per group). In addition, 13 people entered the study as sentinel participants, and 20 non-randomized open-labeled to assess secretory IgA. Therefore, in total, the sample size in phase I will be 153 people.

The sample size in phase II of the study is predicted to be 500 people, which is again based on practical considerations and expert opinion.

All candidates who received a single IMP / vaccine dose in the study will be considered as a safety population.

The main analysis approach in this study will be intention to treat (ITT).Moreover the results of the per-protocol analysis will be extracted and compared with the results of the main approach.

For analysis, first, a descriptive analysis of the study variables will be carried out. All variables collected at baseline will be compared in four groups in phase I and two groups in phase II to ensure the random assignment. The collected data will be sorted and then analyzed using SPSS and STATA statistical software. P-value<0.05 will be considered as the significance level in all tests. For Safety and immunogenicity data, descriptive analysis will first be conducted. These data will then be compared into four groups. Chi-square test and, if necessary, Fisher's Exact test will be used to compare ratios. The immunogenicity findings will be analyzed by logarithmically converting the measured titers into geometric mean titers. Independent t-test will be used to compare continuous quantitative data with normal distribution in the two groups and non-parametric Mann-Whitney U test will be used to compare quantitative rank data with non-normal distribution.

The "serum level using geometric mean" variable is the geometric mean of the antibody titer against Spike antigen, which will be compared in the control group with the vaccine groups. The "seroconversion rate" variable will be calculated and compared by evaluating the fold increase in serum levels. The seroconversion rate is defined as the proportion of those whose serum IgG levels have quadrupled or increased using the ELISA method. The number and percentage of these people in the vaccine groups will be compared with the control group.

No imputation will be performed for missing data. Interim analysis will be performed on all **participants** in phase I 14 days after the second vaccine dose (35 days after receiving the first dose). Based on the results of this analysis, a decision will be made regarding the start of phase II.

After phase II (the selected dose) starts, an interim analysis will be performed two weeks after the second vaccine dose like phase I. The above analysis will be carried out with the difference when the first 150 people (30%) of the participants finish their 35th-day visit.

1.2. Schedule of activity

Phase I

Activity	Screening	Day 0	Day 7	Day 14	Day 21	Day 28	Day 35	After interim analysis, a decision will be made regarding the start of the phase II from the Food and Drug Administration.	Day 51	Day 58	Day 65	Day 150	Day 160
Obtaining informed consent	☑												
Evaluation of inclusion and exclusion criteria	☑												
Random allocation		☑											
β-hCG pregnancy test in women	☑	☑			☑				☑				
Vaccine / IMP administration		☑			☑				☑				
Physical examination	☑	☑	☑	☑	☑	☑	☑		☑	☑	☑	☑	
Face-to-face assessment of complications		☑	☑	☑	☑	☑	☑		☑	☑	☑	☑	
Telephone assessment of complications		☑ daily			☑ daily				☑ daily		☑ monthly		
Evaluation of SAEs, SUSARs, MAAEs<br>Follow up on emergencies				☑							☑		
Laboratory safety assessment	☑		☑			☑				☑			
Evaluation of serum specific IgG antibodies levels		☑	☑	☑	☑	☑	☑		☑	☑	☑	☑	
Evaluation of neutralizing antibody activity		☑					☑			☑	☑	☑	
Cell-mediated immunity assessment		☑					☑			☑	☑	☑	

An unplanned potential COVID-19 illness visit and unplanned potential COVID-19 convalescent visit are required at any time from day 0 up to six months when COVID-19 is suspected.

Phase II

An unplanned potential COVID-19 illness visit and unplanned potential COVID-19 convalescent visit are required at any time from day 0 up to six month COVID-19 is suspected.

Activity	Screening	Day 0	Day 7	Day 21	Day 28*	Day 35	After interim analysis, a decision will be made regarding the start of the phase II from the Food and Drug Administration.	Day 51	Day 58*	Day 90	Day 180
Obtaining informed consent	☑										
Evaluation of inclusion and exclusion criteria	☑										
Random allocation		☑									
β-hCG pregnancy test in women	☑	☑		☑				☑			
Vaccine / IMP administration		☑		☑				☑			
Physical examination	☑	☑	☑	☑	☑	☑		☑	☑	☑	☑
Face-to-face assessment of complications		☑	☑	☑	☑	☑		☑	☑	☑	☑
Telephone assessment of complications		☑ daily		☑ daily				☑ daily		☑monthly	
Evaluation of SAEs, SUSARs, MAAEs<br>Follow up on emergencies				☑					☑		
Laboratory safety assessment	☑		☑		☑				☑		
Evaluation of serum specific IgG antibodies levels		☑		☑		☑				☑	☑
Evaluation of neutralizing antibody activity		☑				☑				☑	☑
Cell-mediated immunity assessment		☑				☑				☑	☑

*Laboratory safety assessment, face-to-face assessment of complications and examinations in these days will be carried out only for people over 55 years old. In addition, the assessment of complications for people under 55 years of age will be carried out remotely.

An unplanned potential COVID-19 illness visit and unplanned potential COVID-19 convalescent visit are required at any time from day 0 up to six months when COVID-19 is suspected.

2. Introduction

RCP recombinant spike protein COVID-19 is a subunit vaccine. A cocktail of spike receptor antigens in an oil-in-water adjuvant, Razi Adjuvant System-01 (RAS-01), has been used to formulate this vaccine.

2.1. Study Rationale

The purpose of this study is to describe the safety, tolerability, finding the best dose and immunogenicity of RCP recombinant spike protein COVID-19 as a subunit vaccine.

2.2. Background

COVID-19 (coronavirus disease, 2019) is a novel viral disease caused by the severe acute respiratory syndrome coronavirus 2 (SARS-CoV-2). COVID-19 was first reported in December 2019 in Wuhan, China. Because of the disease severity, alarming spread level, number of affected countries, and number of deaths, the World Health Organization (WHO) declared the COVID-19 pandemic on 11 March 2020. SARS-CoV-2) infection is being considered a significant public health concern. Since the advent of COVID-19 up to 4 February 2022, nearly 390 million cases of COVID-19 have been reported, including more than 5.7 million deaths.
The rapid spread of COVID-19 globally has created many problems. COVID-19 is a result of 8.8% of global lost working hours in 2020. National and international economic damage, psychological and mental disorders, travel restrictions, additional pressure on health care workers and inducing psychological disorders on their school and university closing, and the prohibition on public gatherings are just a part of the known damages of COVID-19(1)[1].
Developing and using proper and safe vaccines is one of the best ways to prevent and control diseases.
Global collaborations and research efforts among the scientific community have led to 144 vaccines in clinical phases with eight different platforms. Among all vaccines in the clinical phase, the most used method (33%) is the Protein subunit (2). The glycosylated spike (s) is considered the main viral antigenic target, a trimeric protein. S antigen consists two subunits (S1 and S2) and is essential for viral binding, fusion and uptake into mammalian cells(3). Recombinant vaccines have the advantages of fewer potential safety concerns and lower production cost, but they need an adjuvant to induce the required immune response (4).
However, injectable vaccines provide humoral and cellular immunity; they have limited mucosal protection. Mucosal primary target cells need secretory IgA to effectively prevent viral replication within airways, which generally requires a mucosal route of vaccination (5). Intranasal vaccination has been reported as an effective route for reducing virus concentration and virus shedding in various animal models(6-10). Worldwide efforts resulted in 12 candidate vaccines in different clinical phases which use intranasal as the route of administration. (11).
Phase I of this study will evaluate the safety profile and immunogenicity of different doses of the RCP recombinant spike protein COVID-19 vaccine. The selected dose will be entered into Phase II.

2.3. Benefit/ Risk assessment

There is an ongoing global pandemic of COVID-19, and WHO has approved some vaccines. However, there is no available vaccine in Iran for public vaccination yet.
We designed a placebo-controlled randomized trial to assess RCP. There were no data available from clinical trials on using the RCP recombinant spike protein COVID-19 vaccine in humans. This vaccine's available nonclinical data and data from nonclinical studies and clinical trials with the same subunit protein vaccines supported a favorable risk/benefit profile. Although solicited AEs after vaccination were expected to be manageable using the common symptom-driven standard of care as determined by the investigators, we will recruit a small group as a sentinel for more assessing the adverse events. DSMB will review the safety information of this small group before granting permission to start the main recruitment phase of the study.

2.3.1. Risk assessment

Potential Risk of clinical significance	Summary of data/Rationale for Risk	Mitigation Strategy
**Study Intervention: RCP recombinant spike protein COVID-19 vaccine**		
Potential risk for local reactions (injection site pain, tenderness, erythema / redness, swelling / induration, itching) and systemic events (nausea and vomiting, diarrhea, headache, fatigue, and myalgia) following vaccination.	These are common adverse reactions seen with other vaccines.	The Phase I study design includes three steps (see section 4.1.). The first step will include a sentinel group to closely monitor the use of controlled vaccination and more assessment of safety. The first 0.2 ml of each vaccine concentration will first be tested on one participant in the sentinel group. Each vaccine dose will be administrated to one person, and after ensuring the vaccine safety, it will be injected into the other three participants 48 hours later.

		All participants will be observed directly and at the site of vaccination for three (Phase I) or two (Phase II) hours, and they will be followed- up in the next seven days after each vaccination dose.
Unknown AEs and laboratory abnormalities with a novel vaccine.	This study is the first in human study and there is no clinical data available for this vaccine.	Phase I and II stopping rules are in place. The Sentinel group will be observed directly for 24 hours. All other participants in Phase I and Phase II will be observed for three and two hours, respectively, to assess any immediate use.<br>There will be a 24-hour on-call physician to record and manage participants' complaints.
Study Procedures		
Participants will be required to attend healthcare facilities during the global SARS-CoV-2 pandemic.	Without appropriate social distancing and PPE, there is a potential for increased exposure to SARS-CoV-2.	All swab sampling will be done at the study site and considering the participant's crowd of clients.<br>PPE will be granted to each participant during each visit.<br>The cost of the private taxi will be paid to reduce the public vehicle use.
Venipuncture will be performed during the study.	There is the risk of bleeding, bruising, hematoma formation, and infection at the venipuncture site.	Only appropriately qualified personnel would obtain the blood draw.

2.3.2. Benefit Assessment

Benefits to individual participants may include:

- Receipt of a potentially efficacious COVID-19 vaccine during a global pandemic
- Access to COVID-19 diagnostic testing
- Contributing to research to help others in a time of the global pandemic
- Receiving a predefined financial incentive for each visit.
- All participants will be covered by insurance for any related or suspected vaccine adverse events.

2.3.3. Overall Benefit/Risk Conclusion

Considering the measures have been taken to minimize risk to volunteers participating in this study, the potential risks identified in association with the RCP vaccine are justified by the anticipated benefits afforded to healthy participants.

2.4. Protocol Amendments

Amendment on April 6, 2021.

Changing the toxicity scoring for some laboratory indices:

Considering the discrepancy between the normal values of the tests carried out in the studied laboratory (Rasoul Akram Hospital), DSMB suggested and approved a change in some laboratory indices on March 28, 2021.
The following tables show the previous content in black font and the new content in red font.

**Serum**	**Attributable to lab error/pop norms**	**Mild (Grade 1)**	**Medium (Grade 2)**	**Severe (Grade 3)**	**Potentially Life Threatening (Grade 4)**
Sodium – Hyponatremia mEq/L	136-145	132 – ~~134~~ 135	130 – 131	125 – 129	< 125
Sodium – Hypernatremia mEq/L	136-145	~~144~~ 146– ~~145~~ 147	~~146~~ 148– ~~147~~ 149	~~148~~ 150 – ~~150~~ 151	> ~~150~~ 151
Potassium – Hyperkalemia mEq/L	3.7-5.5	~~5.1~~ 5.6– ~~5.2~~ 5.7	~~5.3~~ 5.8– ~~5.4~~ 5.9	~~5.5~~ 6 – ~~5.6~~ 6.1	> ~~5.6~~ 6.1

Liver Function Tests –ALT, AST increase by factor		~~1.1~~ 1.5 – 2.5 x ULN	2.6 – 5.0 x ULN	5.1 – 10 x ULN	> 10 x ULN
Bilirubin – when accompanied by any increase in Liver Function Test increase by factor		~~1.1~~ 1.5– ~~1.25~~ 1.75x ULN	~~1.26~~ 1.76– ~~1.5~~ 2x ULN	~~1.51~~ 2.1– ~~1.75~~ 2.25x ULN	>~~1.75~~ 2.25x ULN
Bilirubin – when Liver Function Test is normal; increase by factor		~~1.1~~ 1.5– ~~1.5~~ 2x ULN	~~1.6~~ 2.1 – ~~2.0~~ 2.5x ULN	~~2.0~~ 2.6 – 3.0 x ULN	> 3.0 x ULN

**Hematology**	**Attributable to lab error/pop norms**	**Mild (Grade 1)**	**Medium (Grade 2)**	**Severe (Grade 3)**	**Potentially Life Threatening (Grade 4)**
Hemoglobin (Female) - gm/dL	11-12	~~11.0~~ 10.5 ~~12.0~~ 10.9	9.5 – ~~10.9~~ 10.4	8.0 – 9.4	< 8.0

**Urine**	**Attributable to lab error**	**Mild (Grade 1)**	**Medium (Grade 2)**	**Severe (Grade 3)**	**Potentially Life Threatening (Grade 4)**
Blood (microscopic) – red blood cells per high power field (rbc/hpf)	1-5	6 ~~0~~ - 10	11 – 50	> 50 and/or gross blood	Hospitalization or packed red blood cells (PRBC) transfusion

Changing the time of the interim analysis to decide on the start of Phase II and Phase III:
Reason for change: Accelerating the clinical trial considering the satisfactory clinical safety results of phase I (where 124 people had received the first vaccine untill updating).
Previous Content: After the start of phase II (with the selected dose), an interim analysis (same as in phase I) will be performed two weeks after the second dose of vaccine, except that it will be done when the first 250 (50%) participants complete their visit on day 35. A request for the start of Phase III will be submitted to FDO (after DSMB approval) if none of the study discontinuation criteria (listed on section 8) occur in Phase II participants and the neutralizing antibody response occurs in more than 50% of participants.
Amended content: After the start of phase II (the selected dose), the interim analysis will be performed when 30% of the participants have completed their 35-day visit. Therefore, if none of the study discontinuation criteria (listed on section 8) occur in phase II and the neutralizing antibody response occurs in more than 30% of participants, a request to start phase III will be sent to the FDA.

Changing the duration of the immediate adverse reactions evaluation in phase II:
Reason for change: Accelerate the clinical trial considering the satisfactory clinical safety results in phase I until 124 people receive the first vaccine dose.
Previous Content: After vaccination, candidates are monitored for three hours and vital signs are examined every hour.
Amended Content: After vaccination, candidates are monitored for two hours and vital signs are assessed every 40 minutes. Accordingly, study objectives and outcomes were changed.

Removing some tests from the screening and one-week post-vaccination follow-up
Reason for change: Reducing the workload of study executors by removing unnecessary tests according to the results of phase I
Previous and amended content: removed tests has been shown in following table.

	**Screening**	**Post vaccination**
Hemoglobin gm/dL	√	√
WBC cell/mm3	√	√
Lymphocytes cell/mm3	√	√
Neutrophils cell/mm3	√	√
Eosinophils - cell/mm3	√	√
Platelets cell/mm3	√	√
PT (prothrombin time)	√	-
PTT (partial thromboplastin time)	√	-

ESR	√	√
CRP	√	√
RT-PCR for SARS-CoV-2	√	-
IgM/IgG for SARS-CoV-2	√	-
HBsAg, HBcAb	√	-
HIV	√	-
HCV	√	-
Sodium, mEq/L	√	~~√~~
Potassium, mEq/L	√	~~√~~
~~Calcium, mg/dL~~	~~√~~	-
~~Magnesium, mg/dL~~	~~√~~	-
~~Phosphorous, mg/dL~~	~~√~~	-
HbA1c	√	-
BUN mg/dL	√	√
Creatinine – mg/dL	√	√
~~Albumin, g/dL~~	~~√~~	~~√~~
~~Total Protein, g/dL~~	~~√~~	~~√~~
~~Uric Acid mg/dL~~	~~√~~	~~√~~
~~LDH~~	~~√~~	~~√~~
~~Bilirubin (total)~~	~~√~~	~~√~~
Alkaline phosphatase IU/L	√	√
ALT, AST IU/L	√	√
β-hCG (on the day of vac)	√	√
U/A, Urine protein	√	√
U/A, Urine glucose	√	√
U/A, RBC	√	√

Changing inclusion criteria in phase II:
Reason for change: To facilitate the process of participant recruitment in the Phase II study.
Previous content: Overall, all the criteria are the same as in phase I, except for the age variable: Participants aged 18-70 years
Amended content: Overall, the criteria are the same as in phase I, but in this phase, people with controlled health problems can also enter the study. Meanwhile, education and the pregnancy test were changed as follows.
Education: Ability to read and write, preferably a diploma
Pregnancy test: If there is enough clinical and laboratory evidence to deny pregnancy, based on the direct decision of PI, there will not be needed to repeat the pregnancy test (β-hCG test) on the day of vaccination.

Change schedule of Phase 2 serum sampling:
Reason for change: Reducing the workload of laboratory experts and reducing costs
Previous content: Taking serum samples for assessing the level of specific IgG antibodies against components S, S1, S2, RBD, and NTD by ELISA on days 0, 21, 35, 51, 90, and 180.
Modified content: Taking serum samples for assessing the level of specific IgG antibodies against components S, S1, S2, RBD, and NTD by ELISA on days 0, 21, 35, 90, and 180.

Amendment on September 6, 2021

Considering the availability of the COVID-19 vaccine for the study population (because starting general vaccination), the code of the participants in Phase I and Phase II will be announced as follows:
Phase I candidates: After day 180, and performing clinical examination, assessing probable adverse events and taking blood samples participants will be informed of their received vaccine. Follow-up of people who have received 10, and 20 µg/200µl doses will continue for one year.
Phase I candidates: After day 180, performing a clinical examination, assessing probable adverse events, and taking blood samples, participants will be informed of their received vaccine. Follow-up of people who have received 10 and 20 µg/200µl doses will continue for one year.

Amendment on January 16, 2022

Changing the duration of follow-up in phases 1 and 2

Cause of change: The need to re-vaccinate people and receive a booster dose
Previous content:
Phase I candidates: On day 180 and after performing a clinical examination, assessing probable adverse events, and taking blood samples, participants will be informed of their received vaccine. Follow-up of people who have received 10 and 20 µg/200µl doses will continue for one year.
Phase II Candidates: At least after day 90, and performing clinical examination, assessing probable adverse events and taking blood samples participants will be informed of their received vaccine. Follow-up of vaccine groups will continue for one year.
Modified content:
Phase one candidates: will not be followed up after decoding (month 6).
Phase II volunteers: After decoding on day 90, the vaccine group will be followed-up for six months.

Assessing mucosal immunity
When the first interim analysis results were known on the selected vaccine strength to enter the phase II trial, an additional group (n=20) of open-labeled and non-randomized subjects were recruited to assess mucosal protection. They received either a ten µg intranasal vaccine or a placebo for the third dose (third step).
IgA antibody response to RBD antigen in the nasopharyngeal mucosal fluid was evaluated on days 0, 65, 120, and 150.

3. Objectives, Estimands, and Endpoints

For Phase I

Objective	Estimand	Endpoints
Primary		
To describe the safety profile of solicited adverse events of RCP in healthy adults after the first, second, and third dose of RCP recombinant spike protein COVID-19 vaccine.	In participants who received at least one dose of study intervention, the percentage of participants reported:<br>• Abnormal vital signs and anaphylactic reactions in the first 3 hours after vaccination<br>• Local adverse events within the first week after vaccination<br>• Systemic adverse events within the first week after vaccination<br>• Abnormal results of laboratory findings(at days 7, 28, and 58)	• Abnormal vital signs within the first three hours after each vaccination visit (days 0, 21 and 51) based on the intensity score (Appendix 1). Vital signs, including body temperature, respiratory rate, heartbeat rate, and systolic and diastolic blood pressure, are measured before and immediately after vaccination. Then, these measurements will be repeated every hour for at least three hours. If any unintended change in the individual's general condition, the measurements will be repeated.<br>• Anaphylaxis is defined as an immediate systemic hypersensitivity that requires simultaneous involvement of both systems. Anaphylactic reactions including erythema, pruritus, urticaria and angioedema, bronchospasm, laryngeal edema, dizziness, hypotension, nausea, shortness of breath, wheezing, arrhythmia, cyanosis, vomiting, diarrhea, abdominal pain will be evaluated up to three hours after each vaccination<br>• Local reactions (pain, tenderness, erythema / redness, swelling / induration, itching)<br>• Systemic events (fever, nausea and vomiting, diarrhea, headache, fatigue, muscle pain)<br>• Biochemistry, hematology, and urine tests
**Secondary**		
To describe the unsolicited AE elicited by RCP recombinant spike protein COVID-19 vaccine	• Number and percentage of:<br>• Severe Adverse event (SAEs) up to six months after first dose<br>• Suspected Unexpected Serious Adverse Reaction up to six month<br>• Medically Attended Adverse Events	• SAE<br>• SUSAR<br>• MAAEs
To describe the immune responses	• Geometric mean Area Under Curve (GM_{AUC}) of specific IgG antibodies.<br>• Geometric mean ratio (GMR)<br>• Geometric Mean Fold Increase (GMFI)<br>• Geometric Mean Fold Ratio (GMFR)	• Specific IgG antibodies against S<br>• Specific IgG antibodies against S1<br>• Specific IgG antibodies against S2<br>• Specific IgG antibodies against RBD<br>• Specific IgG antibodies against NTD

	• Seroconversion rate (the proportion of the individuals whose serum IgG levels have two fold or more using the ELISA method)	
	• GM $_{AUC}$ of specific IgA<br>• GMR of IgA Secretory activity levels	• Specific IgA antibodies against RBD
	• Geometric mean titers (GMTs) at each time point<br>• Geometric mean ratio (GMR)<br>• Proportion of participants achieving ≥4-fold rise from before vaccination to each subsequent time point after vaccination	• Neutralizing antibody activity
	• Counting the number of CD cells<br>• Level of Gamma interferon (IFNγ), tumor necrosis factor (TNFα), and interleukin IL-2, 4, 6, and 17	• CD3, CD4 and CD8 cells and joint calculation of CD3 and CD4 and CD3 and CD8<br>• - Gamma interferon (IFNγ), tumor necrosis factor (TNFα), and interleukin IL-2, 4, 6, and 17 assessed by ELISA

For Phase II

**Objective**	**Estimand**	**Endpoints**
Primary		
To describe safety profile of solicited adverse event of RCP in healthy adults after first, second, and third dose of RCP recombinant spike protein COVID-19 vaccine	In participants who received at least one dose of study intervention, the percentage of participants reporting:<br>• Abnormal vital signs and anaphylactic reactions in first 2 hours after vaccination<br>• Local adverse events within the first week after vaccination<br>• Systemic adverse events within the first week after vaccination	• Immediate adverse reaction (same as phase I)<br>• Local reactions (pain, tenderness, erythema / redness, swelling / induration, itching)<br>• Systemic events (fever, nausea and vomiting, diarrhea, headache, fatigue, muscle pain)
To describe the immune response	• Geometric mean Area Under Curve (GM $_{AUC}$) of specific IgG antibodies.<br>• Geometric mean ratio (GMR)<br>• Geometric Mean Fold Increase (GMFI)<br>• Geometric Mean Fold Ratio (GMFR)<br>• Seroconversion rate (the proportion of the individuals whose serum IgG levels have two fold or more using the ELISA method)	• Specific IgG antibodies against S<br>• Specific IgG antibodies against S1<br>• Specific IgG antibodies against S2<br>• Specific IgG antibodies against RBD<br>• Specific IgG antibodies against NTD
**Secondary**		
To describe the unsolicited AE<br>elicited by RCP recombinant spike protein COVID-19 vaccine	Number and percentage of:<br>• Abnormal results of laboratory findings<br>• Severe Adverse event (SAEs) up to six months after first dose<br>• Suspected Unexpected Serious Adverse Reaction up to six month<br>• Medically Attended Adverse Events	• Biochemistry, hematology, and urine tests<br>• SAE<br>• SUSAR<br>• MAAEs
	• Number and percentage of COVID-19 disease occurrence two weeks after second vaccine dose	• COVID-19 positive PCR
To describe the immune responses	• Geometric mean titers (GMTs) at each time point<br>• Geometric mean ratio (GMR)<br>• Proportion of participants achieving ≥4-fold rise from before vaccination to each subsequent time point after vaccination	• Neutralizing antibody activity
	• Counting the number of CD cells<br>• Level of Gamma interferon (IFNγ), tumor necrosis factor (TNFα), and interleukin IL-2, 4, 6, and 17	• CD3, CD4 and CD8 cells and joint calculation of CD3 and CD4 and CD3 and CD8<br>• Gamma interferon (IFNγ), tumor necrosis factor (TNFα), and interleukin IL-2, 4, 6, and 17 assessed by ELISA

4. STUDY DESIGN:

4.1. Overall Design

This is a randomized, double-blind phase I and II clinical trials in healthy adults. Phase I is a first-in-human, randomized, placebo-controlled, double-blinded study to evaluate the safety and immunogenicity of a recombinant spike protein COVID-19 vaccine (RCP) in 18-55 years old females and males. Based on medical history, physical examination, baseline clinical laboratory testing, and serology tests, subjects will be selected from healthy adults.

In phase I, participants will be recruited in 3 steps. In the first step, a small group of 13 sentinel subjects will receive either placebo or one of three strengths of vaccine at 5, 10, and 20-µg/200µl in an open-labeled and non-randomized manner. Candidates will receive low, medium, and high vaccine doses, respectively. To this end, a 5µg dose is initially injected into only one person followed by a 10µg dose after a 48-hour interval. A 10µg dose is initially injected into only one person, and there will be a 48-hour interval between this dose and the 20µg dose. When it is confirmed that

there is no problem, each vaccine dose will be injected into three other people. Therefore, 12 people will receive the vaccine and one person will receive the adjuvant. The data and safety monitoring board (DSMB) will review the safety information of this small group before granting permission to enter the main recruitment phase of the study.
In the second step, 120 individuals will be randomly assigned 1:1:1:1 ratio to receive either placebo or one of three strengths of vaccine at 5, 10, and 20-µg/200µl.
In third step, when the results of the first interim analysis on the selected vaccine strength to enter the phase II trial will be known, an additional group of open-labeled and non-randomized subjects will be recruited to assess mucosal protection and receive either 10µg intranasal vaccine or placebo for the third dose.
Phase II:
One dose will be selected based on safety and immunogenicity data generated during Phase I after an interim analysis. Phase II is a randomized, double-blind clinical trial with a parallel design. Participants in this phase will be 18-70 years of age. Five hundred participants will be assigned to either placebo or vaccine (selected dose in Phase I).

4.2. Scientific Rationale for Study Design

Additional surveillance for COVID-19 will be conducted as part of the study, given the potential risk of disease enhancement. If a participant experiences symptoms, as detailed in appendix 3 a COVID-19 illness and subsequent convalescent visit will occur. As part of these visits, samples (nasal [midturbinate] swab and blood) will be taken for antigen and antibody assessment as well as recording of COVID-19–related clinical and laboratory information (including local diagnosis).
Additional visits will be performed for AEs which need to be followed up. In this visits, physical examination will be done by a general practitioner or related specialist. Blood sampling or additional diagnostic tests will be done if required.
Human reproductive safety data are not available for RCP vaccine, but there is no suspicion of human teratogenicity based on the intended mechanism of action of the compound. Therefore, the use of a highly effective method of contraception is required. Furthermore, breastfeeding women will be excluded from the study.

5. Study population

This study can fulfill its objectives only if appropriate participants are enrolled. The following eligibility criteria are designed to select participants for whom participation in the study is appropriate. All relevant medical and nonmedical conditions should be considered when deciding whether a particular participant is suitable for this protocol.

5.1. Inclusion Criteria

5.1.1. Inclusion Criteria for Phase I

1. Having Iranian citizenship and residence at a reasonable distance (40-50 km) from the study site
2. Having at least diploma degree
3. Aged 18-55 years
4. Body mass index (BMI) between 17-35 kg/ m^2
5. Being generally healthy based on clinical and laboratory examinations
6. Having sublingual temperatures ≤ 37.2 °C based on mercury or digital thermometer
7. Heart rate between 60 and 100 beats per minute
8. Negative IgG and IgM antibody titers against Covid-19 antigen
9. Negative RT-PCR test to detect Covid-19
10. Negative blood HIV IgG ELISA test
11. Signing the informed consent form
12. Accepting commitments to reduce the risk of Covid-19 according to Table 1
13. Unwillingness to have children and using safe contraceptives until three months after the vaccination period (including men and women)
14. Being not pregnant based on the participant's statement from the first day of the last menstrual period
15. Negative β-hCG test on the day of screening and vaccination
16. Using at least one safe contraceptive method (condoms, oral contraceptive pills, intrauterine contraceptive device (IUD), Norplant capsule) for women of childbearing, i.e. 18 to 49 years
17. Tendency to continue using at least one safe contraceptive methods (condoms, oral contraceptive pills, IUD, Norplant capsule) for women of childbearing age, i.e.18-49 years until 3 months after the end of the vaccination
18. Psychiatric confirmation of having the capacity and mental health to decide to participate in the study

Table S1. List of participants' commitments to reduce the risk of exposure to Covid-19 virus within the first month after vaccination

Number	Description of commitment within the first month after vaccination
1	I do not leave the house except to meet essential needs such as food and medicine without an alternative.
2	If I have to leave the house, I will use a mask.
3	If I have to leave the house, I do not use public transportation at all and use a private vehicle or exclusive taxi service.
4	I avoid attending family parties except with very close relatives with whom I am in daily contact.
5	I avoid attending gatherings.
6	I use leave or work remotely as much as possible.
7	I wash my hands regularly with soap and water.
8	In cases where washing with soap and water is not possible, I disinfect my hands with 70% alcohol.
9	I will notify the study researchers if a family member / close colleague becomes ill.

5.1.2. Inclusion Criteria in Phase II

Except following items, criteria in phase II are generally the same as in phase I
- People with controlled health problems can also enter this phase
- Age 18 to 70 years
- Ability to read and write, preferably high school diploma
- If the principal investigator rules out pregnancy based on clinical or laboratory findings, there will be no need to repeat the β-hCG test on the day of vaccination.

5.2. Exclusion Criteria

5.2.1. Exclusion Criteria in Phase I

1. Having an ongoing, symptomatic acute or chronic illness requiring medication or surgery
2. Working in an occupation with a high risk of exposure to COVID-19 including medical staff, occupations with close contact with the client;
3. Breastfeeding
4. Having a history of any vaccination during the 30 days prior to screening
5. Having a history of receiving blood or any blood product or immunoglobulin within 3 months prior to screening
6. Having immunosuppressive diseases (suspected and confirmed)
7. Having a history of long-term use of immunosuppressive drugs or systemic corticosteroids in the last 4 months leading up to screening day
8. Having a history of long-term use (more than 14 consecutive days) of systemic corticosteroids (10 mg≥ daily prednisolone) during the last 4 months leading up to screening day (excluding topical steroids)
9. Having a history of allergic diseases such as angioedema or anaphylactic reactions
10. Having a history of any allergic reaction to the drug or vaccine (including clinical symptoms of itching, urticaria, excessive redness at the injection site)
11. Having a history of autoimmune diseases (other than controlled autoimmune thyroid disease, and stable and controlled celiac disease, mild psoriasis, and vitiligo that do not require corticosteroid or immunosuppressive therapy)
12. Having a history of chemotherapy in the last 5 years
13. Having a history of cancer in the last 5 years
14. Having a history of serious psychiatric illnesses
15. Having a history of blood disorders (dyscrasia, coagulation, platelet deficiency or disorder, deficiency of blood factors)
16. Having chronic obstructive pulmonary diseases such as asthma and COPD, diagnosed and treated by a specialist.
17. Having Ischemic cardiovascular disease treated by a physician or having a history of cardiac interventions
18. Having high blood pressure even treated by a physician.
19. Having diabetes even treated by a physician.
20. Having a history of chronic neurological diseases (including seizures and epilepsy)
21. Having any history of substance abuse or addiction during the last 2 years
22. Having any grade I> problem in the results of hematology or biochemical tests performed upon the screening day according to Tables 9-12 of Appendix 1, toxicity scoring, page 93.
23. Having a history of a confirmed COVID-19
24. Acute febrile illness at the time of vaccination

25. Having a history of acetaminophen allergy
26. Acute or chronic hepatitis B and C
27. Receiving tuberculosis prophylaxis
28. Having a history of syncope with blood transfusion or observation
29. Splenectomy for any reason
30. Any close contact with a person with a confirmed Covid-19 within two weeks before the first vaccine dose (see Appendix 3.)
31. Previous history of diseases such as SARS, MERS
32. Participating in any clinical (research) study other than the present study

5.2.2. Exclusion Criteria in Phase II

All criteria are the same as phase I, except for absence of diabetes and high blood pressure:
Having high blood pressure that is treated by a physician and is not controlled (systolic blood pressure above 140mm Hg and diastolic blood pressure above 90mm Hg).
Having diabetes that is treated by a physician and is not controlled (HBA1c> 7%) or treated using insulin.

6. Study intervention

Study intervention is defined as any investigational intervention(s), marketed product(s), placebo, medical device(s), or study procedure(s) intended to be administered to a study participant according to the study protocol.
The Phase I study will evaluate the safety and immunogenicity of various different doses RCP to find the best dose to enter the Phase II.
In Phase I, we considered four following groups:
Intervention group 1: Vaccine at 5 µg/200µl dose; Participants in this group will receive two doses (IM) of RCP vaccine 21 days apart followed by a 10 µg/200µl nasal spray 51 days after the first dose (day 0)
Intervention group 2: Vaccine at 10 µg/200µl dose; Participants in this group will receive two doses (IM) of RCP vaccine 21 days apart followed by a 10 µg/200µl nasal spray 51 days after the first dose (day 0)
Intervention group 3: Vaccine at 20 µg/200µl dose; Participants in this group will receive two doses (IM) of RCP vaccine 21 days apart followed by a 10 µg/200µl nasal spray 51 days after the first dose (day 0)
Control group, Adjuvant:
Control group: Adjuvant; Participants in this group will receive two doses (IM) of Adjuvant by 50% v/v concentration produced in RAZI institute 21 days apart followed by another dose in the form of nasal spray at day 51 (counted from day 0)
The vaccine dose selected for Phase 2 will be comprised by adjuvant group.

6.1. Storage, handling and preparation

Vaccine / IMP: The relevant vaccine / IMP is stored at 4 to 8 ° C. These are single-dose vaccine / IMP vials (vaccine and adjutant).
Unopened vials can be stored in the centers' refrigerator for up to six months. The refrigerator temperature will be controlled using a data logger and should be kept in the range of 4-8 °C. Attempts are made to transport vaccines to the clinical center in small quantities for a maximum of one month.
Inhalation spray: Inhalation spray is stored at 4 to 8 °C. This is single-dose spray. These vials will be managed like injectable vials.

6.1.1. Administration route

The vaccine / adjuvant is injected into the upper part of the deltoid area (See Appendix 4).
The vaccinator should wash his/her hands with soap and water before injection.
The injection site should be disinfected with an alcohol pad in a circular motion from the center to the outside.
The vaccine site should be washed with soap and water if it is dirty.
The vaccine must be injected after the alcohol dries.
An insulin syringe is used for injection.
The injection dose is equal to 0.2 CC.
It is advisable to administer both intramuscular doses in one arm.
The vaccine / adjuvant intra-nasal spraying will be carried out with one spray puff in the nasal cavity.

6.1.2. Adherence monitoring strategies

The research team injected the vaccine / IMP in the studied clinical unit should be intended and recorded in the CRF. The unit staff will confirm whether the participant has received the full vaccine / IMP.
The site (left or right arm), time and date of all vaccines are recorded in CRF by the research team member. If a participant does not receive all or part of the vaccine, the reason will be recorded in the CRF and software.
If the candidate does not apply for the vaccine / IMP or blood sample, telephone follow-up is carried out first. If necessary, an in-person visit will be done at the participant's site.

6.2. Measures to Minimize Bias: Randomization and Blinding

6.2.1. Allocation to Study Intervention

We used block randomization with variable block sizes to allocate the study participants into the three groups receiving three different strengths of the vaccine and a placebo (adjuvant only) group. Rand function in excel software was utilized to generate the random sequence within each block by the study epidemiologist (MSD). Four-digit unique codes were assigned to each participant to conceal the allocation. The randomization sequence was embedded in the software specifically developed to manage the data collection alongside paper CRFs in this study. All three vaccine strengths and the placebo had the same volume and color in single-dose preparations. An independent team carried out the labeling.

6.2.2. Blinding of Site Personnel and Sponsor

In this double-blinded study, all study and site personnel, including the investigators, laboratory staff, and participants, will be blinded to study intervention assignments.
The individuals who evaluate participant safety will be blinded. All members of the sponsor will be blinded to study intervention allocation.
All the vaccine strengths in both Phases along with the placebo have the same volume and color in single-dose preparations. An independent team will carry out vial labeling, and each vial will be delivered to the study site based on a randomized code.
Blood specimens collected to assess immunogenicity were coded to blind the immunology lab operators during this research. We used a code pair for this purpose. One of the codes was covered with a scratch label and used to label the blood collection tubes. The other was put in the appropriate place in the paper CRF. The study epidemiologist kept the key to the pairs. Accordingly, all the participants and the research team (including lab staff and executive team) were unaware of the allocated intervention and intermediary immunogenicity outcomes.

6.2.3. Un-blinding

Un-blinding can be carried out in the following cases:
1. Incidence of SAE
2. Obvious biochemical or hematological changes in tests (Grade 3 toxicity or higher)
3. Pregnancy
4. Severe disorder in vital signs, local or systemic symptoms (Grade 3 toxicity or higher)

As soon as any of the above cases occurs, the center physician will explain the reason(s) for the need to un-blinding and perform related treatments after coordination with the principal investigator. In such cases, the reasons for the breaking blind are recorded in the CRF.

6.3. Study Intervention Compliance

When participants are dosed at the site, they will receive study intervention directly from the investigator or designee, under medical supervision. The date and time of each dose administered in the clinic will be recorded in the software and CRF.

7. Withdrawal criteria

7.1. Withdrawal criteria in Phase I

People can withdraw from the study at any time and for any reason. If the candidate refuses to continue receiving the vaccine /IMP, he or she will be advised to stay in the study for safety follow-up. Clinical information and

complications follow-up will continue for these individuals, until the end of the study. Also, study withdrawal does not mean that participants' safety consequences will not be followed up until the end of the study.
The vaccination process is stopped in the event of following cases:
- Non-adherence to interventions and diagnostic processes
- No attendance for a second vaccine within 7 days of the scheduled date despite three telephone follow-ups at 24-hour intervals
- Impossibility of performing laboratory safety assessment: two times with a maximum 7-day interval from the specified date despite three telephone follow-ups at 24-hour intervals
- Impossibility of face-to-face clinical safety assessment: more than two consecutive or three non-consecutive times since the appointed date despite three telephone follow-ups at 24 –hour interval
- No attendance for immunogenicity assessment: Two consecutive or three non-consecutive times since the appointed date despite three telephone follow-ups at 24-hour interval
- Occurrence of SAE for which DSMB / regulator recommends vaccine discontinuation
- The occurrence of obvious biochemical or hematological changes in tests, according to the researcher's diagnosis, the vaccination continuation may endanger the health
- Confirmed COVID-19 infection
- Any disease requires major drug interventions such as intravenous antibiotics or broad-spectrum oral antibiotics, blood or blood products, immunosuppressive drugs, or systemic corticosteroids at the discretion of the principal investigator.
In all the above cases, withdrawal reasons are recorded in the relevant CRF.

7.2. Withdrawal criteria in Phase II

All criteria are the same as phase I

8. Pause Rules

The vaccination process will be stopped, and a DSMB session will be held if any of the following cases is observed. A decision will be made regarding the continuation of the vaccination process based on the opinion of the members of this board,
- Occurrence of any serious vaccine-related adverse event (SAE) following vaccine / IMP injection
- Incidence of serious local toxicity, vital or systemic symptoms (grade 3 according to Tables 9, 10, and 11) in more than 30% of each intervention group one month after each vaccine / IMP dose
- Occurrence of severe laboratory toxicity (grade 3 according to Tables 12 and 13) in more than 30% of each intervention group one month after each vaccine / IMP dose. Tests include creatinine, AST, ALT, bilirubin, hemoglobin, complete white blood cell and platelet count, and urine tests.
Regardless of the above rules, the sponsor can request a DSMB meeting at any time if he or she feels concerned about continuing the trial.

9. STUDY PROCEDURES

9.1. Study conduct site

The study setting will be located in the southern part of Rasoul Akram Hospital and on the first floor of its office building. Entry and exit to this ward will be the at the south door of the hospital located at the corner of Shahid Maziar Mansouri Street and Niayesh Street, which is only for personnel entry and not patients. The inpatient and emergency wards of the hospital are located in the northern part and patient entry and exit are allowed from the north and northeast doors. This place will be equipped with the necessary equipment to conduct the present trial. Also, the necessary training will be provided to fellow researchers during the study period.

9.2. Recruitment Strategy

After the protocol is approved by the Drug Administration, the National Ethics Committee and the DSMB, participants will be invited to study through a specific website. On this website, the necessary study information will be uploaded for the participants and they can complete the initial screening questionnaire online if they wish. Their information is used to determine their eligibility for the study. Candidates who are most likely to be eligible will be invited to the study.

9.3. Screening

Candidates will be screened before participating in the study, at two levels of Online (Digital) and face-to-face (On-site) screening to meet the inclusion and exclusion criteria

9.3.1. Digital screening

Based on the announced call and the plan set by the research team, the selected candidates will attend the study conducting site. At the end of the screening process on the site, if the candidate is eligible, he/she can be invited to study for up to a month (preferably a week).
Volunteers who accept the invitation and come to the center will enter the next stages of the study by trained staff. First, the details of each candidate are written in the registration form. Afterward, necessary explanations are given to candidates regarding the vaccine, study stages, tests and frequency of blood sampling and interventions (vaccine and adjutant), and the benefits or harms of participating in the study. Women will also be asked about their pregnancy and breastfeeding. The interview is continued in the absence of these two issues. Then, the informed consent form is provided to the candidates and the relevant expert gives them the opportunity to study, make informed decisions and provide a complete description of the subject matter and also helps them understand its various clauses. When the candidates fully study the form and the researcher ensures their complete agreement and deep satisfaction, they will announce their readiness to participate in the study. Participants are then asked to sign the informed consent in two copies and place a fingerprint below it.
After receiving the informed consent, the general practitioner first carefully examines each candidate for the inclusion and exclusion criteria. If any of the candidates did not get permission to enter the study, the reason would be written in the relevant screening form. A full history of the candidate's health status is taken, and a full examination of all organs (neck, ears, eyes, nose, mouth, lymph nodes, heart and arteries, lungs, abdomen, limbs, nervous system) and registration of vital signs (blood pressure, temperature, respiration rate, and heart rate) are done and recorded to complete the file. Then, in case of clinical confirmation, to check the normal range of laboratory indicators, some tests are requested and the candidate is referred to the laboratory for testing to confirm the normal range of laboratory indicators and receive the clinical approval.
Tests will be requested for candidates according to the first column of Table 2. The laboratory test results should be within the normal range as determined by the project staff. If any of the test results are abnormal, the test is repeated once and if it is within the normal range, the new number is considered a criterion for entering the study. Otherwise, that participant will not enter the study. If there were slight symptoms of acute and transient illness upon the initial examination, the examination and testing are postponed and the visit is performed only once on another day. Each candidate will participate in a screening phase for a maximum of two visits. Finally, a medical file will be prepared for the eligible candidates, and they will be invited for randomization and vaccine / IMP receiving.
Participants are generally allowed to continue with the medications they have already taken. However, this rule should not be a problem given the entry and non-entry conditions for the study. In Phase I of the study, healthy individuals are selected for inclusion in the study, so authorized therapies will not be a problem. In Phase II, some people with controlled blood pressure or diabetes are allowed to enter the study and they can continue their medications. In general, participants are allowed to continue their previous medications, and it does not seem to be a problem to follow this rule, given the inclusion and exclusion criteria. Moreover, some people with controlled blood pressure or diabetes are allowed to enter phase II of the study and can continue all the medications they have already taken.

9.4. Lab Assessments

Diagnostic and safety tests will be performed, based on items in Table 2, in the medical diagnostic laboratory of Rasoul Hospital. Necessary contracts have been signed between the hospital management and the study sponsor in this regard. Sampling is performed A hospital information management (HIS)-connected computer station will be installed in the study setting. A labeling system will be used to assign a label containing the first and last names and national ID card numbers of the candidates. This national ID card number will be printed by the related printer in the study setting. Participants will not be charged for any of the tests. Safety test samples will be stored in accordance with the guidelines for at least one month.

Table S2. Complete list of biochemistry, hematology and urine tests in different stages of the study

	**Phase I**		**Phase II**	
	**Screening**	**Post vaccination**	**Screening**	**Post vaccination**
Hemoglobin gm/dL	√	√	√	√
WBC cell/mm3	√	√	√	√
Lymphocytes cell/mm3	√	√	√	√
Neutrophils cell/mm3	√	√	√	√
Eosinophils - cell/mm3	√	√	√	√
Platelets cell/mm3	√	√	√	√
PT (prothrombin time)	√	-	√	-

PTT (partial thromboplastin time)	√	-	√	-
ESR	√	√	√	√
CRP	√	√	√	√
RT-PCR for SARS-CoV-2	√	-	√	-
IgM/IgG for SARS-CoV-2	√	-	√	-
HBsAg, HBcAb	√	-	√	-
HIV	√	-	√	-
HCV	√	-	√	-
Sodium, mEq/L	√	√	√	-
Potassium, mEq/L	√	√	√	-
Calcium, mg/dL	√	-		
Magnesium, mg/dL	√	-		
Phosphorous, mg/dL	√	-		
HbA1c	√	-	√	-
BUN mg/dL	√	√	√	√
Creatinine – mg/dL	√	√	√	√
Albumin, g/dL	√	√		
Total Protein, g/dL	√	√		
Uric Acid mg/dL	√	√		
LDH	√	√		
Bilirubin (total)	√	√		
Alkaline phosphatase IU/L	√	√	√	√
ALT, AST IU/L	√	√	√	√
β-hCG (on the day of vac)	√	√	√	√
U/A, Urine protein	√	√	√	√
U/A, Urine glucose	√	√	√	√
U/A, RBC	√	√	√	√

Immunogenicity assessment for coded and blinded samples will be performed in the Immunology Department of the Razi Vaccine and Serum Research Institute. Serological samples will be stored in the central refrigerator of this Institute for six months.

9.5. Sentinel participants

To ensure vaccine safety, a total of 13 sentinel participants will enter the study without blinding and with a special plan. Each vaccine concentration will first be tested on one participant. After ensuring the vaccine's safety, it will be tested on the other three participants 48 hours later. As the three participants enter the study, a higher concentration will be tested on one candidate. Complications monitoring will be performed daily for up to 7 days according to the FDA reporting system. In order for each vaccine concentration to enter phase I of the study, which is double-blind and randomized, at least 7 days must have elapsed since the vaccination of the three vaccine recipients in the previous stage. They also should not show any serious complications (severity grade 3 and above).
In all of these people, vital signs are monitored and recorded before the injection. A negative β-hCG test should be available for women on the same day. Vital signs will be measured and recorded hourly for up to three hours after vaccination. Also, to ensure adverse complications and timely response to probable complications, all sentinel participants will be under hospital care for the first 24 hours.

9.6. Participant visit plans

9.6.1. Participant visit plan in Phase I

In visit, activities are defined, the details of which will be presented below (Figure 1). At the end of each vaccination session, candidates will receive the necessary training on how to record possible vaccine / IMP- related complications a "diary card ", use a mercury thermometer and a ruler. The phone number of the on-call physician is also given to the candidates. Screening is not shown in this chart.

Figure S1. Flowchart (process) of phase I

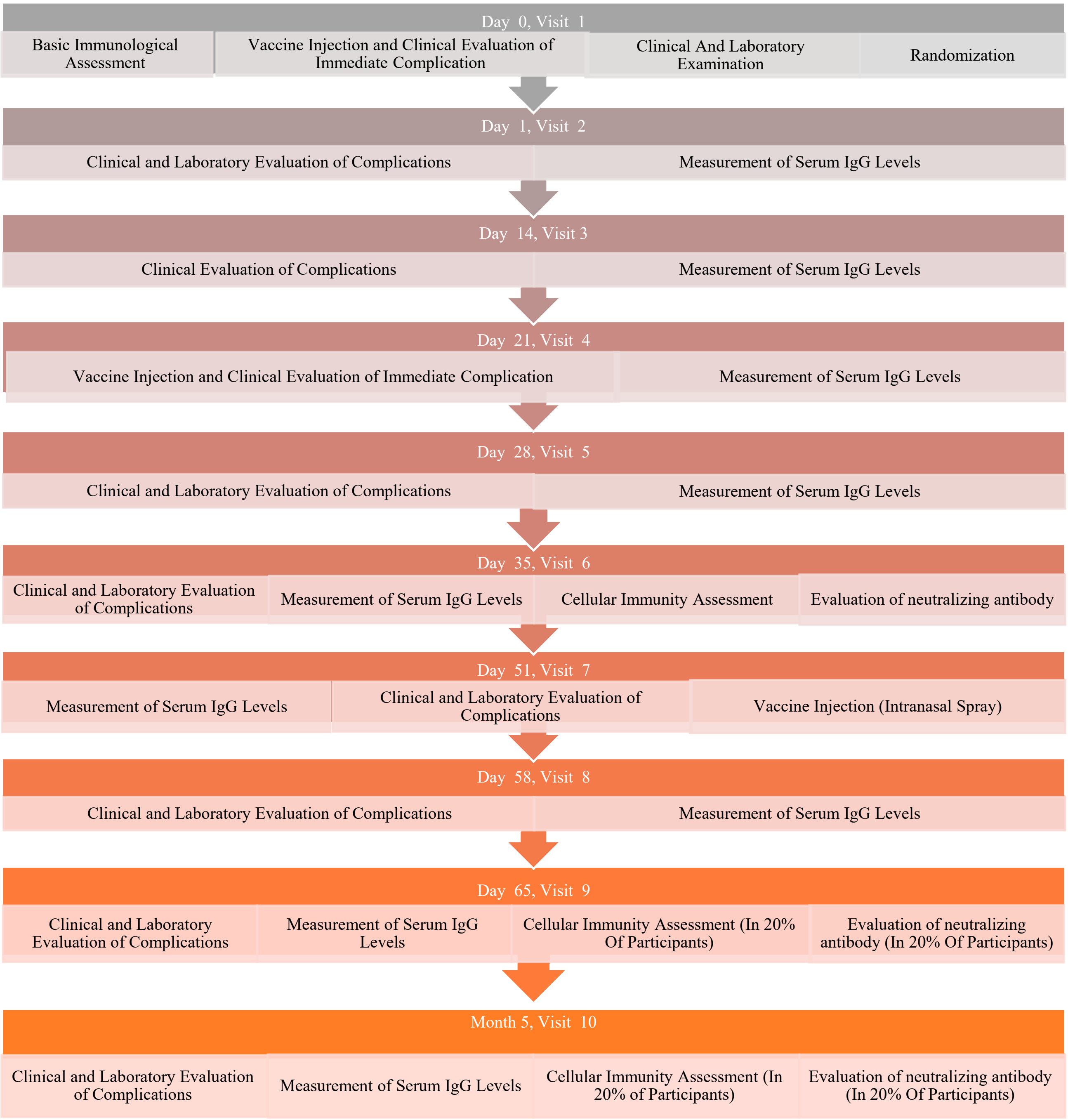
Day 0, Visit 1
Basic Immunological Assessment
Vaccine Injection and Clinical Evaluation of Immediate Complication
Clinical And Laboratory Examination
Randomization
Day 1, Visit 2
Clinical and Laboratory Evaluation of Complications
Measurement of Serum IgG Levels
Day 14, Visit 3
Clinical Evaluation of Complications
Measurement of Serum IgG Levels
Day 21, Visit 4
Vaccine Injection and Clinical Evaluation of Immediate Complication
Measurement of Serum IgG Levels
Day 28, Visit 5
Clinical and Laboratory Evaluation of Complications
Measurement of Serum IgG Levels
Day 35, Visit 6
Clinical and Laboratory Evaluation of Complications
Measurement of Serum IgG Levels
Cellular Immunity Assessment
Evaluation of neutralizing antibody
Day 51, Visit 7
Measurement of Serum IgG Levels
Clinical and Laboratory Evaluation of Complications
Vaccine Injection (Intranasal Spray)
Day 58, Visit 8
Clinical and Laboratory Evaluation of Complications
Measurement of Serum IgG Levels
Day 65, Visit 9
Clinical and Laboratory Evaluation of Complications
Measurement of Serum IgG Levels
Cellular Immunity Assessment (In 20% Of Participants)
Evaluation of neutralizing antibody (In 20% Of Participants)
Month 5, Visit 10
Clinical and Laboratory Evaluation of Complications
Measurement of Serum IgG Levels
Cellular Immunity Assessment (In 20% of Participants)
Evaluation of neutralizing antibody (In 20% Of Participants)

Phase I, day 7, screening visit

The list of activities during this visit is as follows:

- Obtaining informed consent
- Filling out the inclusion and exclusion criteria questionnaire
- Physical examination
- Blood samples for screening tests
- Control of inclusion and exclusion criteria after preparing the results of tests and examinations

Phase I, day 0, visit 1

The list of activities during this visit is as follows:

- Controlling β-hCG in women
- Taking blood samples to measure serum specific IgG antibodies levels
- Receiving dedicated vaccine / IMP *
- Examining vital signs before vaccine / IMP injection and every hour up to three post-vaccination
- Examining local and systemic symptoms three hours after vaccination
- Assessing any possible side effects
- Physical examination
- Delivery of diary card
- Delivery of package containing mercury thermometer and ruler (to record daily complications)
- Phone follow-up of complications for 6 days after the first visit

* In case of any acute febrile respiratory disease, vaccination can be delayed for up to 3 days according to the doctor's discretion.

Phase I, day 7, visit 2

The list of activities during this visit is as follows:

- Examining vital signs
- Examining local and systemic symptoms
- Assessing any possible side effects
- Physical examination
- Taking blood samples to measure serum specific IgG antibodies levels
- Blood and urine sampling for laboratory evaluation according to the second column of Table 2

Phase I, day 14, visit 3

The list of activities during this visit is as follows:

- Examining vital signs
- Examining local and systemic symptoms
- Examining any possible complications
- Physical examination
- Taking blood samples to measure serum specific IgG antibodies levels

Phase I, day 21, visit 4

The list of activities during this visit is as follows:

- Controlling β-hCG in women
- Taking blood samples to measure serum specific IgG antibodies levels
- Receiving dedicated vaccine / IMP *
- Examining vital signs before vaccine / IMP injection up to three hours every hour after vaccination
- Examining local and systemic symptoms three hours after vaccination
- Assessing any possible side effects
- Physical examination
- Delivery of diary card
- Telephone follow up of complications for 7 days after this visit

* In case of any acute febrile respiratory disease, vaccination can be delayed for up to 3 days according to the doctor's discretion.

Phase I, day 28, visit 5

The list of actions of this visit is as follows:

- Evaluate vital signs
- Evaluation of local and systemic symptoms
- Assess any possible complications
- Physical examination
- Blood samples to measure serum specific IgG antibodies levels
- Blood and urine sampling for laboratory evaluation according to the second column of Table 2

Phase I, day 35, visit 6
The list of activities during this visit is as follows:
• Examining vital signs
• Examining local and systemic symptoms
• Examining any possible complications
•Physical examination
• Taking blood samples to measure serum specific IgG antibodies levels
• Taking Blood samples to measure neutralizing antibody activity and cell-mediated immunity

Phase I, day 51, visit 7
The list of activities in this visit is as follows:
• Controlling β-hCG in women
• Receiving a dedicated vaccine / IMP (intranasal) *
• Examining vital signs before vaccination every hour three hours after vaccination
• Examining local and systemic symptoms three hour after vaccination
• Examining any possible complications
•Physical examination
• Delivery of the diary card
• Telephone follow-up for 7 days after this visit
* In case of any acute febrile respiratory disease, vaccination can be delayed for up to 3 days according to the doctor's discretion.

Phase I, day 58, visit 8
The list of activities in this visit is as follows:
• Examining vital signs
• Examining local and systemic symptoms
• Examining any possible complications
•Physical examination
• Taking blood samples to measure serum specific IgG antibodies levels
• Blood and urine sampling for laboratory evaluation according to the second column of Table 2

Phase I, day 65, visit 9
The list of activities in this visit is as follows:
• Examining vital signs
• Examining local and systemic symptoms
• Examining any possible complications
• Physical examination
•Taking blood samples to measure serum specific IgG antibodies levels
• Taking blood samples to measure neutralizing antibody activity and cell-mediated immunity

Phase I, month 5, visit 10
The list of activities in this visit is as follows:
• Examining vital signs
• Examining any possible complications
•Physical examination
• Taking blood samples to measure serum specific IgG antibodies levels
• Taking blood samples to measure neutralizing antibody activity and cell-mediated immunity

9.6.2. Participant visit plans in Phase II

In each visit, activities are defined, the details of which will be presented below (Figure 2). At the end of each vaccination session, candidates will receive the necessary training on how to record possible vaccine / IMP- related complications a "dairy card", use a mercury thermometer and a ruler. The phone number of the on-call physician is also given to the candidates. Screening is not shown in this chart.

Figure S2 Flowchart (process) of phase II

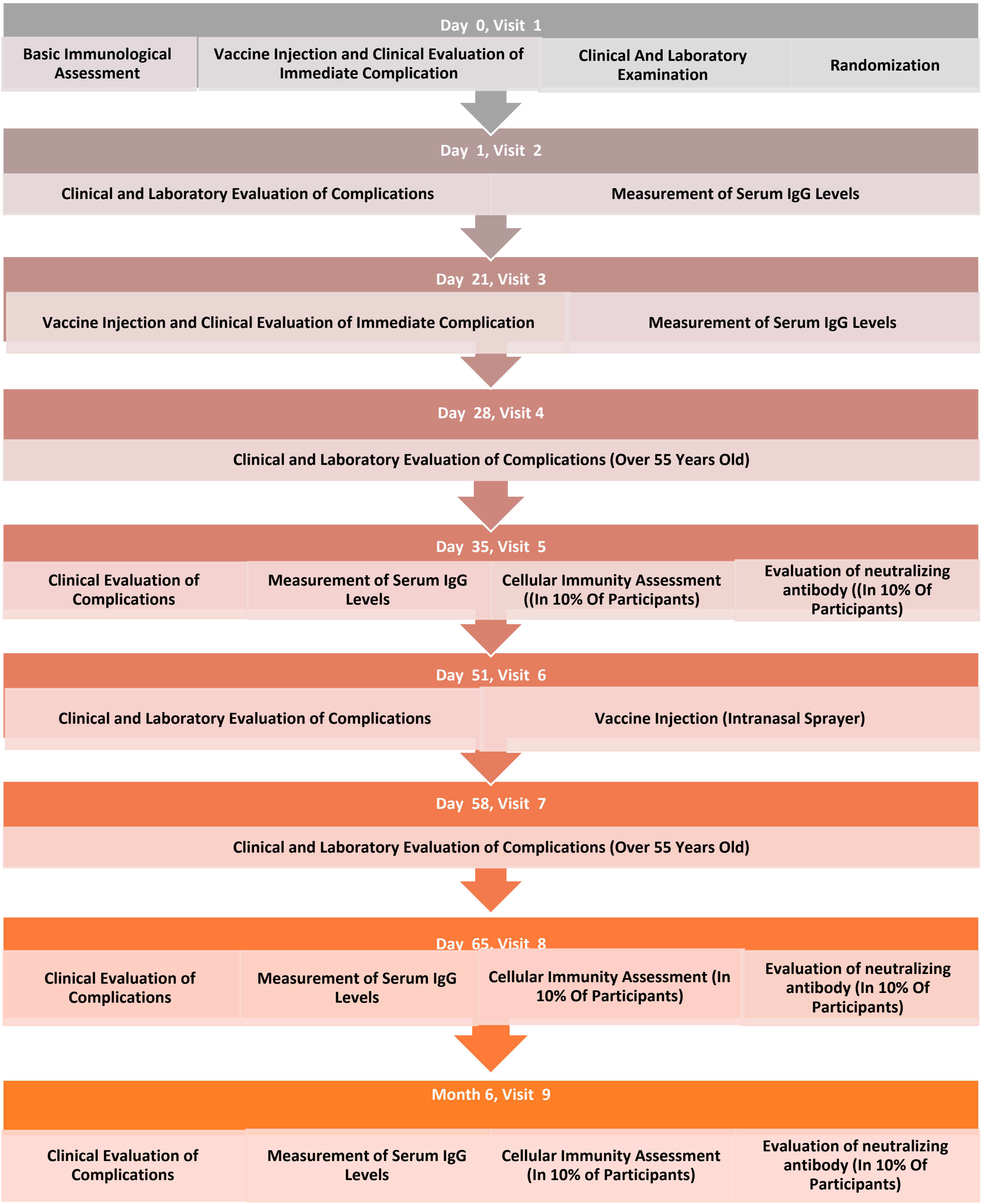
Day 0, Visit 1
Basic Immunological Assessment
Vaccine Injection and Clinical Evaluation of Immediate Complication
Clinical And Laboratory Examination
Randomization
Day 1, Visit 2
Clinical and Laboratory Evaluation of Complications
Measurement of Serum IgG Levels
Day 21, Visit 3
Vaccine Injection and Clinical Evaluation of Immediate Complication
Measurement of Serum IgG Levels
Day 28, Visit 4
Clinical and Laboratory Evaluation of Complications (Over 55 Years Old)
Day 35, Visit 5
Clinical Evaluation of Complications
Measurement of Serum IgG Levels
Cellular Immunity Assessment ((In 10% Of Participants)
Evaluation of neutralizing antibody ((In 10% Of Participants)
Day 51, Visit 6
Clinical and Laboratory Evaluation of Complications
Vaccine Injection (Intranasal Sprayer)
Day 58, Visit 7
Clinical and Laboratory Evaluation of Complications (Over 55 Years Old)
Day 65, Visit 8
Clinical Evaluation of Complications
Measurement of Serum IgG Levels
Cellular Immunity Assessment (In 10% Of Participants)
Evaluation of neutralizing antibody (In 10% Of Participants)
Month 6, Visit 9
Clinical Evaluation of Complications
Measurement of Serum IgG Levels
Cellular Immunity Assessment (In 10% of Participants)
Evaluation of neutralizing antibody (In 10% Of Participants)

Phase II, day 7, screening visit

The list of activities during this visit is as follows:
• Obtaining consent
• Filling out the inclusion and exclusion criteria questionnaire
•Physical examination
• Blood samples for screening tests
• Control of inclusion and exclusion criteria after preparing the results of tests and examinations

Phase II, day 0, visit 1

The list of activities during this visit is as follows:
• Controlling β-hCG in women
• Controlling inclusion and exclusion criteria
• Taking blood samples to measure serum specific IgG antibodies levels
•Receiving dedicated vaccine / IMP *
•Examining vital signs before vaccine / IMP injection and every forty minutes up to two hours after vaccination
•Examining local and systemic symptoms two hours after vaccination
• Assessing any possible complications
•Physical examination
• Delivery of the diary card
• Delivery of package containing mercury thermometer and ruler (to record daily complications)
• Telephone follow-up of complications for 7 days after the first visit

* In case of any acute febrile respiratory disease, according to the doctor's discretion, vaccination can be delayed for up to 3 days.

Phase II, day 7, visit 2

The list of activities during this visit is as follows:
• Examining vital signs
• Examining local and systemic symptoms
• Assessing any possible complications
•Physical examination
• Blood and urine sampling for laboratory evaluation according to the second column of Table 3

Phase II, day 21, visit 3

The list of activities during this visit is as follows:
• Controlling β-hCG in women
• Taking blood samples to measure serum specific IgG antibodies levels
• Receiving dedicated vaccine / IMP *
• Examining vital signs before vaccine / IMP injection up to forty minutes up to two hours after vaccination
•Examining local and systemic symptoms two hours after vaccination
• Assessing any possible complications
•Physical examination
• Delivery of the diary card
• Telephone follow up of complications for 7 days after this visit

* In case of any acute febrile respiratory disease, vaccination can be delayed for up to 3 days according to the doctor's discretion.

Phase II, day 28, visit 4

The list of actions of this visit is as follows:
• Evaluate vital signs
• Evaluation of local and systemic symptoms
• Assessing any possible complications
•Physical examination
• Blood and urine sampling for laboratory evaluation according to the second column of Table 3 (people older than 55 years)

Phase II, day 35, visit 5

The list of activities during this visit is as follows:
• Examining vital signs
• Examining local and systemic symptoms
• Examining any possible complications
•Physical examination
• Taking blood samples to measure serum specific IgG antibodies levels
• Taking blood samples to measure neutralizing antibody activity and cell-mediated immunity

Phase II, day 51, visit 6
The list of activities of this visit is as follows:
• Controlling β-hCG in women
• Receiving a dedicated vaccine / IMP (intranasal) *
• Examining vital signs before vaccination every forty minutes up to two hours after vaccination
• Examining local and systemic symptoms two hour after vaccination
• Examining any possible complications
•Physical examination
• Delivery of the diary card
• Telephone follow-up of complications for 7 days after this visit
* In case of any acute febrile respiratory disease, vaccination can be delayed for up to 3 days according to the doctor's discretion.

Phase II, day 58, visit 7
The list of activities in this visit is as follows:
• Examining vital signs
• Examining local and systemic symptoms
• Examining any possible complications
•Physical examination
• Blood and urine sampling for laboratory evaluation according to the second column of Table 3 (People older than 55 years of age)

Phase II, month 3, visit 8
The list of activities in this visit is as follows:
• Examining vital signs
• Examining local and systemic symptoms
• Examining any possible complications
• Physical examination
•Taking blood samples to measure serum specific IgG antibodies levels
• Taking blood samples to measure neutralizing antibody activity and cell-mediated immunity

Phase II, month 6, visit 9
The list of activities in this visit is as follows:
• Examining vital signs
• Examining any possible side effects
•Physical examination
• Taking blood samples to measure serum specific IgG antibodies levels
• Taking blood samples to measure neutralizing antibody activity and cell-mediated immunity

10. Data collection, management, and analysis

10.1. Data collection methods

Data collection will be carried out using printed CRFs designed in the form of a paper booklet. Each patient will also have a diary card. This booklet will consist of two parts, i.e. screening and assigned participants. Also, all data obtained from the candidates according to CRF format were recorded electronically in a local newly designed software.

10.2. CRF booklet for screening

This section of the booklet will include the screening forms. Suppose individuals are eligible to enter the study and accept the invitation of the research team to participate in the study. In that case, they will enter the randomization phase and undergo the intervention. This separate section will help prevent paper wastage for candidates who are not considered eligible in the screening stage.

10.3. CRF booklet for assigned participants

This section includes data collection forms for the eligible participants. Obviously, after randomization, data must be collected and entered in these forms. Data collected and recorded in the screening section of the booklet up to the point of random allocation should be appropriately added to this section of the booklet.

10.4. Diary card

This card includes patient details including name and surname, and a unique participant code. Participants are asked to record their daily temperature around 12:00 noon. Also, the possible complication and their severity will be recorded according to the specified criteria.

10.5. Data management

The data will be recorded manually in paper forms. Forms will be kept inside the binders/ clearbooks and lockers in the study setting and will only be accessible to authorized study personnel.
The required software will be provided for screening candidates and collecting and entering data, according to CRF data collection forms. Therefore, in addition to all paper forms, the data forms will be stored and recorded in the study databases online using a username and password.

10.6. Data monitoring

All collected data in electronic CRF, paper CRF and source of lab data will be monitored and any discrepancy will be reported to CRO.

11. Monitoring

11.1. Harms

Harms monitoring is important in the present study, and therefore, the FDA guidelines for timely reporting of such harms to the Ethics Committee and the FDA will be strictly observed as follows.

11.2. Safety Reporting Guidelines

These guidelines have been prepared and published by FDA .The relevant are summarized here.

11.2.1. Adverse Event (AE)

Adverse Event (AE) refers to any adverse medical event that occurs to participants while participating in this clinical trial. In this case, the event's occurrence does not necessarily have a causal relationship with the treatment protocols used in this study.
All AEs will be recorded and reviewed by DSMB for a causal relationship with the vaccine / IMP used in the study.
The following information will be collected for each AE in CRF.
- Event title / description
- Start date
- End date
- Intensity
- Treatments
- The result of the treatments
- Importance
- Vaccine / IMP-related measures

11.2.1.1. Expected Adverse Event

An expected adverse event is an event that has been predictable based on information contained in vaccine documentation such as the researcher's brochure. All expected adverse events will be recorded and reviewed by DSMB for a causal relationship with the studied vaccine / IMP.
Researchers will report cases to the Ethics and sponsor committee (Razi Vaccine and Serum Research Institute) if these cases lead to the exclusion of participants or have an unexpectedly higher prevalence (Table 2).

Table S3 Expected adverse event reporting guideline

Complication type	Reporting time period	Reporting procedure
The task of the researcher in the case of expected adverse event with an unexpectedly higher prevalence	As soon as the patient needs to withdraw the study or in the case of an obvious unexpectedly higher prevalence	Reporting by the researcher to the ethics committee and the pharmaceutical company (Sponsor)
The task of the sponsor company in the case of expected adverse event with an unexpectedly higher prevalence	Within 15 calendar days after notification of the pharmaceutical company (sponsor)	Reporting by the pharmaceutical company (Sponsor) to the authority approving the protocol (General Directorate of Drugs)

11.2.2. Serious Adverse Event / Reaction (SAE)

It is an event that occurs following vaccination and can lead to death, life threatening, hospitalization, prolonged hospital stay, permanent or significant disability, or congenital anomaly. Intervention to prevent any of the above should also be classified in the same category.
If the SAE results in the death of the patient or puts him/her at risk of death, the researcher will inform the Razi Vaccine and Serum Research Institute and the Ethics Committee in an appropriate and documented manner immediately and within 24 hours. If the risk of death is prevented by a slightly less severe action by the researcher, the case will be reported to the ethics committee and sponsor of the Razi Vaccine and Serum Research Institute immediately and within 7 days (Table 3).
The sponsor of the Razi Vaccine and Serum Research Institute will report all reports of SAE and follow-up results to the general directorate of FDA within 15 days of being notified.

Table S4. Serious adverse event reporting guide

Complication type	Reporting time period	Reporting procedure
Life-threatening or deadly SAE	Immediately, up to 24 hours after the researcher is aware of the SAE (via fax, e-mail, etc.)	Reporting by the researcher to the pharmaceutical company (Sponsor)<br>Reporting by the researcher to the ethics committee
Non-life-threatening or non-deadly SAE	Immediately, up to 7 calendar days after the researcher is aware of the SAE	Reporting by the researcher to the pharmaceutical company (Sponsor)<br>Reporting the report by the researcher to the ethics committee
All SAE and follow-up results	Supplementary report containing relevant information and follow-up results up to 15 calendar days after the pharmaceutical company (sponsor) is aware of the SAE	Reporting by the pharmaceutical company (Sponsor) to the general directorate of medicines

11.2.3. Suspected Unexpected Serious Adverse Reaction (SUSAR)

They are unexpected adverse events the characteristics, features and severity of which are not mentioned in the vaccine-related documentation and the researcher's brochure. These events are reported by the researcher based on the rules governing SAE. If SUSAR is deadly or life-threatening, the sponsor of the Razi Vaccine and Serum Research Institute shall report the matter, using the relevant form, to the Ethics Committee and the General Directorate of FDA as soon as possible and within a maximum of 7 days after notification.
If SUSAR of a slightly less severity leads to preventive action by the treatment team in a life-threatening situation, the above reporting time period will be increased to a maximum of 15 days (Table 4).

Table S5 Guide for suspected unexpected serious adverse reaction reporting

Complication type	Reporting time period	Reporting procedure
SUSAR was deadly or life-threatening	In the shortest time and up to 7 calendar days after the pharmaceutical company (sponsor) is aware of SUSAR.<br>Supplementary reporting of new information and follow-up results up to 15 calendar days after the pharmaceutical company (financial support) is aware of the SUSAR	Reporting by the pharmaceutical company (sponsor) to the ethics committee and the protocol approving authority (General Directorate of FDA)
SUSAR was not deadly or life-threatening	In the shortest possible time and up to 15 calendar days after the notification of the pharmaceutical company (Sponsor) of the SUSAR<br>Supplementary reporting of new information and follow-up results as soon as possible after the initial report	Reporting by the pharmaceutical company (sponsor) to the ethics committee and the protocol approving authority (General Directorate of FDA)

11.2.4. Medically Attended Adverse Events (MAAEs)

There are events that force participants to see a doctor immediately. All complications that are classified as severity grade 4 in Toxicity Tables in the Appendices section have this property. If one of these cases is reported in any way (daily / monthly telephone follow-up / candidate self-reporting during clinical visits), the relevant expert will collect detailed information in this regard, record in the CRF and report it to the principal investigator (PI) as soon as possible.

12. Ethics and Dissemination

12.1. Research Ethics Approval

The ethics approval of the present study will be obtained from the National Ethics Committee in Biomedical Research through the Food and Drug Organization in accordance with national guidelines.

12.2. Protocol Amendments

If amendments are needed in some parts of the protocol that affect the potential benefit from participating in the study or safety of candidates, the research team should revise and approve them and then submit them to the National Ethics Committee. These parts mainly include objectives, study design, study population, sample size, and actions performed on candidates during the study. Changes in the study establishing that do not affect the study as a whole will not need to be re-approved by the ethics committee. It should be noted that the Food and Drug Organization must approve any changes in the protocol.

12.3. Informed Consent

In the present study, informed consent will be obtained from all participants. At baseless, one of the study team members will provide the necessary explanations for the candidate regarding the process of study conduct and the number of visits for examining and taking human samples. It will be explained to candidates that their participation or non-participation will not have an unpleasant consequence in terms of receiving health services, and they can withdraw from the study whenever they want.
The informed consent form has been prepared according to the National Ethics Committee standards, which will be provided to the participants and their companions. Participants will be given sufficient time to study the form and make decisions and ask their questions. A flowchart of the visiting process (Appendix 5) will also be provided to them. If participants express their consent and sign the form, they will enter the study process.

12.4. Compensation

In order to compensate for possible vaccine-related harms, all participants have been covered by Iran Insurance with a contract worth 5300 million Rials. Participants will also be provided with transportation costs, complication recording equipment (mercury thermometer and ruler) as well as COVID-19 disease prevention equipment (including masks, gloves and alcohol). Each participant will be given a gift worth 2 million Rials for each visit at the end of the study.

12.5. Confidentiality

The principles of confidentiality regarding the collection, maintenance, and dissemination of information obtained from volunteers will be observed in this study. For this purpose, the codes assigned to the participants will be written on the data collection forms instead of their names. Participants' files will be available only to the research team and this information will be kept in a safe and secure place.
All documents will be kept confidential by the sponsor for five years after the end of the study.

12.6. Dissemination policy

The results of the present study will be reported to the Food and Drug Administration of Iran. They will also be published as articles in scientific journals of relevant fields.
Parts of the protocol that do not contain confidential information from sponsors and researchers will be made available to the public along with the results.

12.7. Additional Considerations

12.7.1. Vaccine/IMP- related complications

The study setting is a part of the hospital where the infection risk is minimized because of allocation of a separate entrance. There is also the least distance between the study setting and emergency department of the hospital since an

ambulance is also dedicated to this project. However, the following are taken into account to ensure minimization of the immediate threats following vaccination:
1. A separate room is provided in the inpatient wards to take care of the study participants. This clause is included in the contract between Rasoul Akram Hospital and the sponsor and costs are undertaken by the sponsor.
2. The existence of an emergency department at the vaccination site with suitable facilities to manage possible cases
3. Existence of a special study ambulance in front of the entrance door of the study setting
4. Monitoring all candidates for at least three hours after vaccination, measuring vital signs every one hour in phases I and II for at least 2 hours after vaccination, and measuring the vital signs every 40 minutes
5. Daily telephone follow-up of all candidates within the first week after vaccination
6. Monthly telephone follow-up of all candidates up to 6 months after vaccination
7. Considering an on-call physician and providing a mobile phone line to all candidates

In case of emergencies, the person is immediately referred to Rasoul Akram Hospital by the physician attending the center or an on-call physician (depending on the timing of the vaccine-related complication) and the necessary medical measures are taken for the person. Since all candidates will be covered by insurance as soon as they receive the randomization code, the costs related to the study processes will be reimbursed by the insurance.

13. Statistical methods

13.1. Sample Size

The sample size is based on practical considerations and expert opinion and not standard formulas and statistical power calculations. In the present study, there are four different vaccine / IMP groups (n= 30 people per group). In addition, 13 people entered the study as sentinel participants, and 20 non-randomized open-labeled to assess secretory IgA. Therefore, in total, the sample size in phase I will be 153 people.
If participants refuse to cooperate in the study, those who were excluded from the study at baseline will be replaced by eligible individuals. People who withdraw from the study after the first or second vaccine / IMP dose are encouraged for safety follow-up.
The sample size in phase II of the study is predicted to be 500 people, which is again based on practical considerations and expert opinion. Participants will receive randomly either adjuvant only or the selected dose of Phase I. This strength will be determined from the data obtained from the interim analysis after the 35th day of phase I and will be operationalized after being approved by the DSMB. Participants in phase II will receive two doses of IM intervention (day 0 and 21) followed by one intranasal intervention at day 51.

13.2. Definition

13.2.1. Safety population

All candidates who received a single IMP / vaccine dose in the study will be considered as a safety population.

13.2.2. Immunogenicity population

Effectiveness is interpreted as immunogenicity in this study, and therefore, all candidates who have received at least the first and second vaccine dose and have at least one sample to assess immunogenicity at the appropriate time and committed no protocol deviation will be considered as an immunogenicity population.

13.3. Analysis approach

The main analysis approach in this study will be intention to treat (ITT). If the protocol deviation occurs for any reason, the outcome data will be collected as much as possible until the end and will be considered in the analysis stage. Finally, the results of the per-protocol analysis will be extracted and compared with the results of the first approach. In the ITT approach, each subject will be classified into comparison groups based on random allocation at baseline, and if they receive at least one vaccine / IMP dose and a subsequent blood sample, it will be included in the analysis. The researcher will exclude candidates from the analysis in per protocol approach who have withdrawn from the study for some reason or have changed their study group.

13.4. Analysis plan

First, a descriptive analysis of the study variables will be carried out. All variables collected at baseline will be compared in four groups in phase I and two groups in phase II to ensure the random assignment. The collected data will be sorted and then analyzed using SPSS and STATA statistical software. P-value<0.05 will be considered as the

significance level in all tests. For Safety and immunogenicity data, descriptive analysis will first be conducted. These data will then be compared into four groups. Chi-square test and, if necessary, Fisher's Exact test will be used to compare ratios. The immunogenicity findings will be analyzed by logarithmically converting the measured titers into geometric mean titers. Independent t-test will be used to compare continuous quantitative data with normal distribution in the two groups and non-parametric Mann-Whitney U test will be used to compare quantitative rank data with non-normal distribution.

The "serum level using geometric mean" variable is the geometric mean of the antibody titer against Spike antigen, which will be compared in the control group with the vaccine groups. The "seroconversion rate" variable will be calculated and compared by evaluating the fold increase in serum levels. The seroconversion rate is defined as the proportion of those whose serum IgG levels have quadrupled or increased using the ELISA method. The number and percentage of these people in the vaccine groups will be compared with the control group.

The study will be stopped, and phase II will not start if one of the following cases occurs:

- Occurrence of any serious vaccine-related complication (SAE) following vaccine / IMP injection
- Occurrence of serious local toxicity, vital or systemic symptoms (Grade 3 according to Tables 8) in more than 30% of each intervention group one month after each vaccine / IMP dose
- Occurrence of severe laboratory toxicity (Grade 3 according to Tables9-11) in more than 30% of each intervention group one month after each vaccine / IMP dose. Tests include creatinine, AST, ALT, bilirubin, hemoglobin, complete white blood cell and platelet count, and urine tests.

13.5. Missing Data

No imputation will be performed for missing data.

13.6. Interim analysis

Considering the current COVID-19 crisis in Iran and the need for faster access to an effective vaccine, an interim analysis will be performed on all participants in phase I, 14 days after the second vaccine dose (35 days after receiving the first dose). Based on the results of this analysis, a decision will be made regarding the start of phase II.

In the present study, the members of the DSMB and FDA will decide on the initiation of phase II. This decision will be based on the evidence of safety and immunogenicity on day 35 in phase I (two weeks after the second vaccine dose). If none of the study discontinuation criteria listed on section 8 occur and the neutralizing antibody response occurs in more than 50% of participants, a request to start phase II will be sent to the FDA after the DSMB approval.

The criterion for selecting the final antigen concentration to enter phase II will be the concentration that causes the lowest incidence and lowest severity of side effects in general, especially in terms of SAEs, and induces higher immunogenicity, especially cell-mediated immunity and neutralizing antibody titer.

After phase II (the selected dose) starts, an interim analysis will be performed two weeks after the second vaccine dose like phase I. The above analysis will be carried out with the difference when the first 150 people (30%) of the participants finish their 35th-day visit. If none of the study- discontinuation criteria listed on section 8 occur in phase II participants and the neutralizing antibody response occurs in more than 30% of participants, a request on the start of Phase III will be sent to the FDA after DSMB approval.

References

1. Hale T, Angrist N, Goldszmidt R, Kira B, Petherick A, Phillips T, et al. A global panel database of pandemic policies (Oxford COVID-19 Government Response Tracker). Nature Human Behaviour. 2021;5(4):529-38.
2. WHO. The COVID-19 vaccine tracker and landscape compiles detailed information of each COVID-19 vaccine candidate in development by closely monitoring their progress through the pipeline. 2023 [Available from: https://www.who.int/publications/m/item/draft-landscape-of-covid-19-candidate-vaccines.
3. Richmond P, Hatchuel L, Dong M, Ma B, Hu B, Smolenov I, et al. Safety and immunogenicity of S-Trimer (SCB-2019), a protein subunit vaccine candidate for COVID-19 in healthy adults: a phase 1, randomised, double-blind, placebo-controlled trial. Lancet. 2021;397(10275):682-94.
4. Goepfert PA, Fu B, Chabanon A-L, Bonaparte MI, Davis MG, Essink BJ, et al. Safety and immunogenicity of SARS-CoV-2 recombinant protein vaccine formulations in healthy adults: interim results of a randomised, placebo-controlled, phase 1–2, dose-ranging study. The Lancet Infectious Diseases. 2021.
5. Bleier BS, Ramanathan M, Lane AP. COVID-19 Vaccines May Not Prevent Nasal SARS-CoV-2 Infection and Asymptomatic Transmission. Otolaryngology–Head and Neck Surgery. 2021;164(2):305-7.
6. Hassan AO, Feldmann F, Zhao H, Curiel DT, Okumura A, Tang-Huau T-L, et al. A single intranasal dose of chimpanzee adenovirus-vectored vaccine protects against SARS-CoV-2 infection in rhesus macaques. Cell Reports Medicine. 2021;2(4):100230.
7. Ku M-W, Bourgine M, Authié P, Lopez J, Nemirov K, Moncoq F, et al. Intranasal vaccination with a lentiviral vector protects against SARS-CoV-2 in preclinical animal models. Cell Host & Microbe. 2021;29(2):236-49.e6.
8. Du Y, Xu Y, Feng J, Hu L, Zhang Y, Zhang B, et al. Intranasal administration of a recombinant RBD vaccine induced protective immunity against SARS-CoV-2 in mouse. Vaccine. 2021;39(16):2280-7.
9. van Doremalen N, Purushotham JN, Schulz JE, Holbrook MG, Bushmaker T, Carmody A, et al. Intranasal ChAdOx1 nCoV-19/AZD1222 vaccination reduces viral shedding after SARS-CoV-2 D614G challenge in preclinical models. Science Translational Medicine. 2021:eabh0755.
10. van Doremalen N, Purushotham J, Schulz J, Holbrook M, Bushmaker T, Carmody A, et al. Intranasal ChAdOx1 nCoV-19/AZD1222 vaccination reduces shedding of SARS-CoV-2 D614G in rhesus macaques. bioRxiv. 2021:2021.01.09.426058.
11. Alu A, Chen L, Lei H, Wei Y, Tian X, Wei X. Intranasal COVID-19 vaccines: From bench to bed. EBioMedicine. 2022;76:103841.
12. FDA. Guidance for industry: toxicity grading scale for healthy adult and adolescent volunteers enrolled in preventive vaccine clinical trials. Food and Drug Administration, US Department of Health and Human Services2007.

14. Appendix

Appendix 1. Abbreviation Table for protocol and supplementary

AE	Adverse Event
ACE2	Angiotensin Converting Enzyme-2
ARDS	Acute Respiratory Distress Syndrome
B-hCG	beta-human chorionic gonadotropin
BUN	blood urea nitrogen
CFSE	Carboxy Fluorescein Succinimidyl Ester
CD4	Cluster of Differentiation 4 0
CD8	Cluster of Differentiation 8
CHO	Chinese Hamster Ovary
COVID-19	Corona Virus Disease -2019
COPD	Chronic Obstructive Pulmonary Disease
CRF	Case Report Form
CRO	Contract Research Organization
DPP4	Dipeptidyle Peptidase-4
DSMB	Data and Safety Monitoring Board
EADRs	Expected Adverse Drug Reactions
EFD	Embryo-Fetal Development
FDA	Iran Food and Drug Administration
GCP	Good Clinical Practice
GMP	Good Manufacturing Practices
HBsAg	Hepatitis B surface antigen
HBcAb	Hepatitis B core antibody
HCV	hepatitis C virus
HEK	Human Embryonic Kidney
IMP	Investigational Medicinal Product
INFγ	Interferon gamma
ITT	Intention To Treat
IL4	Interleukin 4
IUMS	Iran University of Medical Sciences
M	Membrane protein
MAAE	Medically Attended Adverse Event
MERS	Middle East Respiratory
N	Nucleocapside protein
NHP	Non-Human Primate
ORF	Open Reading Frames
PBMC	Peripheral Blood Mononuclear Cell
PBS	Phosphate Buffered Saline
RBD	Receptor Binding Domain
RVSRI	Razi Vaccine and Serum Research Institute
S	Spike glycoprotein
SAE	Serious Adverse Events
SARS	Severe Acute Respiratory Syndrome
SOP	Standard Operating Procedures
SUSAR	Suspected Unexpected Serious Adverse Reaction
Th1/2	T helper type 1/2

Appendix 2 .Toxicity scoring

Toxicity severity scoring will be carried out according to US Food and Drug Administration guidelines (12). This classification is intended for use in adults and adolescents.

Table S6. Classifying the severity of local adverse reactions

Local Reaction to Injectable Product	Mild (Grade 1)	Medium(Grade 2)	Severe (Grade 3)	Potentially Life Threatening (Grade 4)
Pain	Does not interfere with activity	Repeated use of non-narcotic pain reliever > 24 hours or interferes with activity	Any use of narcotic pain reliever or prevents daily activity	Emergency room (ER) visit or hospitalization
Tenderness	Mild discomfort to touch	Discomfort with movement	Significant discomfort at rest	ER visit or hospitalization
Erythema/Redness *	2.5 – 5 cm	5.1 – 10 cm	> 10 cm	Necrosis or exfoliative dermatitis
Induration/Swelling **	2.5 – 5 cm and does not interfere with activity	5.1 – 10 cm or interferes with activity	> 10 cm or prevents daily activity	Necrosis

* In addition to grading the measured local reaction at the greatest single diameter, the measurement should be recorded as a continuous variable. ** Induration/Swelling should be evaluated and graded using the functional scale as well as the actual measurement.

Table S7. Classifying the severity of adverse reactions based on vital signs

Vital Signs *	Mild (Grade 1)	Medium (Grade 2)	Severe (Grade 3)	Potentially Life Threatening (Grade 4)
Fever (°C) **	38.0 – 38.4	38.5 – 38.9	39.0 – 40	> 40
Tachycardia - beats per minute	101 – 115	116 – 130	> 130	ER visit or hospitalization for arrhythmia
Bradycardia - beats per minute***	50 – 54	45 – 49	< 45	ER visit or hospitalization for arrhythmia
Hypertension (systolic) - mm Hg	141 – 150	151 – 155	> 155	ER visit or hospitalization for malignant hypertension
Hypertension (diastolic) - mm Hg	91 – 95	96 – 100	> 100	ER visit or hospitalization for malignant hypertension
Hypotension (systolic) – mm Hg	85 – 89	80 – 84	< 80	ER visit or hospitalization for hypotensive shock
Respiratory Rate – breaths per minute	17 – 20	21 – 25	> 25	Intubation

* Subject should be at rest for all vital sign measurements. ** Oral temperature; no recent hot or cold beverages or smoking. *** When resting heart rate is between 60 – 100 beats per minute. Use clinical judgement when characterizing bradycardia among some healthy subject populations, for example, conditioned athletes.

Table S8. Classifying the severity of systemic adverse reactions

Systemic (General)	Mild (Grade 1)	Medium(Grade 2)	Severe (Grade 3)	Potentially Life Threatening (Grade 4)
Nausea/vomiting	No interference with activity or 1 – 2 episodes/24 hours	Some interference with activity or > 2 episodes/24 hours	Prevents daily activity, requires outpatient IV hydration	ER visit or hospitalization for hypotensive shock
Diarrhea	2 – 3 loose stools or < 400 gms/24 hours	4 – 5 stools or 400 – 800 gms/24 hours	6 or more watery stools or > 800 gms/24 hours or requires outpatient IV hydration	ER visit or hospitalization
Headache	No interference with activity	Repeated use of non-narcotic pain reliever > 24 hours or some interference with activity	Significant; any use of narcotic pain reliever or prevents daily activity	ER visit or hospitalization

Fatigue	No interference with activity	Some interference with activity	Significant; prevents daily activity	ER visit or hospitalization
Myalgia	No interference with activity	Some interference with activity	Significant; prevents daily activity	ER visit or hospitalization
Illness or clinical adverse event (as defined according to applicable regulations)	No interference with activity	Some interference with activity not requiring medical intervention	Prevents daily activity and requires medical intervention	ER visit or hospitalization

Table S9. Classifying the severity of adverse reactions based on laboratory criteria (serum)

**Serum ***	**Attributable to lab error/pop norms**	**Mild (Grade 1)**	**Medium (Grade 2)**	**Severe (Grade 3)**	**Potentially Life Threatening (Grade 4)****
Sodium – Hyponatremia mEq/L	136-145	132 – 135	130 – 131	125 – 129	< 125
Sodium – Hypernatremia mEq/L	136-145	146 – 147	148– 149	150 – 151	> 151
Potassium – Hyperkalemia mEq/L	3.7- 5.5	5.6 – 5.7	5.8 – 5.9	6 – 6.1	> 6.1
Potassium – Hypokalemia mEq/L		3.5 – 3.6	3.3 – 3.4	3.1 – 3.2	< 3.1
Glucose – Hypoglycemia mg/dL		65 – 69	55 – 64	45 – 54	< 45
Glucose – Hyperglycemia<br>Fasting – mg/dL<br>Random – mg/dL		100 – 110<br>110 – 125	111 – 125<br>126 – 200	>125<br>>200	Insulin requirements or hyperosmolar coma
Blood Urea Nitrogen<br>BUN mg/dL		23 – 26	27 – 31	> 31	Requires dialysis
Creatinine – mg/dL		1.5 – 1.7	1.8 – 2.0	2.1 – 2.5	> 2.5 or requires dialysis
Calcium – hypocalcemia mg/dL		8.0 – 8.4	7.5 – 7.9	7.0 – 7.4	< 7.0
Calcium – hypercalcemia mg/dL		10.5 – 11.0	11.1 – 11.5	11.6 – 12.0	> 12.0
Magnesium – hypomagnesemia mg/dL		1.3 – 1.5	1.1 – 1.2	0.9 – 1.0	< 0.9
Phosphorous – hypophosphatemia mg/dL		2.3 – 2.5	2.0 – 2.2	1.6 – 1.9	< 1.6
CPK – mg/dL		1.25 – 1.5 x ULN***	1.6 – 3.0 x ULN	3.1 –10 x ULN	> 10 x ULN
Albumin – Hypoalbuminemia g/dL		2.8 – 3.1	2.5 – 2.7	< 2.5	--
Total Protein – Hypoproteinemia g/dL		5.5 – 6.0	5.0 – 5.4	< 5.0	--
Alkaline phosphate – increase by factor		1.1 – 2.0 x ULN	2.1 – 3.0 x ULN	3.1 – 10 x ULN	> 10 x ULN
Liver Function Tests –ALT, AST<br>increase by factor		1.5 – 2.5 x ULN	2.6 – 5.0 x ULN	5.1 – 10 x ULN	> 10 x ULN
Bilirubin – when accompanied by any increase in Liver Function Test<br>increase by factor		1.5 – 1.75x ULN	1.76– 2x ULN	2.1– 2.25x ULN	> 2.25x ULN
Bilirubin – when Liver Function Test is normal; increase by factor		1.5 – 2x ULN	2.1 – 2.5x ULN	2.6 – 3.0 x ULN	> 3.0 x ULN
Cholesterol		201 – 210	211 – 225	> 226	---
Pancreatic enzymes – amylase, lipase		1.1 – 1.5 x ULN	1.6 – 2.0 x ULN	2.1 – 5.0 x ULN	> 5.0 x ULN

* The laboratory values provided in the tables serve as guidelines and are dependent upon institutional normal parameters. Institutional normal reference ranges should be provided to demonstrate that they are appropriate. ** The clinical signs or symptoms associated with laboratory abnormalities might result in characterization of the laboratory abnormalities as Potentially Life Threatening (Grade 4). For example, a low sodium value that falls within a grade 3 parameter (125-129 mE/L) should be recorded as a grade 4 hyponatremia event if the subject had a new seizure associated with the low sodium value. ***ULN" is the upper limit of the normal range.

Table S10. Classifying the severity of adverse reactions based on laboratory criteria (hematology)

**Hematology ***	**Attributable to lab error/pop norms**	**Mild (Grade 1)**	**Medium (Grade 2)**	**Severe (Grade 3)**	**Potentially Life Threatening (Grade 4)**

Hemoglobin (Female) - gm/dL	11-12	10.5 - 0.9	9.5 – 10.4	8.0 – 9.4	< 8.0
Hemoglobin (Female) change from baseline value - gm/dL		Any decrease – 1.5	1.6 – 2.0	2.1 – 5.0	> 5.0
Hemoglobin (Male) - gm/dL		12.5 – 13.5	10.5 – 12.4	8.5 – 10.4	< 8.5
Hemoglobin (Male) change from baseline value – gm/dL		Any decrease – 1.5	1.6 – 2.0	2.1 – 5.0	> 5.0
WBC Increase - cell/mm^3		10,800 – 15,000	15,001 – 20,000	20,001 – 25, 000	> 25,000
WBC Decrease - cell/mm^3		2,500 – 3,500	1,500 – 2,499	1,000 – 1,499	< 1,000
Lymphocytes Decrease - cell/mm^3		750 – 1,000	500 – 749	250 – 499	< 250
Neutrophils Decrease - cell/mm^3		1,500 – 2,000	1,000 – 1,499	500 – 999	< 500
Eosinophils - cell/mm^3		650 – 1500	1501 - 5000	> 5000	Hypereosinophilic
Platelets Decreased - cell/mm^3		125,000 – 140,000	100,000 – 124,000	25,000 – 99,000	< 25,000
PT – increase by factor (prothrombin time)		1.0 – 1.10 x ULN**	1.11 – 1.20 x ULN	1.21 – 1.25 x ULN	> 1.25 ULN
PTT – increase by factor (partial thromboplastin time)		1.0 – 1.2 x ULN	1.21 – 1.4 x ULN	1.41 – 1.5 x ULN	> 1.5 x ULN
Fibrinogen increase - mg/dL		400 – 500	501 – 600	> 600	--
Fibrinogen decrease - mg/dL		150 – 200	125 – 149	100 – 124	< 100 or associated with gross bleeding or (DIC)

Table S11. Classifying the severity of adverse reactions based on laboratory criteria (urine)

**Urine ***	**Attributable to lab error**	**Mild (Grade 1)**	**Medium (Grade 2)**	**Severe (Grade 3)**	**Potentially Life Threatening (Grade 4)**
Protein	-	Trace	1+	2+	Hospitalization or dialysis
Glucose	-	Trace	1+	2+	Hospitalization for hyperglycemia
Blood (microscopic) – red blood cells per high power field (rbc/hpf)	1-5	6 - 10	11 – 50	> 50 and/or gross blood	Hospitalization or packed red blood cells (PRBC) transfusion

* The laboratory values provided in the tables serve as guidelines and are dependent upon institutional normal parameters. Institutional normal reference ranges should be provided to demonstrate that they are appropriate.

Appendix 3. COVID-19 case definition

Suspected case

A. A disease that has clinical signs and epidemiological criteria:

Clinical findings:

Sudden onset of fever and cough

Or

Sudden onset of at least three or more symptoms such as fever, cough, general weakness / extreme fatigue, headache, muscle aches, sore throat, runny nose, shortness of breath, anorexia / nausea / vomiting, diarrhea, loss of consciousness

Epidemiological evidence

Accommodation, employment or travel to areas with high risk of infection (such as accommodation centers, crowded places, conferences and events, healthcare centers, etc.) during the past 14 days

B. A person with acute respiratory disease (SARS), the onset of symptoms during the last 10 days, urgent need to hospital admission

Probable case

A. A patient with a suspected infection who is in contact with a patient with probable or confirmed infection or a cluster of patients among whom at least one confirmed case has been reported.

B. A patient with a probable infection and positive Covid-19 infection based on imaging findings

• Such as unilateral or bilateral multinodular infiltration, especially infiltration of peripheral areas in CT scan of the lung or chest radiography and grounded glass lung (Clinically confirmed)
C. A patient who has severely lost his sense of smell or taste
D. Death in a patient with suspected Covid-19 (above criteria) that is not justified by any other reason

Confirmed case
A person with laboratory confirmation of the Covid-19 virus, regardless of the presence of clinical signs and symptoms

Close contact
A person who has been in contact with a patient with probable or confirmed infection in the following circumstances, within 2 days before to 14 days after the onset of symptoms, including:
1. Face to face contact at a less-than-1-meter distance for at least 15 minutes
2. Direct physical contact with a person with probable or confirmed infection
3. Caring for a patient with a probable or confirmed infection without the use of appropriate personal protective equipment
Or
4. In other cases, the evaluation is based on the probability of regional transmission.

Appendix 4. Intramuscular vaccine injection procedure

Vaccine preparation:

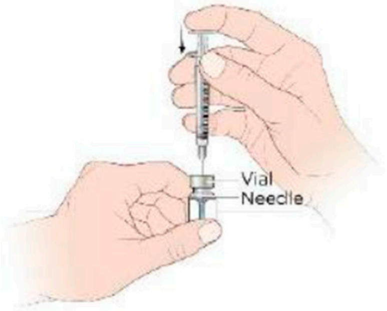

Insert the needle into the rubber top

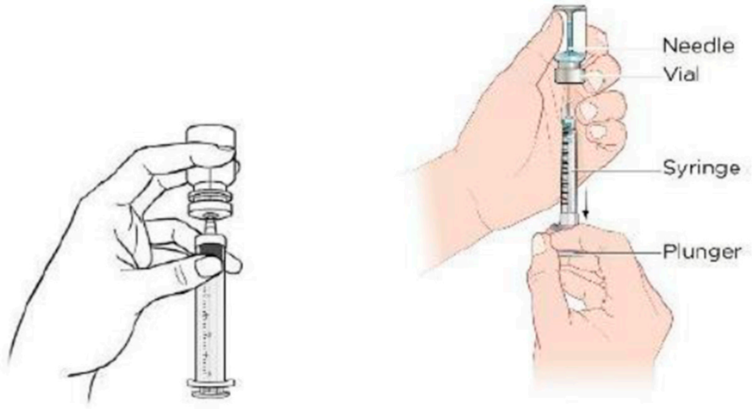

Draw the required dose with a syringe

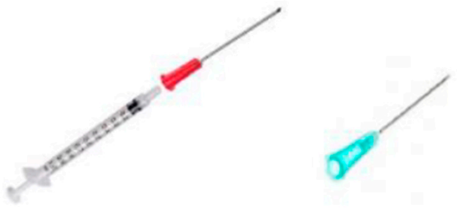

Change the needle

Injection method:

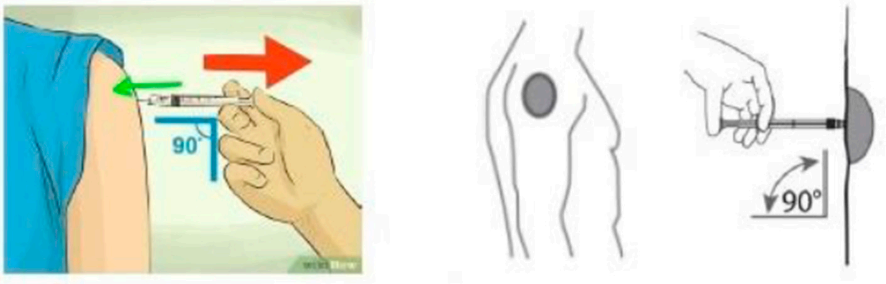

Insert the needle into the deltoid muscle at a 90 degree angle

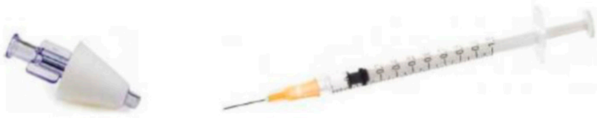

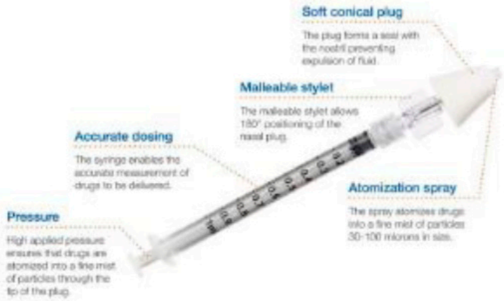

B.

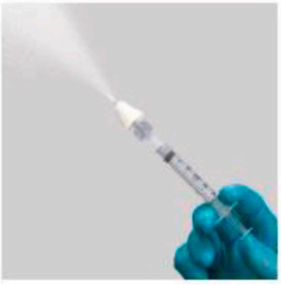

C.

Appendix 5. The visiting process for vaccination in Phase I

IUMS Clinical Trial Centre

Razi Vaccine & Serum Research Institute

Dear volunteer

Thank you for your participation in the study of phase I of SARS-CoV2 -recombinant Spike protein vaccine produced by Razi Institute, the steps of this study are as follows:

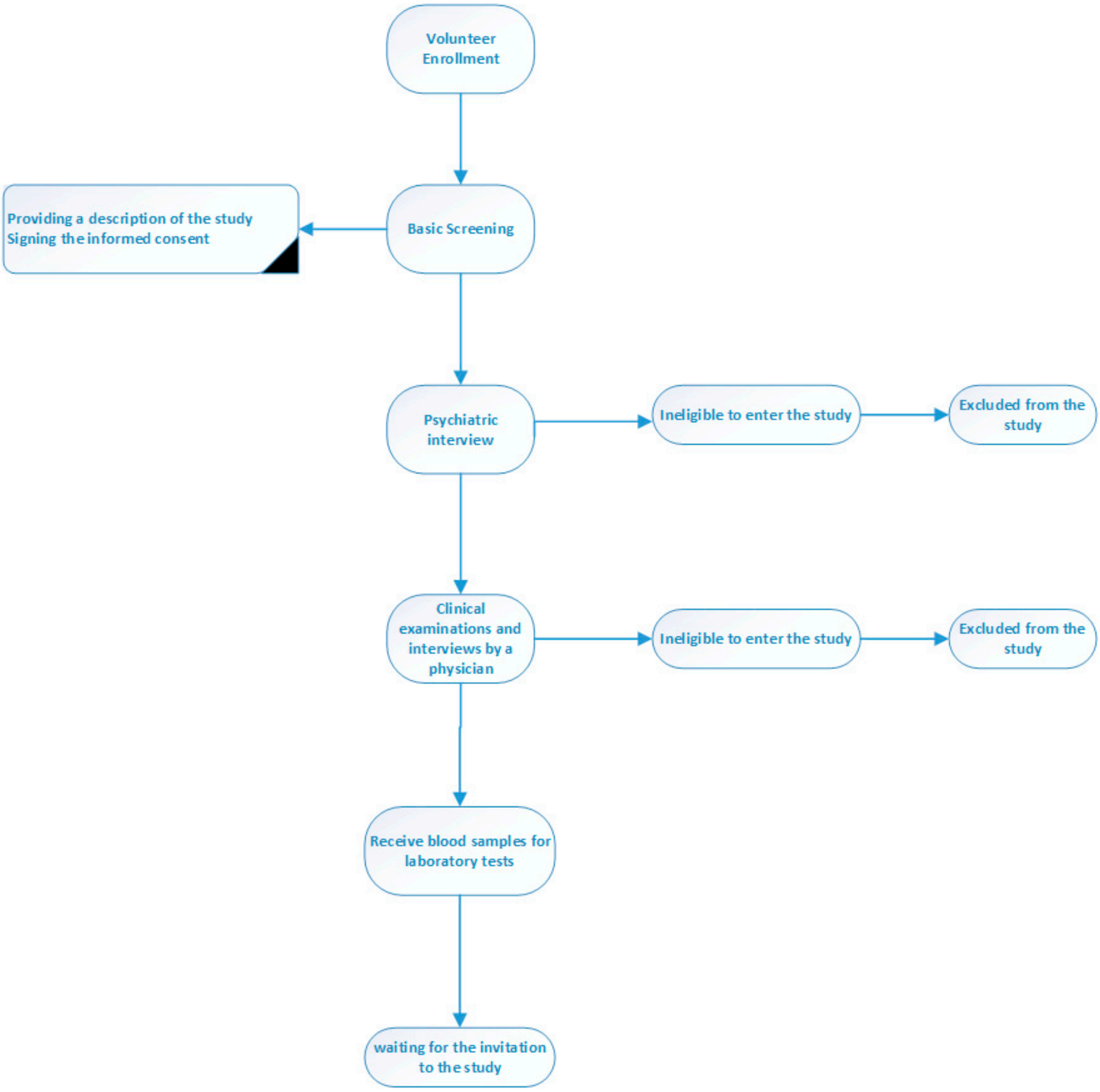

IUMS CTC
IUMS Clinical Trial Centre

Process of participants visiting to carry out the study of phase I of SARS-CoV2 -recombinant spike protein vaccine developed by Razi Institute

Razi Vaccine & Serum Research Institute

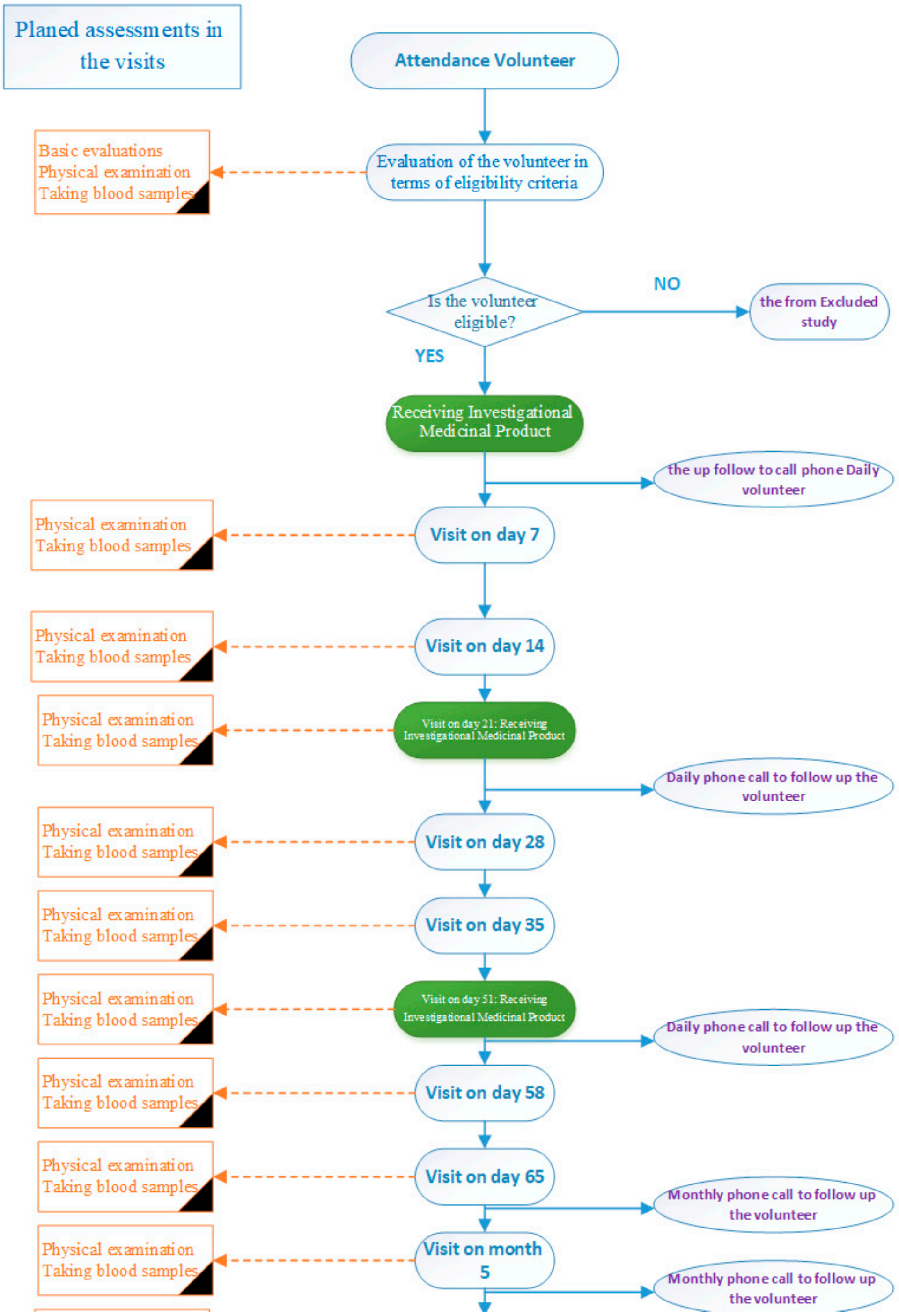

Supplement: Supplementary file 1 [file vaccines-11-00455-s001.zip › vaccines-2172159 -Supplemantary file-Protocol Phase I, II,-2.15 conversion.pdf]
