# Supplementary material for: Immunogenicity and Safety of a Combined Intramuscular/Intranasal Recombinant Spike Protein COVID-19 Vaccine (RCP) in Healthy Adults Aged 18 to 55 Years Old: A Randomized, Double-Blind, Placebo-Controlled, Phase I Trial
Source: Vaccines (Basel). 2023 Feb 16;11(2):455. doi: 10.3390/vaccines11020455 (PMC9961243; doi:10.3390/vaccines11020455)
Supplement: Supplementary file 1 [file vaccines-11-00455-s001.zip › vaccines-2172159-sup-tables and figure -2.15conversion.pdf]

Immunogenicity and safety of a combined intramuscular/intranasal recombinant spike protein COVID-19 vaccine (RCP) in healthy adults aged 18 to 55 years old: a randomized, double-blind, placebo-controlled, phase I trial

Table of Contents

I- Supplemental Method 3

Vaccination Pause Rules 3

Determination of immunogenicity 3

Enzyme-linked immunosorbent assays (ELISA) 3

Saliva collection for assessment of RBD specific IgA antibody using ELISA 3

Virus Neutralizing Test (VNT) 3

Human ACE2 Protein (hACE-2) Binding Assay 4

Evaluation the Cellular Immune Response by CFSE proliferation assay 4

Evaluation the cell markers and intracellular IFN-γ of MNCs in vaccinated human 4

Evaluation of the cytokine levels by ELISA 4

Classification of toxicity 4

Table S1 FDA toxicity grading scale for vital sign abnormalities· 4

Table S2 FDA toxicity grading scales for solicited local and systemic adverse events 5

Table S3 FDA toxicity grading scales for clinical laboratory abnormalities 5

Table S4: World Health Organisation-Uppsala Monitoring Centre causality assessment scale 7

Table S5: Predefined time schedule of the study. 8

II-Supplemental Study Results 8

Demographic characteristic 8

Table S6 Demographic characteristics of the sentinel participants 8

Table S7Geometric mean ratio and 95% CI of specific antibody responses (AUC) to S, S1, S2, RBD and NTD antigens (Variant in Wuhan) in the intervention groups over the predefined study time schedule. 9

Table S8 Serum levels of specific antibodies against S, S1, S2, RBD, NTD and N antigens (Variant in Wuhan) in trial participants at enrollment. 9

Table S9 Number and percentages of subjects experiencing solicited local and systemic adverse events vaccination dose, by FDA toxicity grade 10

Table S10 Number and percentages of subjects experiencing solicited systemic adverse events after third dose of vaccine, by FDA toxicity grade 11

Table S11 List of adverse events, their grades and causal relationship with the intervention received (sorted by grade) during the six-month follow-up period. 12

Table S12 List of patients with positive COVID-19 PCR test result during the follow up period 13
Table S13 Abnormal vital signs during 3 hours after dose 1 and 2 14
Table S14 Number and percentages of subjects experiencing laboratory abnormalities by FDA toxicity grade 16
Table S15 Geometric means of IgG antibody responses (presented as area under the curve, AUC) against S antigen (Variant in Wuhan) in the intervention groups over the predefined study time schedule 18
Table S16 Geometric means of IgG antibody responses (presented as area under the curve, AUC) against S1 antigen (Variant in Wuhan) in the intervention groups over the predefined study time schedule 19
Table S17 Geometric means of IgG antibody responses (presented as area under the curve, AUC) against S2 antigen (Variant in Wuhan) in the intervention groups over the predefined study time schedule 20
Table S18 Geometric means of IgG antibody responses (presented as area under the curve, AUC) against RBD antigen (Variant in Wuhan) in the intervention groups over the predefined study time schedule 21
Table S19 Geometric means of IgG antibody responses (presented as area under the curve, AUC) against NTD antigen (Variant in Wuhan) in the intervention groups over the predefined study time schedule 22
Table S20 Geometric means of IgG antibody responses (presented as area under the curve, AUC) against N antigen (Variant in Wuhan) in the intervention groups over the predefined study time schedule 23
Table S21 Geometric mean titer for neutralizing antibody titer the over the predefined study time schedule 24
Figure S1 Geometric means of IgG antibody responses (presented as area under the curve, AUC) against S antigen in the intervention groups over the predefined study time schedule 25
Figure S2 Geometric means of IgG antibody responses (presented as area under the curve, AUC) against S1 antigen in the intervention groups over the predefined study time schedule 26
Figure S3 Geometric means of IgG antibody responses (presented as area under the curve, AUC) against S2 antigen in the intervention groups over the predefined study time schedule 27
Figure S4 Geometric means of IgG antibody responses (presented as area under the curve, AUC) against RBD antigen in the intervention groups over the predefined study time schedule 28
Figure S5 Geometric means of IgG antibody responses (presented as area under the curve, AUC) against NTD antigen in the intervention groups over the predefined study time schedule 29
Figure S6 Scatter plots illustrating the correlation between neutralizing antibody responses and specific IgG ELISA antibody responses (AUC) at 2 weeks after the second dose (day 35) in the intervention groups. Nonparametric Spearman correlation estimates have been shown on the diagrams. 30
Figure S7 Scatter diagram of changes in percentage of CD3,CD4 and CD3,CD8 (cytotoxic) T cells in response to stimulation by S antigen measured by flow cytometry in peripheral blood mononuclear cell (PBMC) extract in the intervention groups at the day 35 compared to the baseline. Percentage means have been shown on the diagram. 31

References 32

I- Supplemental Method

Vaccination Pause Rules

1· Occurrence of any severe vaccine-related complication (SAE) following vaccine / IMP injection
2· Incidence of severe local or systemic toxicity, or abnormal vital signs (grade 3) in more than 30% in each intervention group one month after each vaccine / IMP dose
3· Incidence of severe laboratory toxicity (grade 3) in more than 30% in each intervention group one month after each vaccine / IMP dose

Determination of immunogenicity

Enzyme-linked immunosorbent assays (ELISA)

The full S, S1, S2, RBD, N, and N- terminal domain (NTD) specific IgG antibody titer of the collected serum samples were evaluated through in house ELISA. Briefly, 96-well high-binding plates (Greiner, Austria) were coated with 100-ngr/well of S1 and RBD antigens diluted in sterile carbonate buffer at 4°C overnight. Following standard blocking with 3% of skimmed milk for 2 hours at 37°C, it was washed. Afterwards, the plates were incubated with the collected sera at a dilution of 1:100 up to 100000 for 1 hour at 37° C in duplicates. After being washed three times, anti-human IgG -HRP conjugates were diluted and used as secondary antibodies (Sigma, USA) followed by washing and detection with 3,5,3′,5′-tetramethylbenzidine (Millipore, USA). The OD value (450 nm) was read via Cytation 5 imaging reader (BioTek, USA). The results are expressed as area under curve (AUC).

Saliva collection for assessment of RBD specific IgA antibody using ELISA

Twenty different participants were recruited to investigate immune stimulation of the upper respiratory tract. After receiving two doses of 10-µg IM vaccine, they were assigned (non-random and open abled) to receiving either adjuvant only or 10-µg/200µl intranasal vaccine on day 51. Saliva sampling was performed before receiving first dose of vaccine in day 0, and at predefined intervals (day 65, 120, and 150), and they were used to evaluate the level of IgA antibodies against RBD antigen by enzyme linked immune-sorbent assay (ELISA). The collected saliva samples were centrifuged at 4000 rpm for 10 minutes in 4ºC. Following this step, samples were treated with diluted Triton X-100 solution for inactivation of the virus. After inactivation, saliva samples were stored in -80ºC overnight. A day after, the samples were centrifuged at 15,000 rpm for 10 minutes in 4ºC; the supernatant was then immediately transferred to a clean tube, allocated and stored in -20ºC until examined by ELISA assay. Antigen concentrations considered in the study included 100-ng/well of RBD in Carbonate-Bicarbonate buffer, pH 9·6 which were coated on 96-well ELISA plates (Greiner) and incubated overnight for 24 hours at 4°C. Plates were washed once and blocked with 200-µL/well of 3% skimmed milk (Sigma, USA) for 3 hours at 37°C. After being washed once with PBS, the plates were then incubated with 100-µL/well of saliva with dilution of 1/10 in 3% skimmed milk for 2 hours at 37°C. The wells were then washed three times with 10 seconds of soaking time. Following the washing step, incubation was performed with 100-µL/well of Anti human IgA HRP (Sigma, USA) diluted to 1: 5,000 in 3% skimmed milk and incubated at 37°C for 2 hours. The plates were washed four times with PBS-T, followed by which TMB (Sigma, USA). The OD value (450-nm) was read by Cytation 5 imaging reader (BioTek, USA). Duplicate analyses were performed on each sample.

Virus Neutralizing Test (VNT)

The virus neutralization test was performed to evaluate the protective properties of RCP recombinant spike protein COVID-19 vaccine. SARS-CoV-2 (GISAID accession EPI_ISL_1398937) was isolated from the clinical human specimens in this study. The titration of virus was performed from 1 log to 11 log (in serial 1 log dilutions) in order to obtain a 50% tissue culture infective dose (TCID50) on 96-well culture plates of VERO cells. The serum samples obtained from all the participants were heat-inactivated at 56°C for 30 minutes. Two-fold serial dilutions of serum samples with the starting dilution of 1:4 (i.e., 1/4, 1/8, 1/16, 1/32, 1/64) were then mixed with an equal volume of 100 TCID50 of the SARS-CoV-2, and incubated at 37°C for 1 hour under 5% CO2. The pre-incubated SARS-CoV-2 was then added to 100-µL of the VERO cells (4 X105 cells/ml) in duplicate and incubated for 10 days at 37°C under 5% CO2. Following the incubation, an inverted optical microscope was used to enumerate the formation of cytopathic effect (CPE) in wells. The highest serum dilution that protected more than 50% of the cells from CPE was taken as the neutralization titer (1).

Human ACE2 Protein (hACE-2) Binding Assay

For the ACE2 binding assay, the human ACE2 protein (hACE-2) (Native Antigen UK) was coated overnight and blocked for 3 hrs with 0.1% BSA. Then, His tag RBD in different concentrations was added to human saliva with 1/100 dilution, and the samples were incubated for 2 hrs at 37°C. After incubation, centrifuged 6000 RPM and supernatant were removed from the tube, placed into a 96-well ELISA plate, and incubated for 2 hrs at 37°C. After that, the blocking buffer was removed and washed with 200 µL 1X washing buffer once. In the next step, 100 µL of 1500X HRP-conjugated Anti-His tag antibodies were added to the 96-well plates and incubated for one hr at 37°C. Finally, the secondary antibody was removed, and the 96-well plates were washed by 200 µL using PBST four times. Then, 100 µL TMB reagents were added to each well. After 15 min, 100 µL of stop solution was added to the wells, and read the absorbance (OD450) of each well with an ELISA plate reader.

Evaluation the Cellular Immune Response by CFSE proliferation assay

For the evaluation of cell proliferative ability of peripheral mononuclear cell (MNCs), cytokine production, and surface marker expression, cell suspensions of peripheral blood from participants were prepared from recently sample, and were cultured within medium containing 10-ml of RPMI medium (Gibco, USA) with 5% fetal bovine serum for 5 hours at 37°C. Briefly, the cells were cultured within RPMI medium (Gibco, USA), containing penicillin/streptomycin 1%, and 10% FBS. The proliferative ability of MNCs of the peripheral blood from human at day 35, was measured via Carboxy fluorescein succinimidyl ester (CFSE) (BioLegend, USA) proliferation assay, using a flow cytometer (BD FACS Lyric, USA) (2, 3). In brief, CFSE labelling was performed on separated MNCs, as the tube containing the mixture of cells (1×10^6 cells in 1-ml PBS/ 2% FBS) and 1-µL of 5 mM CFSE (the final concentration of 1-µg/ml) were rapidly inverted and vortexed for 10 seconds. After 15 minutes of incubation in 37ºC in dark and two times washing, the CFSE stained MNCs were seeded in 96-well plates (1×105 cells in 100 µl of RPMI with 10% FBS). The cells were stimulated either with 5-µl of PHA (GIBCO, USA), S1 SARS-COV-2 protein (0·3µg/ml) and the heat-inactivated SARS-COV-2 viruses were obtained from convalescent patients. Then incubated in 37ºC and 5% CO2 for 72 hours until being analyzed using a flow cytometer (BD FACS Lyric, USA) and FACSuite V1·2·1 software.

Evaluation the cell markers and intracellular IFN-γ of MNCs in vaccinated human

The MNCs collected from human peripheral blood cultured as described above. After 48 hours of incubation with PHA, S1 proteins (0·3 µg/ml), and heat-inactivated SARS-COV-2 viruses, the cells were analyzed for cell surface markers and intracellular IFN-γ cytokine. Cell surface staining was performed with APC-Cy7 conjugated anti-CD3 (BD, 560590), PerCP-Cy5·5 anti-CD4 (BD, 550954), APC-R700 anti-CD8 (BD, 561093), and phycoerythrin-Cy 7 (PE- Cy7) anti CD27 (BD Pharmingen, 555275), APC anti-CD62L (BD, 104505), and Alexa flour 488 (Ax-488) anti-CD127. Therefore, cells at the density of 10^6/100µL in staining buffer were incubated with desired conjugated antibodies for 30 minutes at 4°C in the dark. Cells were then washed twice using PBS/2% FBS and centrifuged at 500-g for 5 minutes. Thereafter, for intracellular IFN-γ cytokine staining, Golgi Plug (BD, 2301kz) treated cells fixed with 250-µl of paraformaldehyde (PFA) (BD Cell fix) for 20 minutes at RT. The cells permeabilized with 0·2% tween 20/ PBS for 15 minutes at RT. Cells were stained with PE-anti IFN-γ antibody (BD Pharmingen, 555275) in 100-µL of 0·1% tween 20 in PBS/FBS 2% (perm/wash buffer) for 30 minutes at 4°C, then washed twice with perm/wash buffer and data acquisition were performed by BD FACS Lyric flow cytometer and analyzed by analyzed by FACSuite V 1·2·1 software.

Evaluation of the cytokine levels by ELISA

The level of the cytokines TNF-α, IFN-γ, IL-2, IL4, IL-6, IL-17 in the supernatants of MNCs cultured specifically stimulated with S1 proteins (0•3 µg/ml), and heat-inactivated SARS-COV-2 viruses were measured by commercial ELISA kits (R&D System, USA) according to the manufacturer's instructions. The OD value (450 nm) was read by Cytation 5 imaging reader (BioTek, USA). The results are expressed as pg/ml.

Classification of toxicity

FDA toxicity scoring is used for the severity classification (Table S1, Table S2 and Table S3).

Table S1 FDA toxicity grading scale for vital sign abnormalities·

Vital Signs *	Mild (Grade 1)	Moderate (Grade 2)	Severe (Grade 3)	Potentially Life Threatening (Grade 4)
Fever (°C) **	38·0 – 38·4	38·5 – 38·9	39·0 – 40	> 40

Tachycardia - beats per minute	101 – 115	116 – 130	> 130	ER visit or hospitalization for arrhythmia
Bradycardia - beats per minute***	50 – 54	45 – 49	< 45	ER visit or hospitalization for arrhythmia
Hypertension (systolic) - mm Hg	141 – 150	151 – 155	> 155	ER visit or hospitalization for malignant hyprtension
Hypertension (diastolic) - mm Hg	91 – 95	96 – 100	> 100	ER visit or hospitalization for malignant hypertension
Hypotension (systolic) – mm Hg	85 – 89	80 – 84	< 80	ER visit or hospitalization for hypotensive shock
Respiratory Rate – breaths per minute	17 – 20	21 – 25	> 25	Intubation

* Subject should be at rest for all vital sign measurements· ** Oral temperature; no recent hot or cold beverages or smoking· *** When resting heart rate is between 60 – 100 beats per minute· Use clinical judgement when characterizing bradycardia among some healthy subject populations, for example, conditioned athletes·

Table S2 FDA toxicity grading scales for solicited local and systemic adverse events

**Local Reaction to Injectable Product**	**Mild (Grade 1)**	**Moderate(Grade 2)**	**Severe (Grade 3)**	**Potentially Life Threatening (Grade 4)**
Pain	Does not interfere with activity	Repeated use of non-narcotic pain reliever > 24 hours or interferes with activity	Any use of narcotic pain reliever or prevents daily activity	Emergency room (ER) visit or hospitalization
Tenderness	Mild discomfort to touch	Discomfort with movement	Significant discomfort at rest	ER visit or hospitalization
Erythema/Redness *	2·5 – 5 cm	5·1 – 10 cm	> 10 cm	Necrosis or exfoliative dermatitis
Induration/Swelling **	2·5 – 5 cm and does not interfere with activity	5·1 – 10 cm or interferes with activity	> 10 cm or prevents daily activity	Necrosis
Nausea/vomiting	No interference with activity or 1 – 2 episodes/24 hours	Some interference with activity or > 2 episodes/24 hours	Prevents daily activity, requires outpatient IV hydration	ER visit or hospitalization for hypotensive shock
Diarrhea	2 – 3 loose stools or < 400 gms/24 hours	4 – 5 stools or 400 – 800 gms/24 hours	6 or more watery stools or > 800 gms/24 hours or requires outpatient IV hydration	ER visit or hospitalization
Headache	No interference with activity	Repeated use of non-narcotic pain reliever > 24 hours or some interference with activity	Significant; any use of narcotic pain reliever or prevents daily activity	ER visit or hospitalization
Fatigue	No interference with activity	Some interference with activity	Significant; prevents daily activity	ER visit or hospitalization
Myalgia	No interference with activity	Some interference with activity	Significant; prevents daily activity	ER visit or hospitalization
Illness or clinical adverse event (as defined according to applicable regulations)	No interference with activity	Some interference with activity not requiring medical intervention	Prevents daily activity and requires medical intervention	ER visit or hospitalization

* In addition to grading the measured local reaction at the greatest single diameter, the measurement should be recorded as a continuous variable· ** Induration/Swelling should be evaluated and graded using the functional scale as well as the actual measurement·

Table S3 FDA toxicity grading scales for clinical laboratory abnormalities

**Serum ***	**Attributable to lab error/pop norms**	**Mild (Grade 1)**	**Moderate (Grade 2)**	**Severe (Grade 3)**	**Potentially Life Threatening (Grade 4)****
Sodium – Hyponatremia mEq/L	136-145	132 – 135	130 – 131	125 – 129	< 125
Sodium – Hypernatremia mEq/L	136-145	146 – 147	148– 149	150 – 151	> 151

Potassium – Hyperkalemia mEq/L	3·7- 5·5	5·6 – 5·7	5·8 – 5·9	6 – 6·1	> 6·1
Potassium – Hypokalemia mEq/L		3·5 – 3·6	3·3 – 3·4	3·1 – 3·2	< 3·1
Glucose – Hypoglycemia mg/dL		65 – 69	55 – 64	45 – 54	< 45
Glucose – Hyperglycemia<br>Fasting – mg/dL<br>Random – mg/dL		100 – 110<br>110 – 125	111 – 125<br>126 – 200	>125<br>>200	Insulin requirements or hyperosmolar coma
Blood Urea Nitrogen BUN mg/dL		23 – 26	27 – 31	> 31	Requires dialysis
Creatinine – mg/dL		1·5 – 1·7	1·8 – 2·0	2·1 – 2·5	> 2·5 or requires dialysis
Calcium – hypocalcemia mg/dL		8·0 – 8·4	7·5 – 7·9	7·0 – 7·4	< 7·0
Calcium – hypercalcemia mg/dL		10·5 – 11·0	11·1 – 11·5	11·6 – 12·0	> 12·0
Magnesium – hypomagnesemia mg/dL		1·3 – 1·5	1·1 – 1·2	0·9 – 1·0	< 0·9
Phosphorous – hypophosphatemia mg/dL		2·3 – 2·5	2·0 – 2·2	1·6 – 1·9	< 1·6
CPK – mg/dL		1·25 – 1·5 x ULN***	1·6 – 3·0 x ULN	3·1 –10 x ULN	> 10 x ULN
Albumin – Hypoalbuminemia g/dL		2·8 – 3·1	2·5 – 2·7	< 2·5	--
Total Protein – Hypoproteinemia g/dL		5·5 – 6·0	5·0 – 5·4	< 5·0	--
Alkaline phosphate – increase by factor		1·1 – 2·0 x ULN	2·1 – 3·0 x ULN	3·1 – 10 x ULN	> 10 x ULN
Liver Function Tests –ALT, AST<br>increase by factor		1·5 – 2·5 x ULN	2·6 – 5·0 x ULN	5·1 – 10 x ULN	> 10 x ULN
Bilirubin – when accompanied by any increase in Liver Function Test<br>increase by factor		1·5 – 1·75x ULN	1·76– 2x ULN	2·1– 2·25x ULN	> 2·25x ULN
Bilirubin – when Liver Function Test is normal; increase by factor		1·5 – 2x ULN	2·1 – 2·5x ULN	2·6 – 3·0 x ULN	> 3·0 x ULN
Cholesterol		201 – 210	211 – 225	> 226	---
Pancreatic enzymes – amylase, lipase		1·1 – 1·5 x ULN	1·6 – 2·0 x ULN	2·1 – 5·0 x ULN	> 5·0 x ULN
Hemoglobin (Female) - gm/dL	11-12	10·5 - 0·9	9·5 – 10·4	8·0 – 9·4	< 8·0
Hemoglobin (Female) change from baseline value - gm/dL		Any decrease – 1·5	1·6 – 2·0	2·1 – 5·0	> 5·0
Hemoglobin (Male) - gm/dL		12·5 – 13·5	10·5 – 12·4	8·5 – 10·4	< 8·5
Hemoglobin (Male) change from baseline value – gm/dL		Any decrease – 1·5	1·6 – 2·0	2·1 – 5·0	> 5·0
WBC Increase - cell/mm^3		10,800 – 15,000	15,001 – 20,000	20,001 – 25, 000	> 25,000
WBC Decrease - cell/mm^3		2,500 – 3,500	1,500 – 2,499	1,000 – 1,499	< 1,000
Lymphocytes Decrease - cell/mm^3		750 – 1,000	500 – 749	250 – 499	< 250
Neutrophils Decrease - cell/mm^3		1,500 – 2,000	1,000 – 1,499	500 – 999	< 500
Eosinophils - cell/mm^3		650 – 1500	1501 - 5000	> 5000	Hypereosinophilic
Platelets Decreased - cell/mm^3		125,000 – 140,000	100,000 – 124,000	25,000 – 99,000	< 25,000
PT – increase by factor (prothrombin time)		1·0 – 1·10 x ULN**	1·11 – 1·20 x ULN	1·21 – 1·25 x ULN	> 1·25 ULN

PTT – increase by factor (partial thromboplastin time)		1·0 – 1·2 x ULN	1·21 – 1·4 x ULN	1·41 – 1·5 x ULN	> 1·5 x ULN
Fibrinogen increase - mg/dL		400 – 500	501 – 600	> 600	--
Fibrinogen decrease - mg/dL		150 – 200	125 – 149	100 – 124	< 100 or associated with gross bleeding or (DIC)
Urine protein	-	Trace	1+	2+	Hospitalization or dialysis
Urine glucose	-	Trace	1+	2+	Hospitalization for hyperglycemia
Blood (microscopic) – red blood cells per high power field (rbc/hpf)	1-5	6 - 10	11 – 50	> 50 and/or gross blood	Hospitalization or packed red blood cells (PRBC) transfusion

* The laboratory values provided in the tables serve as guidelines and are dependent upon institutional normal parameters. Institutional normal reference ranges should be provided to demonstrate that they are appropriate. ** The clinical signs or symptoms associated with laboratory abnormalities might result in characterization of the laboratory abnormalities as Potentially Life Threatening (Grade 4). For example, a low sodium value that falls within a grade 3 parameter (125-129 mE/L) should be recorded as a grade 4 hyponatremia event if the subject had a new seizure associated with the low sodium value. ***ULN" is the upper limit of the normal range.

Table S4: World Health Organisation-Uppsala Monitoring Centre causality assessment scale

Causality term	Assessment criteria
Certain	1. Event or laboratory test abnormality, with plausible time relationship to drug intake<br>2. Cannot be explained by disease or other drugs<br>3. Response to withdrawal Plausible (pharmacologically, pathologically)<br>4. Event definitive pharmacologically or phenomenologically (i.e., an objective and specific medical disorder or a recognized pharmacological phenomenon)<br>5. Rechallenge satisfactory, if necessary
Probable or likely	1. Event or laboratory test abnormality, with reasonable time relationship to drug intake<br>2. Unlikely to be attributed to disease or other drugs<br>3. Response to withdrawal clinically reasonable<br>4. Rechallenge not required
Possible	1. Event or laboratory test abnormality, with reasonable time relationship to drug intake<br>2. Could also be explained by disease or other drugs<br>3. Information on drug withdrawal may be lacking or unclear
Unlikely	1. Event or laboratory test abnormality, with a time to drug intake that makes a relationship improbable (but not impossible)<br>2. Disease or other drugs provide plausible explanations
Conditional or unclassified	1. Event or laboratory test abnormality or<br>2. More data for proper assessment needed, additional data under examination
Unassessable/ unclassifiable	1. Report suggesting an adverse reaction<br>2. Cannot be judged because information is insufficient or contradictory<br>3. Data cannot be supplemented for verified

Table S5: Predefined time schedule of the study.

	screening	0	7	14	21	28	35	51	58	65	150
Nasal swab for COVID-19 PCR test[a]	×										
Visit to the study center and physical examination	×	×	×	×	×	×	×	×	×	×	×
Psychological assessment	×										
Blood sample: screening [b]	×										
Vaccination		×			×			×			
Blood sample: safety [c]			×			×			×		
Blood sample: humoral immunogenicity		×	×	×	×	×	×			×	×
Blood sample: cellular immunogenicity		×					×			×	×
Blood sample: VNT		×					×			×	×
Immediate and solicited local and systemic reactions		←	→		←	→		←	→		
Unsolicited adverse events		←	—	—	—	—	—	—	—	—	→
Medically attended adverse events		←	—	—	—	—	—	—	—	—	→

[a] Participants underwent nasopharyngeal swab testing for SARS-CoV-2 whenever they reported symptoms suggestive of possible infection.
[b] Tests included CBC, ESR, CRP, Sodium, potassium, Magnesium, phosphorous, Albumin, Total protein, BUN, Creatinine, PT, PTT, Alkaline phosphatase, ALT, AST, total bilirubin, LDH, urine protein, urine glucose, U/A RBC, HbA1c, IgM and IgG for SARS-COV-2, HBsAg, HBcAb, Anti HCVAb, HIV, and Beta HCG for women.
[c] Tests included CBC, ESR, CRP, Alkaline phosphatase, ALT, AST, total bilirubin, LDH, urine protein, urine glucose, and U/A RBC.

II-Supplemental Study Results

Demographic characteristic

Table S6 Demographic characteristics of the sentinel participants

Variables	Placebo	Vac. 5 µ	Vac. 10 µ	Vac. 20 µ	Total
	n=1	n=4	n=4	n=4	n=13
Gender, n(%)					
Male	1 (100·0)	3 (75·0)	3 (75·0)	3 (75·0)	10 (76·92)
Female	0 (0·00)	1 (25·0)	1 (25·0)	1 (25·0)	3 (23·08)
Age					
Mean (SD)	36	28·3 (7·1)	41·8 (6·9)	45·5 (8·2)	38·3 (9·8)
Median (min – max)	-	26·5 (22 – 38)	40 (36 – 51)	44 (38 – 55)	38·3 (22 – 55)
Body-mass index					
Mean (SD)	28·1	24·1 (3·3)	25·6 (2·6)	24·7 (2·9)	25·0 (2·8)
Median (min - max)	-	23·8 (20·3 28·4)	25·5 (22·7-28·7)	25·1 (21·3-27·5)	24·6 (20·3-28·7)
Smoking, n(%)					
Current Smoking	0 (0·0)	1 (25·0)	0 (0·0)	0 (0·0)	1
Never Smoking	1 (100)	3 (75·0)	4 (100)	4 (100)	12
Education, n(%)					
Diploma	0	2	1	0	3
Diploma plus	1	0	0	0	1
Bachelor	0	1	0	2	3
Doctoral and above	0	1	3	2	6
Job, n (%)					
Unemployed/Retired	0	2	0	1	3
Government employee	1	1	3	2	7
Private employee	0	1	1	0	2
Housewife	0	0	0	1	1

Table S7 Geometric mean ratio and 95% CI of specific antibody responses (AUC) to S, S1, S2, RBD and NTD antigens (Variant in Wuhan) in the intervention groups over the predefined study time schedule.

		Baseline	Day 7	Day 14	Day 21	Day 28	Day 35	Day 65	Day 150
	GMR_{AUC} (95% CI)								
Anti S antibody	Placebo	1	1	1	1	1	1	1	1
	Vac. 5 µg	1.28 (1.08-1.52)	1.36 (1.00-1.84)	1.50 (0.99-2.28)	1.50 (0.92-2.45)	1.96 (1.03-3.73)	3.44 (1.66-7.14)	3.55 (1.69-11.25)	3.20 (1.70-9.03)
	Vac. 10 µg	1.01 (0.85-1.20)	1.21 (0.90-1.64)	1.29 (0.85-1.96)	1.57 (0.96-2.57)	1.97 (1.03-3.78)	4.19 (2.00-8.79)	3.68 (1.61-15.33)	5.60 (1.68-27.39)
	Vac. 20 µg	0.90 (0.75-1.07)	0.93 (0.69-1.26)	1.03 (0.68-1.56)	1.15 (0.70-1.89)	1.96 (1.02-3.78)	6.12 (2.90-12.89)	5.15 (1.66-25.79)	5.61 (1.70-25.28)
Anti S1 antibody	Placebo	1	1	1	1	1	1	1	1
	Vac. 5 µg	1.28 (1.08-1.52)	1.36 (1.0-1.84)	1.50 (0.99-2.28)	1.50 (0.92-2.45)	1.96 (1.03-3.73)	3.46 (1.67-7.18)	2.70 (0.73-9.97)	3.04 (0.87-10.59)
	Vac. 10 µg	1.01 (1.08-1.20)	1.21 (1.14-1.64)	1.24 (0.82-1.88)	1.57 (0.96-2.57)	1.97 (1.03-3.78)	4.22 (2.01-8.84)	2.87 (0.87-9.52)	5.61 (1.64-19.17)
	Vac. 20 µg	0.90 (0.75 -1.07)	0.93 (0.69-1.26)	1.03 (0.68-1.56)	1.15 (0.70-1.89)	1.96 (1.02-3.78)	6.15 (2.92-12.97)	4.20 (1.19-14.84)	4.95 (1.40-17.45)
Anti S2 antibody	Placebo	1	1	1	1	1	1	1	1
	Vac. 5 µg	1.46 (1.09-1.95)	1.49 (1.05-2.10)	1.65 (1.05-2.61)	1.73 (1.02-2.93)	2.17 (1.10-4.30)	4.04 (1.90-8.59)	2.69 (1.67-6.82)	3.17 (1.62-10.91)
	Vac. 10 µg	1.07 (0.80-1.44)	1.26 (0.89-1.77)	1.39 (0.88-2.20)	1.67 (0.99-2.84)	2.17 (1.09-4.34)	4.89 (2.28-10.50)	2.94 (1.60-9.78)	4.40 (1.61-22.65)
	Vac. 20 µg	0.87 (0.65-1.16)	0.95 (0.67-1.34)	1.07 (0.68-1.68)	1.25 (0.73-2.12)	2.14 (1.06-4.30)	6.98 (3.23-15.08)	4.05 (1.64-16.61)	4.68 (1.63-23.81)
Anti RBD antibody	Placebo	1	1	1	1	1	1	1	1
	Vac. 5 µg	1.38 (1.13-1.68)	1.45 (1.06-1.98)	1.57 (1.00-2.47)	1.43 (0.87-2.34)	1.99 (1.06-3.76)	3.40 (1.63-7.07)	2.69 (1.68-6.82)	3.38 (1.68-10.38)
	Vac. 10 µg	1.05 (0.86-1.27)	1.21 (0.89-1.64)	1.30 (0.83-2.05)	1.57 (0.96-2.57)	1.88 (0.99-3.58)	4.07 (1.94-8.55)	3.04 (1.60-10.49)	5.98 (1.67-32.79)
	Vac. 20 µg	0.91 (0.75-1.11)	1.01 (0.74-1.37)	1.05 (0.67-1.65)	1.13 (0.69-1.86)	2.01 (1.05-3.84)	5.88 (2.78-12.44)	4.22 (1.65-17.99)	5.75 (1.69-27.94)
Anti NTD antibody	Placebo	1	1	1	1	1	1	1	1
	Vac. 5 µg	1.31 (1.08-1.60)	1.40 (1.03-1.91)	1.54 (1.01-2.37)	1.50 (0.91-2.47)	1.93 (1.01-3.67)	3.24 (1.51-6.93)	2.30 (1.71-4.71)	3.44 (1.68-10.70)
	Vac. 10 µg	1.06 (0.87-1.29)	1.21 (0.89-1.64)	1.31 (0.85-2.00)	1.62 (0.98-2.66)	1.98 (1.03-3.81)	4.23 (1.95-9.15)	3.01 (1.63-9.49)	5.75 (1.67-30.27)
	Vac. 20 µg	0.93 (0.76-1.13)	1.01 (0.74-1.37)	1.12 (0.73-1.71)	1.24 (0.75-2.05)	2.11 (1.09-4.09)	6.67 (3.07-14.52)	4.12 (1.68-15.49)	5.52 (1.69-25.79)

Table S8 Serum levels of specific antibodies against S, S1, S2, RBD, NTD and N antigens (Variant in Wuhan) in trial participants at enrollment.

	**Placebo**	**Vac. 5 µ**	**Vac. 10 µ**	**Vac. 20 µ**	**Total**
	n=30	n=30	n=30	n=30	n=120
Antibody for S Ag (AUC)	19634 (17535-21986)	19583 (17932-21386)	17892 (16181-19784)	17002 (15407-18762)	19472 (18468-20532)
Antibody for S1 Ag (AUC)	20835 (18594-23345)	26027 (23809-28450)	20241 (18075-22666)	17868 (16092-19841)	21859 (20689-23096)
Antibody for S2 Ag (AUC)	16890 (13939-20465)	23729 (20450-27534)	17019 (13825-20951)	13521 (11400-16035)	18494 (16885-20256)
Antibody for RBD Ag (AUC)	20683 (17967-23809)	27496 (24320-31087)	20435 (18014-23181)	17721 (16150-19445)	22167 (20809-23613)
Antibody for NTC Ag (AUC)	17658 (15758-19788)	22123 (20394-23999)	17472 (15462-19744)	15474 (13972-17138)	19181 (18050 – 20384)
Antibody for N Ag (AUC)	17835 (15768-20173)	23755 (21993-25659)	18798 (17133-20625)	16692 (14833-18783)	19156 (18135 – 20236)

Table S9 Number and percentages of subjects experiencing solicited local and systemic adverse events vaccination dose, by FDA toxicity grade

	**Dose 1**				**Dose 2**				**Any adverse event by person**			
Symptom	**Placebo** n=31	**Vac. 5 µ** n=34	**Vac. 10 µ** n=34	**Vac. 20 µ** n=34	**Placebo** n=29	**Vac. 5 µ** n=34	**Vac. 10 µ** n=33	**Vac. 20 µ** n=32	**Placebo** n=31	**Vac. 5 µ** n=34	**Vac. 10 µ** n=34	**Vac. 20 µ** n=34
**Local**												
Pain												
Grade 1	4 (12·9 %)	5 (14·7 %)	1 (2·9 %)	3 (8·8 %)	5 (17·2%)	7 (20·6%)	3 (9·1%)	7 (21·8%)	7 (22·6%)	10(29·4%)	3 (8·8%)	6 (17·6%)
Grade 2	0 (0·0%)	0 (0·0%)	0 (0·0%)	1 (2·9 %)	1 (3·4%)	0 (0·0%)	0 (0·0%)	1 (3·1%)	1 (3·2%)	0 (0·0%)	0 (0·0%)	2 (5·9%)
Tenderness												
Grade 1	6 (19·3 %)	6 (17·6 %)	3 (29·4 %)	7 (20·6 %)	4 (13·8%)	7 (20·6%)	9 (27·3%)	6 (18·7%)	7 (22·6%)	9 (26·5%)	7 (20·6%)	9 (26·5%)
Grade 2	3 (9·7 %)	3 (8·8 %)	4 (11·7 %)	3 (8·8%)	6 (20·6%)	8 (23·5%)	4 (12·1%)	7 (21·8%)	7 (22·6%)	9 (26·5%)	8 (23·5%)	8 (23·5%)
Itching												
Grade 1	0 (0·0%)	1 (2·9 %)	1 (2·9 %)	0 (0·0%)	0 (0·0%)	0 (0·0%)	0 (0·0%)	1 (3·1%)	0 (0·0%)	1 (2·9%)	1 (2·9%)	1 (2·9%)
Grade 2	0 (0·0%)	0 (0·0%)	0 (0·0%)	0 (0·0%)	0 (0·0%)	0 (0·0%)	0 (0·0%)	0 (0·0%)	0 (0·0%)	0 (0·0%)	0 (0·0%)	0 (0·0%)
Redness												
Grade 1	0 (0·0%)	0 (0·0%)	0 (0·0%)	0 (0·0%)	1 (3·4%)	1 (2·9%)	0 (0·0%)	0 (0·0%)	1 (3·2%)	1 (2·9%)	0 (0·0%)	0 (0·0%)
Grade 2	0 (0·0%)	0 (0·0%)	0 (0·0%)	0 (0·0%)	0 (0·0%)	0 (0·0%)	0 (0·0%)	0 (0·0%)	0 (0·0%)	0 (0·0%)	0 (0·0%)	0 (0·0%)
Swelling												
Grade 1	0 (0·0%)	0 (0·0%)	0 (0·0%)	0 (0·0%)	0 (0·0%)	0 (0·0%)	0 (0·0%)	0 (0·0%)	0 (0·0%)	0 (0·0%)	0 (0·0%)	0 (0·0%)
Grade 2	0 (0·0%)	0 (0·0%)	0 (0·0%)	0 (0·0%)	0 (0·0%)	0 (0·0%)	0 (0·0%)	0 (0·0%)	0 (0·0%)	0 (0·0%)	0 (0·0%)	0 (0·0%)
Any solicited local												
Grade 1	7 (22·6 %)	10 (29·4 %)	4 (11·7 %)	8 (23·5 %)	4 (13·8%)	8 (23·5%)	9 (27·3%)	7 (21·8%)	7 (22·6%)	10(29·4%)	7 (20·6%)	10(29·4%)
Grade 2	3 (9·7 %)	3 (8·8 %)	4 (11·7 %)	4 (11·7 %)	6 (20·6%)	8 (23·5%)	4 (12·1%)	7 (21·8%)	7 (22·6%)	9 (26·5%)	8 (23·5%)	8 (23·5%)
**Systemic**												
Nausea												
Grade 1	1 (3·2%)	0 (0·0%)	1 (2·9%)	0 (0·0%)	0 (0·0%)	0 (0·0%)	0 (0·0%)	0 (0·0%)	1 (3·2%)	0 (0·0%)	1 (2·9%)	0 (0·0%)
Grade 2	0 (0·0%)	0 (0·0%)	0 (0·0%)	0 (0·0%)	0 (0·0%)	0 (0·0%)	0 (0·0%)	0 (0·0%)	0 (0·0%)	0 (0·0%)	0 (0·0%)	0 (0·0%)
Diarrhea												
Grade 1	2 (6·5%)	2 (5·9%)	0 (0·0%)	1 (2·9%)	1 (3·4%)	0 (0·0%)	1 (3·0%)	1 (3·1%)	3 (9·7%)	2 (5·9%)	1 (2·9 %)	2 (5·9%)
Grade 2	0 (0·0%)	0 (0·0%)	1 (2·9%)	0 (0·0%)	0 (0·0%)	0 (0·0%)	0 (0·0%)	0 (0·0%)	0 (0·0%)	0 (0·0%)	1 (2·9%)	0 (0·0%)
Headache												
Grade 1	4 (12·9%)	1 (2·9%)	4 (11·8%)	5 (14·7%)	5 (17·2%)	2 (5·9%)	6 (18·2%)	4 (11·7%)	7 (22·6%)	3 (8·8%)	9 (2·9%)	6 (17·6%)
Grade 2	1 (3·2%)	6 (17·6%)	0 (0·0%)	3 (8·8%)	0 (0·0%)	0 (0·0%)	0 (0·0%)	3 (8·8%)	1 (3·2%)	6 (17·6%)	0 (0·0%)	6 (17·6%)
Fatigue												
Grade 1	0 (0·0%)	1 (2·9%)	1 (2·9%)	5 (14·7%)	5 (17·2%)	3 (8·8%)	1 (3·0%)	3 (8·8%)	5 (0·0%)	4 (11·7%)	2 (5·9%)	7 (20·6%)
Grade 2	1 (3·2%)	0 (0·0%)	0 (0·0%)	0 (0·0%)	0 (0·0%)	0 (0·0%)	1 (3·0%)	1 (3·1%)	1 (16·1%)	0 (0·0%)	1 (2·9%)	1 (2·9%)
Myalgia												
Grade 1	1 (3·2%)	0 (0·0%)	2 (5·9%)	3 (8·8%)	3 (10·3%)	1 (2·9%)	1 (3·0%)	1 (3·1%)	4 (12·9%)	1 (2·9%)	3 (8·8%)	4 (11·7%)
Grade 2	0 (0·0%)	1 (2·9%)	0 (0·0%)	0 (0·0%)	0 (0·0%)	0 (0·0%)	0 (0·0%)	2 (6·2%)	0 (0·0%)	1 (2·9%)	0 (0·0%)	2 (5·9%)
Any systemic												
Grade 1	9 (29·0%)	5 (14·7%)	6 (17·6%)	7 (20·6%)	8 (27·5%)	5 (14·7%)	8 (24·2%)	6 (18·7%)	10(32·2%)	8 (23·5%)	10(29·4%)	9 (26·5%)
Grade 2	1 (3·2%)	7 (20·6%)	1 (2·9%)	3 (8·8%)	0 (0·0%)	0 (0·0%)	1 (3·0%)	3 (9·4%)	1 (3·2%)	7 (20·6%)	2 (5·9%)	6 (17·5%)

Table S10 Number and percentages of subjects experiencing solicited systemic adverse events after third dose of vaccine, by FDA toxicity grade

	Placebo	Vac. 5 µ	Vac. 10 µ	Vac. 20 µ
	n=27	n=34	n=32	n=30
Nausea				
Grade 1	0 (0.0%)	2 (5·8%)	1 (3·1%)	0 (0·0%)
Grade 2	0 (0·0%)	0 (0·0%)	0 (0·0%)	0 (0·0%)
Diarrhea				
Grade 1	0 (0·0%)	0 (0·0%)	1 (3·1 %)	0 (0·0%)
Grade 2	0 (0·0%)	0 (0·0%)	0 (0·0%)	1 (3·3%)
Headache				
Grade 1	1 (3·7%)	3 (8·8%)	1 (3·1%)	0 (0·0%)
Grade 2	0 (0·0%)	0 (0·0%)	0 (0·0%)	0 (0·0%)
Fatigue				
Grade 1	0 (0·0%)	1 (2·9%)	0 (0·0%)	0 (0·0%)
Grade 2	1 (3·7%)	0 (0·0%)	0 (0·0%)	1 (3·3%)
Myalgia				
Grade 1	0 (0·0%)	1 (2·9%)	1 (3·1%)	0 (0·0%)
Grade 2	0 (0·0%)	0 (0·0%)	0 (0·0%)	0 (0·0%)
Any				
Grade 1	1 (3·7%)	5 (14·7%)	3 (9·4%)	0 (0·0%)
Grade 2	1 (3·7%)	0 (0·0%)	0 (0·0%)	1 (3·3%)

Table S11 List of adverse events, their grades and causal relationship with the intervention received (sorted by grade) during the six-month follow-up period.

Randomization code	Adverse Event	Grade	Causal Relationship	Group of IMP
002-5997	Chilling	1	Unlikely	B
015-6152	Hordeolum	2	Unlikely	A
049-4249	Oral Aphthus	1	Unlikely	B
084-3861	Tension Headache	2	Unlikely	D
111-5644	Unspecific Chest Discomfort	2	Unlikely	A
124-9095	Fatigue	1	Unlikely	D
011-4098	Migraine Attack	2	Suspicious	D
011-4098	Migraine Attack	2	Suspicious	D
014-2636	Cold Sore	2	Suspicious	A
022-7475	Irregular Menstruation	2	Suspicious	A
030-3789	Fatigue	1	Suspicious	A
035-2442	Dizziness	2	Suspicious	C
035-2442	Migraine Attack	3	Suspicious	C
055-2443	Mastalgia	1	Suspicious	A
065-4258	Dizziness	2	Suspicious	B
065-4258	Migraine Attack	2	Suspicious	B
068-8263	Conjunctivitis	1	Suspicious	D
068-8263	Conjunctivitis	1	Suspicious	D
069-6168	Migraine Attack	3	Suspicious	D
080-7939	Pruritus	2	Suspicious	B
080-7939	Pruritus	2	Suspicious	B
085-3718	Pruritus	1	Suspicious	D
085-3718	Skin Rash With Pruritus	1	Suspicious	D
088-9674	Conjunctivitis	1	Suspicious	B
091-5325	Eosinophilia	2	Suspicious	C
093-3815	Skin Rash Without Pruritus	1	Suspicious	D
098-6926	Vestibulitis	3	Suspicious	A
100-6626	Pruritus	2	Suspicious	D
114-6141	Conjunctivitis	2	Suspicious	C
115-9342	Dizziness	2	Suspicious	D
131-3597	Myalgia	1	Suspicious	D
002-5998	Cellulitis	3	Probable	B
008-9952	Skin Papule	2	Probable	C
053-9290	Urticaria	3	Probable	D
064-8313	Skin Papule	2	Probable	B
071-7994	Pruritus	1	Probable	A
072-1431	Pruritus	1	Probable	B
074-2212	Skin Papule	2	Probable	C
123-7629	Lat. Cutaneous Nerve Injury	3	Probable	D
014-2636	Panic Attack	N/A	Not Related	A
017-6601	Gastroenteritis	1	Not Related	B
017-6601	Transient Numbness In Limbs	1	Not Related	B
021-9147	Nephrolithiasis	3	Not Related	B
024-7623	Unspecific Neck Pain	1	Not Related	B
027-6693	Abnl Lung Sound Due To Copd	1	Not Related	B
033-1576	Burning Of The Oral Mucosa	1	Not Related	B
033-1576	Appendectomy Scar Discharge	1	Not Related	B
036-1133	Transient Knee Pain	2	Not Related	C
038-4440	Vaginitis	2	Not Related	A
041-2213	Constipation	2	Not Related	B

044-5350	Unspecific Chest Pain	2	Not Related	C
046-9997	Headache	2	Not Related	A
049-4249	Unspecific Epigastria Pain	1	Not Related	B
049-4249	Transient Unspecific Epigastria Pain	1	Not Related	B
054-8016	Flank Pain	1	Not Related	A
057-9981	Transient Unspecific Epigastria Pain, peptic disease history	2	Not Related	B
058-4898	Gastritis (With Previous History)	2	Not Related	C
059-4435	Post Nasal Discharge	1	Not Related	C
062-3195	Skin Papule	1	Not Related	A
064-8313	Caught	N/A	Not Related	B
066-9582	Gastroenteritis	1	Not Related	C
071-7994	Alopecia Areata	N/A	Not Related	A
072-1431	Fatigue	1	Not Related	B
075-8826	Sport Knee Injury	N/A	Not Related	C
078-2710	Transient Muscular Spasm	1	Not Related	A
080-7939	Unspecific Chest Pain	2	Not Related	B
080-7939	Transient Numbness In Limbs	1	Not Related	B
080-7939	Cystitis	2	Not Related	B
080-7939	Pelvic Pain, Ovarian Cysts	2	Not Related	B
080-7939	Dyspepsia	1	Not Related	B
081-4202	Allergic Rhinitis	1	Not Related	B
083-9391	Unspecific Chest Pain	1	Not Related	C
084-3861	Headache	3	Not Related	D
085-3718	Pruritus	1	Not Related	D
086-8281	Nephrolithiasis	3	Not Related	A
089-3041	Radius Bone Fracture	4	Not Related	B
090-4618	Pharyngitis	N/A	Not Related	C
091-5325	Acute Sinusitis	N/A	Not Related	C
103-6915	Headache	2	Not Related	A
103-6915	Dizziness	2	Not Related	A
113-3478	Nose Trauma	N/A	Not Related	C
114-6141	Transient Numbness In Injected Limb	1	Not Related	C
118-5894	Transient Anosmia	1	Not Related	A
122-8924	Palpitation	2	Not Related	C
126-2641	Dysmenorrhea	1	Not Related	A
127-8883	Sun Burning	2	Not Related	A
130-6142	Dysuria	1	Not Related	D

Table S12 List of patients with positive COVID-19 PCR test result during the follow up period

Randomization code	Group	Grade
001-5076	Placebo	Mild
064-8313	Placebo	Mild
026-7568	Placebo	Mild
021-9147	Placebo	Mild
119-2963	Placebo	Mild
055-2443	Placebo	Mild
121-8571	Placebo	Moderate
089-3041	5 µg	Mild
037-5616	5 µg	Mild

018-1699	5 µg	Moderate to Severe
015-6152	5 µg	Mild
028-4429	5 µg	Moderate to Severe
025-8885	5 µg	Mild
061-7701	10 µg	Mild
067-9297	10 µg	Mild
122-8924	10 µg	Mild
100-6626	10 µg	Mild
080-7939	20 µg	Moderate
085-3718	20 µg	Mild
093-3815	20 µg	Mild
120-3855	20 µg	Mild

Table S13 Abnormal vital signs during 3 hours after dose 1 and 2

Symptom	Dose 1				Dose 2			
	Placebo n=31	Vac. 5 µ n=34	Vac. 10 µ n=34	Vac.20 µ n=34	Placebo n=29	Vac. 5 µ n=34	Vac. 10 µ n=33	Vac.20µ n=32
**Fever(°C)**								
Grade 1	0	0	0	0	0	0	0	0
Grade 2	0	0	0	0	0	0	0	0
Grade 3	0	0	0	0	0	0	0	0
Grade 4	0	0	0	0	0	0	0	0
**Tachycardia**								
Grade 1	0	2 (5.9 %)	0	0	0	1 (2.9 %)	0	0
Grade 2	0	0	0	0	0	0	0	0
Grade 3	0	0	0	0	0	0	0	0
Grade 4	0	0	0	0	0	0	0	0
**Bradycardia**								
Grade 1	0	0	0	0	0	0	0	0
Grade 2	0	0	0	0	0	0	0	0
Grade 3	0	0	0	0	0	0	0	0
Grade 4	0	0	0	0	0	0	0	0
**Hypertension (Systolic)**								
Grade 1	1 (3.2 %)	0	0	0	0	0	0	0
Grade 2	0	0	0	0	0	0	0	0
Grade 3	0	0	0	0	0	0	0	0
Grade 4	0	0	0	0	0	0	0	0
**Hypertension (diastolic)**								
Grade 1	1 (3.2 %)	0	1 (2.9 %)	0	0	0	0	0
Grade 2	0	0	1 (2.9%)	0	0	0	0	0

Grade 3	0	0	0	0	0	0	0	0
Grade 4	0	0	0	0	0	0	0	0
**Hypotension (Systolic)**								
Grade 1	0	0	0	0	0	0	0	0
Grade 2	0	0	0	0	0	0	0	0
Grade 3	0	0	0	0	0	0	0	0
Grade 4	0	0	0	0	0	0	0	0
**Respiration rate**								
Grade 1	1 (3.2 %)	4 (11.7%)	0	0	0	0	0	0
Grade 2	0	0	0	0	0	0	0	0
Grade 3	0	0	0	0	0	0	0-	0
Grade 4	0	0	0	0	0	0	0	0

Table S14 Number and percentages of subjects experiencing laboratory abnormalities by FDA toxicity grade

	Baseline				Day 7				Day 28				Day 58			
	Placebo	Vac. 5 µ	Vac. 10 µ	Vac. 20 µ	Placebo	Vac. 5 µ	Vac. 10 µ	Vac. 20 µ	Placebo	Vac. 5 µ	Vac. 10 µ	Vac. 20 µ	Placebo	Vac. 5 µ	Vac. 10 µ	Vac. 20 µ
	n=31	n=34	n=34	n=34	n=31	n=34	n=34	n=34	n=29	n=34	n=32	n=31	n=27	n=34	n=32	n=30
Hemoglobin gm/dL (decrease)																
Grade 1	-	-	2 (5·9%)	2 (5·9%)	-	-	3 (8·8%)	1 (2·9%)	-	-	2 (6·2%)	2 (6·2%)	-	-	1(3·1%)	1(3·3%)
Grade 2	-	-	-	-	-	-	-	1(2·9 %)	-	-	-	1 (3·2%)	-	-	1(3·1%)	1(3·3%)
Grade 3	-	-	-	-	-	-	-	-	-	-	-	-	-	-	-	-
WBC increase cell/mm^3																
Grade 1	-	-	-	-	-	-	-	-	-	-	-	-	-	-	-	1(3·3%)
Grade 2	-	-	-	-	-	-	-	-	-	-	-	-	-	-	-	-
Grade 3	-	-	-	-	-	-	-	-	-	-	-	-	-	-	-	-
WBC decrease cell/mm^3																
Grade 1	1 (3·2%)	-	-	-	1 (3·2%)	-	-	-	1 (3·4%)	-	-	-	-	-	-	-
Grade 2	-	-	-	-	-	-	-	-	-	-	-	-	-	-	-	-
Grade 3	-	-	-	-	-	-	-	-	-	-	-	-	-	-	-	-
Lymphocytes cell/mm^3																
Grade 1	-	-	-	-	-	-	-	-	-	-	-	-	-	-	-	-
Grade 2	-	-	-	-	-	-	-	-	-	-	-	-	-	-	-	-
Grade 3	-	-	-	-	-	-	-	-	-	-	-	-	-	-	-	-
Neutrophils cell/mm^3																
Grade 1	2 (6·5%)	1 (2·9%)	-	-	4 (12·9%)	1 (2·9%)	-	-	3 (10·3)	-	-	-	1(3·7%)	-	-	-
Grade 2	-	-	-	-	1 (3·2%)	-	-	-	2 (6·9%)	-	-	-	-	-	-	-
Grade 3	-	-	-	-	-	-	-	-	-	-	-	-	-	-	-	-
Eosinophil - cell/mm^3																
Grade 1	-	-	-	-	-	1 (2·9%)	-	-	-	1 (2·9%)	-	1 (3·2%)	-	1(2·9%)	-	-
Grade 2	-	-	-	-	-	-	-	-	-	-	-	-	-	-	-	-
Grade 3	-	-	-	-	-	-	-	-	-	-	-	-	-	-	-	-
Platelets cell/mm^3																
Grade 1	-	-	1 (2·9%)	-	1 (3·2)	1 (2·9)	-	2 (5·9%)	-	-	-	-	-	-	-	1(3·3%)
Grade 2	-	-	-	-	-	-	-	-	-	-	-	-	-	-	-	-
Grade 3	-	-	-	-	-	-	-	-	-	-	-	-	-	-	-	-
BUN mg/dL																
Grade 1	-	-	-	1 (2·9)	-	-	-	-	-	-	-	-	-	1(2·9%)	-	1(3·3%)
Grade 2	-	-	-	-	-	-	-	-	-	-	-	-	-	-	-	-
Grade 3	-	-	-	-	-	-	-	-	-	-	-	-	-	-	-	-
Creatinine – mg/dL																
Grade 1	-	-	-	-	-	1 (2·9)	-	-	-	-	1 (3·1%)	-	-	1(2·9%)	1(3·1%)	1(3·3%)

Grade 2	-	-	-	-	-	-	-	-	-	-	-	-	-	-	-	-
Grade 3	-	-	-	-	-	-	-	-	-	-	-	-	-	-	-	-
Alkaline phosphatase IU/L																
Grade 1	2 (6·4%)	-	1 (2·9%)	-	1(3·2%)	-	-	1 (2·9%)	-	-	-	1 (3·2%)	1(3·7%)	-	-	-
Grade 2	-	-	-	-	-	-	-	-	-	-	-	-	-	-	-	-
Grade 3	-	-	-	-	-	-	-	-	-	-	-	-	-	-	-	-
ALT IU/L																
Grade 1	4 (12·9%)	4 (11·6%)	2 (5·9%)	2 (5·9%)	3 (9·7%)	4 (11·7%)	3 (8·8%)	3 (8·8%)	1 (3·4%)	3 (8·8%)	1 (3·1%)	2 (6·4%)	2(7·4%)	3(8·8%)	4(1·2%)	2(6·6%)
Grade 2	-	-	-	-	-	-	-	2 (5·9%)	-	-	-	-	-	-	-	1(3·3%)
Grade 3	-	-	-	-	-	-	-	-	-	-	-	1 (3·2%)	-	-	-	-
AST IU/L																
Grade 1	1 (3·2%)		3 (8·8%)	1 (2·9%)	-	-	-	-	-	1 (2·9%)	-	2 (6·4%)	-	1(2·9%)	2(6·2%)	1(3·3%)
Grade 2	-	-	-	-	-	-	-	-	-	-	-	-	-	-	-	-
Grade 3	-	-	-	-	-	-	-	-	-	-	-	-	-	-	-	-
Bilirubin total																
Grade 1	-	-	-	-	1 (3·2%)	-	-	-	-	-	-	-	-		1(3·1%)	
Grade 2	-	1 (2·9%)	-	1 (2·9%)	1 (3·2%)	-	-	-	-	-	-	-	-	-	-	-
Grade 3	-	-	-	-	-	-	-	-	-	-	-	-	-	-	-	-
U/A, RBC			-													
Grade 1	1 (3·2%)	-	-	1 (2·9%)	1 (3·2%)	-	-	1 (2·9%)	-	1 (2·9%)	1 (3·1%)	-	-	-	-	3(10.0%)
Grade 2	-	-	1 (2·9%)	2 (5·6)	-	-	1 (2·9%)	1 (2·9%)	-	-	-	-	-	-	-	1(3·3%)
Grade 3	-	-	-	-	-	-	-	-	-	-	-	-	-	-	-	-

Table S15 Geometric means of IgG antibody responses (presented as area under the curve, AUC) against S antigen (Variant in Wuhan) in the intervention groups over the predefined study time schedule

GM_{AUC} (95% CI)	Baseline	Day 7	Day 14	Day 21	Day 28	Day 35	Day 65	Day 150
Placebo	19866 (17785-22191)	19004 (16850-21433)	22026 (18349-26438)	22833 (18780-27762)	24202 (19132-30615)	30051 (20336-44408)	26786 (14763-48600)	19710 (11639-33379)
Vac. 5 µ	20609 (18801-22591)	24400 (20170-29517)	33422 (24388-45803)	35035 (24508-50085)	48547 (30821-76468)	107840 (66618-174569)	95006 (43421-207879)	63058 (30307-131204)
Vac. 10 µ	19155 (17134-21414)	23230 (17752-30398)	30348 (21645-42550)	37780 (25664-55616)	51401 (33080-79870)	133944 (81582-219912)	98702 (49261-197766)	110449 (53356-228633)
Vac. 20 µ	18053 (16133-20202)	19364 (16666-22499)	23859 (18878-30153)	27667 (20413-37499)	52734 (35219-78961)	202801 (138281-297425)	137880 (66921-284078)	110515 (53899-226601)
GMR_{AUC} (95% CI)								
Placebo	1	1	1	1	1	1	1	1
Vac. 5 µ	1·28 (1·08-1·52)	1·36 (1·00-1·84)	1·50 (0·99-2·28)	1·50 (0·92-2·45)	1·96 (1·03-3·73)	3·44 (1·66-7·14)	3·55 (1·69-11·25)	3·20 (1·70-9·03)
Vac. 10 µ	1·01 (0·85-1·20)	1·21 (0·90-1·64)	1·29 (0·85-1·96)	1·57 (0·96-2·57)	1·97 (1·03-3·78)	4·19 (2·00-8·79)	3·68 (1·61-15·33)	5·60 (1·68-27·39)
Vac. 20 µ	0·90 (0·75-1·07)	0·93 (0·69-1·26)	1·03 (0·68-1·56)	1·15 (0·70-1·89)	1·96 (1·02-3·78)	6·12 (2·90-12·89)	5·15 (1·66-25·79)	5·61 (1·70-25·28)
$GMFI_{AUC}$ (95% CI)								
Placebo	1	1·0 (0·87-1·04)	1·1 (0·93-1·35)	1·2 (0·95-1·43)	1·2 (0·96-1·59)	1·5 (1·04-2·30)	1·4 (0·80-2·48)	1·0 (0·62-1·58)
Vac. 5 µ	1	1·2 (0·99-1·42)	1·6 (1·19-2·21)	1·7 (1·19-2·44)	2·4 (1·51-3·66)	5·2 (3·26-8·40)	5·2 (2·37-11·19)	3·2 (1·51-6·61)
Vac. 10 µ	1	1·2 (0·96-1·53)	1·6 (1·16-2·18)	2·0 (1·34-2·86)	2·6 (1·73-4·06)	6·9 (4·27-11·14)	5·6 (2·76-11·19)	5·3 (2·59-10·93)
Vac. 20 µ	1	1·1 (0·94-1·22)	1·3 (1·05-1·66)	1·6 (1·15-2·12)	3·0 (2·03-4·32)	11·4 (7·79-16·66)	8·7 (4·28-17·87)	6·1 (2·90-13·00)
$GMFR_{AUC}$ (95% CI)								
Placebo	1	1	1	1	1	1	1	1
Vac. 5 µ	1	1·24 (0·83-1·89)	1·45 (0·97-2·16)	1·51 (1·01-2·25)	1·92 (1·28-2·85)	3·45 (2·30-5·16)	1·00 (1·00-6·62)	6·36 (3·60-11·24)
Vac. 10 µ	1	1·27 (0·85-1·67)	1·42 (0·95-2·11)	1·70 (1·14-2·54)	2·15 (1·44-3·22)	4·54 (3·02-6·83)	3·69 (2·06-6·95)	2·99 (1·65-5·41)
Vac. 20 µ	1	1·12 (0·76-2·16)	1·18 (0·79-1·76)	1·33 (0·89-1·99)	2·37 (1·58-3·56)	7·06 (4·68-10·65)	4·01 (2·31-11·24)	5·24 (2·92-9·42)
Seroconversion[a] (n/N, %)								
Placebo	-	0/30 (0 %)	2/29 (7 %)	2/29 (7 %)	2/29 (7 %)	4/27 (15 %)	4/19 (21 %)	2/15 (13 %)
Vac. 5 µ	-	1/34 (3 %)	7/34 (21%)	6/34 (18 %)	10/34 (29 %)	19/34 (56 %)	9/19 (47 %)	10/23 (43 %)
Vac. 10 µ	-	4/34 (12 %)	5/34 (15 %)	7/33 (21 %)	11/32 (34 %)	21/32 (66 %)	16/28 (57 %)	15/25 (60 %)
Vac. 20 µ	-	1/34 (3 %)	3/34 (9 %)	5/32 (16 %)	10/31 (32 %)	24/30 (80 %)	14/22 (63 %)	14/22 (63 %)
Seroconversion[b] (n/N, %)								
Placebo	-	1/30 (3 %)	2/29 (7 %)	2/29 (7 %)	3/29 (10 %)	4/27 (15 %)	5/19 (26 %)	2/15 (13 %)
Vac. 5 µ	-	1/34 (3 %)	7/34 (20 %)	7/34 (21 %)	12/34 (35 %)	22/34 (65 %)	12·19 (63 %)	12/23 (52 %)
Vac. 10 µ	-	4/34 (12 %)	7/34 (21 %)	10/33 (30 %)	15/32 (47 %)	23/32 (72 %)	16/28 (57 %)	15/25 (60 %)
Vac. 20 µ	-	1/34 (3 %)	5/34 (15 %)	6/32(19 %)	16/31 (52 %)	27/30 (87 %)	16/22 (72 %)	14/22 (63 %)

[a] based on four-fold increase from total geometric mean value; [b] based on 3 standard deviation increase from total geometric mean value; GMR, Geometric mean ratio; GMFI, Geometric mean fold increase; GMFR, Geometric mean fold Ratio

Table S16 Geometric means of IgG antibody responses (presented as area under the curve, AUC) against S1 antigen (Variant in Wuhan) in the intervention groups over the predefined study time schedule

**GM_{AUC} (95% CI)**	**Baseline**	**Day 7**	**Day 14**	**Day 21**	**Day 28**	**Day 35**	**Day 65**	**Day 150**
Placebo	20880 (18692-23323)	20191 (17766-22946)	22611 (18860-27108)	23484 (19277-28608)	25305 (19913-32157)	31085 (21507-44928)	32472 (16811-62722)	18571 (10693-32254)
Vac. 5 µ	26759 (24624-29080)	27426 (23164-32471)	33948 (25731-44790)	35184 (25701-48165)	49617 (32250-76337)	106933 (66768-171259)	87665 (39894-192638)	56417 (28462-111827)
Vac. 10 µ	21084 (18918-23498)	24504 (19056-31509)	29223 (22091-38657)	36870 (26627-51053)	49864 (32790-75829)	130390 (80846-210296)	93316 (44256-196763)	104141 (51205-211804)
Vac. 20 µ	18728 (16791-20888)	18771 (16403-21481)	23192 (18506-29065)	27089 (20024-36646)	49566 (33198-74004)	190136 (129416-279345)	136473 (65897-282638)	91915 (43398-194675)
GMR_{AUC} (95% CI)								
Placebo	1	1	1	1	1	1	1	1
Vac. 5 µ	1·28 (1·08-1·52)	1·36 (1·0-1·84)	1·50 (0·99-2·28)	1·50 (0·92-2·45)	1·96 (1·03-3·73)	3·46 (1·67-7·18)	2·70 (0·73-9·97)	3·04 (0·87-10·59)
Vac. 10 µ	1·01 (1·08-1·20)	1·21 (1·14-1·64)	1·24 (0·82-1·88)	1·57 (0·96-2·57)	1·97 (1·03-3·78)	4·22 (2·01-8·84)	2·87 (0·87-9·52)	5·61 (1·64-19·17)
Vac. 20 µ	0·90 (0·75 -1·07)	0·93 (0·69-1·26)	1·03 (0·68-1·56)	1·15 (0·70-1·89)	1·96 (1·02-3·78)	6·15 (2·92-12·97)	4·20 (1·19-14·84)	4·95 (1·40-17·45)
$GMFI_{AUC}$ (95% CI)								
Placebo	1·0 (1·00-1·00)	1·0 (0·88-1·04)	1·1 (0·91-1·29)	1·1 (0·92-1·37)	1·2 (0·95-1·56)	1·5 (1·02-2·14)	1·7 (0·88-3·23)	0·9 (0·51-1·47)
Vac. 5 µ	1·0 (1·00-1·00)	1·0 (0·88-1·19)	1·3 (0·98-1·64)	1·3 (0·98-1·77)	1·9 (1·23-2·79)	4·0 (2·51-6·37)	3·6 (1·67-7·89)	2·1 (1·10-4·20)
Vac. 10 µ	1·0 (1·00-1·00)	1·2 (0·95-1·42)	1·4 (1·07-1·83)	1·7 (1·25-2·40)	2·3 (1·52-3·54)	6·1 (3·77-9·78)	4·7 (2·14-10·16)	4·6 (2·25-9·59)
Vac. 20 µ	1·0 (1·00-1·00)	1·0 (0·90-1·12)	1·2 (1·02-1·51)	1·5 (1·11-1·94)	2·7 (1·83-3·88)	10·2 (7·01-14·90)	8·1 (4·01-16·51)	4·9 (2·26-10·60)
$GMFR_{AUC}$ (95% CI)								
Placebo	1	1	1	1	1	1	1	1
Vac. 5 µ	1	1·07 (0·72-1·78)	1·17 (0·79-1·72)	1·19 (0·81-1·77)	1·52 (1·03-2·25)	2·73 (1·84-4·04)	2·30 (1·26-5·18)	2·41 (1·31-4·43)
Vac. 10 µ	1	1·21 (0·82-1·53)	1·28 (0·87-1·89)	1·55 (1·05-2·29)	1·92 (1·29-2·84)	4·16 (2·80-6·19)	2·93 (1·66-9·33)	5·21 (2·85-9·54)
Vac. 20 µ	1	1·04 (0·71-1·72)	1·14 (0·77-1·68)	1·29 (0·87-1·91)	2·17 (1·47-3·22)	6·60 (4·42-9·85)	5·18 (2·88-4·43)	5·40 (2·92-9·99)
Seroconversion[a] (n/N, %)								
Placebo	-	0/30 (0 %)	1/29 (3 %)	2/29 (7 %)	2/29 (7 %)	4/27 (15 %)	5/19 (26%)	2/15 (13 %)
Vac. 5 µ	-	1/34 (3 %)	7/34 (21 %)	6/34 (18 %)	8/34 (24 %)	16/34 (47 %	9/19 (47 %)	10/23 (43 %)
Vac. 10 µ	-	2/34 (6 %)	3/34 (9 %)	6/33 (18 %)	9/32 (28 %)	20/32 (63 %)	16/28 (57%)	15/25 (60 %)
Vac. 20 µ	-	0/34 (0 %)	2/34 (6 %)	4/32 (13 %)	9/31 (29%)	24/30 (77 %)	14/22 (63%)	13/22 (59 %)
Seroconversion[b] (n/N, %)								
Placebo	-	0/30 (0 %)	2/29 (7 %)	2/29 (7 %)	3/29 (10 %)	4/27 (15 %)	5/19 (26 %)	2/15(13 %)
Vac. 5 µ	-	1/34 (3%)	7/34 (21%)	6/34 (18%)	10/34 (29%)	20/34 (59%)	12/19 (63 %)	11/23 (48 %)
Vac. 10 µ	-	4/34 (12%)	6/34 (18%)	10/33 (30%)	12/32 (37%)	22/32 (69%)	16/28 (57 %)	15/25 (60 %)
Vac. 20 µ	-	1/34 (3%)	5/34 (14·7%)	6/32 (19%)	14/31 (45%)	27/30 (87%)	15/22 (68 %)	13/22 (59 %)

[a] based on four-fold increase from total geometric mean value; [b] based on 3 standard deviation increase from total geometric mean value; GMR, Geometric mean ratio; GMFI, Geometric mean fold increase; GMFR, Geometric mean fold Ratio

Table S17 Geometric means of IgG antibody responses (presented as area under the curve, AUC) against S2 antigen (Variant in Wuhan) in the intervention groups over the predefined study time schedule

GM_{AUC} (95% CI)	Baseline	Day 7	Day 14	Day 21	Day 28	Day 35	Day 65	Day 150
Placebo	16905 (14051-20340)	15419 (12966-18336)	17087 (13698-21314)	18091 (14393-22739)	20135 (15239-26604)	24520 (16575-36274)	31406 (17161-57472)	18410 (10602-31969)
Vac. 5 µ	24600 (21496-28153)	22909 (19275-27227)	28238 (21364-37324)	31255 (22597-43232)	43783 (27891-68729)	99135 (61276-160385)	84499 (38768-184175)	58450 (28892-118247)
Vac. 10 µ	18132 (14933-22017)	19373 (14724-25491)	24973 (18356-33975)	30260 (21390-42808)	43714 (27908-68472)	119837 (72192-198928)	92232 (46613-182496)	80979 (43644-150253)
Vac. 20 µ	14638 (12224-17529)	14561 (12243-17317)	17060 (13693-21256)	22533 (16183-31375)	43070 (28321-65500)	171252 (117300-250020)	127229 (62840-257594)	86089 (44899-165069)
GMR_{AUC} (95% CI)								
Placebo	1	1	1	1	1	1	1	1
Vac. 5 µ	1·46 (1·09-1·95)	1·49 (1·05-2·10)	1·65 (1·05-2·61)	1·73 (1·02-2·93)	2·17 (1·10-4·30)	4·04 (1·90-8·59)	2·69 (1·67-6·82)	3·17 (1·62-10·91)
Vac. 10 µ	1·07 (0·80-1·44)	1·26 (0·89-1·77)	1·39 (0·88-2·20)	1·67 (0·99-2·84)	2·17 (1·09-4·34)	4·89 (2·28-10·50)	2·94 (1·60-9·78)	4·40 (1·61-22·65)
Vac. 20 µ	0·87 (0·65-1·16)	0·95 (0·67-1·34)	1·07 (0·68-1·68)	1·25 (0·73-2·12)	2·14 (1·06-4·30)	6·98 (3·23-15·08)	4·05 (1·64-16·61)	4·68 (1·63-23·81)
$GMFI_{AUC}$ (95% CI)								
Placebo	1	0·9 (0·79-1·02)	1·0 (0·81-1·27)	1·1 (0·83-1·39)	1·2 (0·87-1·63)	1·4 (0·94-2·13)	1·7 (0·94-3·15)	1·0 (0·58-1·75)
Vac. 5 µ	1	0·9 (0·82-1·05)	1·1 (0·91-1·45)	1·3 (0·96-1·71)	1·8 (1·17-2·70)	4·0 (2·50-6·49)	4·6 (2·12-10·09)	3·2 (1·58-6·48)
Vac. 10 µ	1	1·1 (0·90-1·27)	1·4 (1·03-1·85)	1·6 (1·14-2·36)	2·3 (1·46-3·72)	6·4 (3·78-10·78)	5·1 (2·55-10·00)	4·4 (2·39-8·23)
Vac. 20 µ	1	1·0 (0·86-1·15)	1·2 (0·94-1·44)	1·6 (1·15-2·15)	3·0 (2·00-4·40)	11·8 (7·95-17·55)	7·0 (3·44-14·11)	4·7 (2·46-9·04)
$GMFR_{AUC}$ (95% CI)								
Placebo	1	1	1	1	1	1		
Vac. 5 µ	1	1·03 (0·68-1·79)	1·13 (0·74-1·71)	1·21 (0·80-1·85)	1·49 (0·98-2·26)	2·83 (1·86-4·32)	1·98 (1·09-5·08)	2·24 (1·22-4·12)
Vac. 10 µ	1	1·18 (0·78-1·67)	1·36 (0·89-2·06)	1·55 (1·02-2·35)	1·97 (1·29-3·00)	4·54 (2·97-6·96)	2·88 (1·63-9·52)	3·80 (2·08-6·95)
Vac. 20 µ	1	1·10 (0·73-1·71)	1·15 (0·76-1·74)	1·45 (0·95-2·21)	2·46 (1·61-3·76)	8·23 (5·36-12·63)	5·29 (2·94-4·12)	5·34 (2·89-9·87)
Seroconversion[a] (n/N, %)								
Placebo	-	0/30 (0 %)	2/29 (7 %)	2/29 (7 %)	2/29 (7 %)	3/27 (11 %)	4/19 (21 %)	2/15 (13 %)
Vac. 5 µ	-	1/34 (3 %)	6/34 (18 %)	6/34 (18 %)	10/34 (30 %)	16/34 (47 %)	9/19 (47 %)	10/23 (43 %)
Vac. 10 µ	-	2/34 (6 %)	4/34 (12 %)	7/33 (21 %)	10/32 (31 %)	21/32 (66 %)	16/28 (57 %)	15/25 (60 %)
Vac. 20 µ	-	0/34 (0 %)	2/34 (6 %)	4/32 (12 %)	10/31 (32 %)	25/30 (81 %)	14/22 (63 %)	14/22 (63 %)
Seroconversion[b] (n/N, %)								
Placebo	-	0/30 (0 %)	1/29 (3 %)	2/29 (7 %)	2/29 (7 %)	3/27 (11 %)	4/19 (21 %)	2/15 (13 %)
Vac. 5 µ	-	1/34 (3 %)	5/34 (15 %)	5/34 (15 %)	7/34 (26 %)	16/34 (47 %)	9/19 (47 %)	9/23 (39 %)
Vac. 10 µ	-	2/34 (6 %)	4/34 (12 %)	5/33 (15 %)	8/32 (25 %)	20/32 (62 %)	16/28 (57 %)	15/25 (60 %)
Vac. 20 µ	-	0/34 (0 %)	2/34 (6 %)	4/32 (4 %)	8/31 (26 %)	25/30 (80 %)	14/22 (63 %)	14/22 (63 %)

[a] based on four-fold increase from total geometric mean value; [b] based on 3 standard deviation increase from total geometric mean value; GMR, Geometric mean ratio; GMFI, Geometric mean fold increase; GMFR, Geometric mean fold Ratio

Table S18 Geometric means of IgG antibody responses (presented as area under the curve, AUC) against RBD antigen (Variant in Wuhan) in the intervention groups over the predefined study time schedule

GM_{AUC} (95% CI)	Baseline	Day 7	Day 14	Day 21	Day 28	Day 35	Day 65	Day 150
Placebo	20532 (17987-23437)	20268 (17459-23530)	23526 (19364-28582)	25030 (20442-30648)	26501 (20714-33905)	32464 (22271-47323)	31705 (16455-61091)	17909 (10490-30575)
Vac. 5 µ	28265 (25294-31584)	29537 (24776-35213)	37005 (27859-49154)	35757 (26807-47695)	53024 (35080-80147)	110359 (69567-175070)	85377 (39632-183925)	60552 (29271-125260)
Vac. 10 µ	21456 (18994-24237)	24581 (19238-31406)	31905 (23280-43725)	39309 (27567-56053)	49860 (32620-76212)	132037 (79918-218145)	96315 (49064-189071)	107030 (52748-217171)
Vac. 20 µ	18705 (16844-20771)	20483 (17792-23582)	24783 (19505-31489)	28275 (21044-37992)	53271 (35951-78937)	190289 (131893-274538)	133917 (66626-269168)	103054 (50656-209652)
GMR_{AUC} (95% CI)								
Placebo	1	1	1	1	1	1	1	1
Vac. 5 µ	1·38 (1·13-1·68)	1·45 (1·06-1·98)	1·57 (1·00-2·47)	1·43 (0·87-2·34)	1·99 (1·06-3·76)	3·40 (1·63-7·07)	2·69 (1·68-6·82)	3·38 (1·68-10·38)
Vac. 10 µ	1·05 (0·86-1·27)	1·21 (0·89-1·64)	1·30 (0·83-2·05)	1·57 (0·96-2·57)	1·88 (0·99-3·58)	4·07 (1·94-8·55)	3·04 (1·60-10·49)	5·98 (1·67-32·79)
Vac. 20 µ	0·91 (0·75-1·11)	1·01 (0·74-1·37)	1·05 (0·67-1·65)	1·13 (0·69-1·86)	2·01 (1·05-3·84)	5·88 (2·78-12·44)	4·22 (1·65-17·99)	5·75 (1·69-27·94)
$GMFI_{AUC}$ (95% CI)								
Placebo	1	1·0 (0·89-1·07)	1·1 (0·95-1·36)	1·2 (0·99-1·47)	1·3 (0·99-1·65)	1·6 (1·07-2·25)	1·4 (0·75-2·78)	0·8 (0·48-1·39)
Vac. 5 µ	1	1·0 (0·91-1·21)	1·3 (1·01-1·69)	1·3 (0·99-1·64)	1·9 (1·27-2·76)	3·9 (2·46-6·19)	3·9 (1·80-8·36)	2·8 (1·33-5·69)
Vac. 10 µ	1	1·1 (0·95-1·39)	1·5 (1·11-1·98)	1·8 (1·29-2·56)	2·3 (1·50-3·46)	6·0 (3·68-9·91)	4·4 (2·23-8·59)	4·9 (2·40-9·87)
Vac. 20 µ	1	1·1 (0·97-1·24)	1·3 (1·06-1·66)	1·5 (1·15-2·03)	2·9 (1·95-4·21)	10·2 (7·01-14·91)	6·1 (3·03-12·23)	4·7 (2·30-9·53)
$GMFR_{AUC}$ (95% CI)								
Placebo	1	1	1	1	1	1	1	1
Vac. 5 µ	1	1·06 (0·72-1·71)	1·15 (0·78-1·69)	1·07 (0·73-1·58)	1·46 (0·99-2·15)	2·53 (1·71-3·75)	2·20 (1·23-5·16)	2·53 (1·40-4·57)
Vac. 10 µ	1	1·17 (0·79-1·64)	1·30 (0·89-1·92)	1·51 (1·02-2·22)	1·78 (1·21-2·63)	3·93 (2·65-5·83)	2·98 (1·72-8·86)	5·35 (2·98-9·60)
Vac. 20 µ	1	1·11 (0·76-1·69)	1·16 (0·79-1·71)	1·25 (0·85-1·85)	2·21 (1·49-3·26)	6·57 (4·42-9·77)	5·02 (2·84-4·57)	6·22 (3·43-11·28)
Seroconversion[a] (n/N, %)								
Placebo	-	0/30 (0 %)	1/29 (3 %)	2/29 (7 %)	2/29 (7 %)	4/27 (15 %)	4/19 (21%)	2/15 (13%)
Vac. 5 µ	-	1/34 (3 %)	7/34 (21 %)	5/34 (15 %)	8/34 (23 %)	19/34 (56 %)	9/19 (47 %)	9/23 (39%)
Vac. 10 µ	-	3/34 (9 %)	5/34 (15 %)	6/33 (19 %)	8/32 (25 %)	20/32 (62 %)	16/28 (57 %)	15/25 (60%)
Vac. 20 µ	-	0/34 (0 %)	3/34 (9 %)	4/32 (12 %)	9/31 (29 %)	25/30 (81 %)	14/22 (63%)	14/22 (63%)
Seroconversion[b] (n/N, %)								
Placebo	-	0/30 (0 %)	2/29 (7 %)	2/29 (7 %)	3/29 (10 %)	4/27 (15 %)	5/19 (26%)	2/15 (13)
Vac. 5 µ	-	1/34 (3 %)	7/34 (21 %)	5/34 (15 %)	10/34 (29 %)	20/34 (59 %)	11/19 (58 %)	11/23 (47%)
Vac. 10 µ	-	4/34 (12 %)	5/34 (15 %)	9/33 (27 %)	12/32 (37 %)	21/32 (66 %)	16/28 (57 %)	15/25 (60%)
Vac. 20 µ	-	0/34 (3 %)	5/34 (15 %)	6/32 (19 %)	12/31 (39 %)	25/30 (81 %)	15/22 (68%)	14/22 (63%)

[a] based on four-fold increase from total geometric mean value; [b] based on 3 standard deviation increase from total geometric mean value; GMR, Geometric mean ratio; GMFI, Geometric mean fold increase; GMFR, Geometric mean fold Ratio

Table S19 Geometric means of IgG antibody responses (presented as area under the curve, AUC) against NTD antigen (Variant in Wuhan) in the intervention groups over the predefined study time schedule

GM_{AUC} (95% CI)	Baseline	Day 7	Day 14	Day 21	Day 28	Day 35	Day 65	Day 150
Placebo	17817 (15892-19975)	17806 (15692-20206)	20196 (16780-24306)	21268 (17354-26066)	23687 (18496-30335)	29117 (20005-42378)	33302 (17409-63705)	17935 (10501-30629)
Vac. 5 µ	23415 (21398-25623)	24821 (20997-29341)	31200 (23905-40722)	32219 (23893-43446)	45457 (29622-69758)	94237 (58359-152174)	76585 (31446-186519)	61779 (29604-128925)
Vac. 10 µ	18907 (16517-21643)	21386 (16651-27467)	27546 (20662-36725)	33150 (23466-46830)	46803 (30227-72469)	122813 (74433-202637)	100344 (51063-197184)	103198 (50877-209324)
Vac. 20 µ	16517 (14657-18612)	17824 (15303-20760)	22569 (17685-28802)	26628 (19590-36195)	49998 (33833-73886)	173234 (107926-278062)	137192 (67774-277715)	98998 (49029-199893)
GMR_{AUC} (95% CI)								
Placebo	1	1	1	1	1	1	1	1
Vac. 5 µ	1·31 (1·08-1·60)	1·40 (1·03-1·91)	1·54 (1·01-2·37)	1·50 (0·91-2·47)	1·93 (1·01-3·67)	3·24 (1·51-6·93)	2·30 (1·71-4·71)	3·44 (1·68-10·70)
Vac. 10 µ	1·06 (0·87-1·29)	1·21 (0·89-1·64)	1·31 (0·85-2·00)	1·62 (0·98-2·66)	1·98 (1·03-3·81)	4·23 (1·95-9·15)	3·01 (1·63-9·49)	5·75 (1·67-30·27)
Vac. 20 µ	0·93 (0·76-1·13)	1·01 (0·74-1·37)	1·12 (0·73-1·71)	1·24 (0·75-2·05)	2·11 (1·09-4·09)	6·67 (3·07-14·52)	4·12 (1·68-15·49)	5·52 (1·69-25·79)
$GMFI_{AUC}$ (95% CI)								
Placebo	1	1·0 (0·92-1·07)	1·1 (0·95-1·35)	1·2 (0·97-1·46)	1·3 (1·02-1·72)	1·6 (1·11-2·35)	2·0 (1·07-3·83)	1·0 (0·58-1·61)
Vac. 5 µ	1	1·1 (0·92-1·22)	1·3 (1·03-1·72)	1·4 (1·04-1·83)	1·9 (1·29-2·93)	4·0 (2·52-6·42)	3·5 (1·47-8·42)	2·7 (1·29-5·69)
Vac. 10 µ	1	1·1 (0·93-1·37)	1·5 (1·11-1·91)	1·7 (1·23-2·44)	2·4 (1·56-3·75)	6·4 (3·87-10·46)	5·8 (2·85-11·82)	5·0 (2·42-10·39)
Vac. 20 µ	1	1·1 (0·96-1·21)	1·4 (1·10-1·70)	1·6 (1·22-2·19)	3·1 (2·10-4·44)	10·6 (6·62-16·92)	9·4 (4·66-18·81)	6·1 (2·86-12·81)
$GMFR_{AUC}$ (95% CI)								
Placebo	1	1	1	1	1	1	1	1
Vac. 5 µ	1	1·07 (0·71-1·71)	1·18 (0·78-1·77)	1·19 (0·79-1·79)	1·46 (0·97-2·20)	2·52 (1·67-3·80)	1·83 (1·00-5·25)	2·57 (1·39-4·76)
Vac. 10 µ	1	1·14 (0·76-1·63)	1·29 (0·86-1·94)	1·47 (0·98-2·22)	1·84 (1·22-2·77)	4·00 (2·64-6·07)	2·97 (1·68-8·86)	5·08 (2·77-9·32)
Vac. 20 µ	1	1·09 (0·72-1·77)	1·21 (0·80-1·82)	1·36 (0·90-2·06)	2·28 (1·51-3·44)	6·55 (4·31-9·95)	4·91 (2·72-4·76)	5·90 (3·18-10·94)
Seroconversion[a] (n/N, %)								
Placebo	-	0/30 (0 %)	2/29 (7 %)	2/29 (7 %)	2/29 (7 %)	4/27 (15 %)	5/19 (26%)	2/15 (13%)
Vac. 5 µ	-	1/34 (3 %)	6/34 (18 %)	6/34 (18 %)	9/34 (26 %)	17/34 (50 %)	9/19 (47 %)	10/23 (43%)
Vac. 10 µ	-	2/34 (6 %)	4/34 (12 %)	7/33 (21 %)	11/32 (34 %)	21/32 (65 %)	16/28 (57%)	15/25 (60%)
Vac. 20 µ	-	0/34 (0 %)	4/34 (12 %)	5/32 (15 %)	10/31 (32 %)	24/30 (77 %)	14/22 (63%)	14/22 (63 %)
Seroconversion[b] (n/N, %)								
Placebo	-	0/30 (0 %)	2/29 (7 %)	2/29 (7 %)	3/29 (10 %)	4/27 (15 %)	5/19 (26 %)	2/15 (13%)
Vac. 5 µ	-	1/34 (3 %)	7/34 (20 %)	6/34 (18 %)	10/34 (29 %)	19/34 (56 %)	12/19 (63 %)	10/23 (43 %)
Vac. 10 µ	-	4/34 (12 %)	7/34 (20 %)	9/33 (27 %)	13/32 (40 %)	23/32 (72 %)	16/28 (57%)	15/25 (60%)
Vac. 20 µ	-	1/34 (3 %)	5/34 (15 %)	6/32 (19 %)	15/31 (48 %)	25/30 (81 %)	15/22 (68%)	14/22 (63%)

[a] based on four-fold increase from total geometric mean value; [b] based on 3 standard deviation increase from total geometric mean value; GMR, Geometric mean ratio; GMFI, Geometric mean fold increase; GMFR, Geometric mean fold Ratio

Table S20 Geometric means of IgG antibody responses (presented as area under the curve, AUC) against N antigen (Variant in Wuhan) in the intervention groups over the predefined study time schedule

GM_{AUC} (95% CI)	Baseline	Day 7	Day 14	Day 21	Day 28	Day 35
Placebo, n=27	17625 (15611-19900)	15486 (14154-16942)	14806 (13283-16504)	13641 (12662-14695)	14131 (13244-15078)	14342 (13314-15448)
Vac. 5 μ, n=34	23755 (21993-25659)	18277 (17233-19383)	17141 (15948-18424)	15263 (14222-16381)	14838 (14020-15703)	15493 (14307-16778)
Vac. 10 μ, n=32	18685 (17065-20459)	15786 (14278-17454)	14166 (13098-15321)	14613 (13670-15621)	15101 (14087-16187)	15610 (14321-17015)
Vac. 20 μ, n=31	16544 (14735-18576)	14479 (13179-15909)	13214 (12184-14332)	13752 (12698-14894)	16016 (13257-19350)	17191 (15541-19016)
GMR_{AUC} (95% CI)						
Placebo, n=27	1	1	1	1	1	1
Vac. 5 μ, n=34	1·35 (1·07-1·60)	1·18 (1·06-1·37)	1·16 (1·06-1·34)	1·12 (1·05-1·26)	1·05 (1·08-1·26)	1·08 (1·06-1·25)
Vac. 10 μ, n=32	1·06 (1·07-1·26)	1·02 (1·06-1·18)	0·96 (1·06-1·10)	1·07 (1·05-1·21)	1·07 (1·08-1·28)	1·09 (1·06-1·26)
Vac. 20 μ, n=31	0·94 (1·07-1·11)	0·94 (1·06-1·08)	0·89 (1·06-1·03)	1·01 (1·05-1·14)	1·13 (1·08-1·36)	1·20 (1·06-1·39)
$GMFI_{AUC}$ (95% CI)						
Placebo, n=27	1	0·9 (0·80-0·94)	0·8 (0·75-0·92)	0·8 (0·68-0·86)	0·8 (0·69-0·92)	0·8 (0·67-0·94)
Vac. 5 μ, n=34	1	0·8 (0·72-0·82)	0·7 (0·68-0·77)	0·6 (0·60-0·69)	0·6 (0·58-0·67)	0·7 (0·57-0·74)
Vac. 10 μ, n=32	1	0·8 (0·79-0·90)	0·8 (0·72-0·80)	0·8 (0·72-0·84)	0·8 (0·71-0·90)	0·8 (0·72-0·95)
Vac. 20 μ, n=31	1	0·9 (0·80-0·96)	0·8 (0·74-0·86)	0·8 (0·75-0·94)	1·0 (0·77-1·23)	1·0 (0·88-1·24)
$GMFR_{AUC}$ (95% CI)						
Placebo, n=27	1	1	1	1	1	1
Vac. 5 μ, n=34	1	0·88 (0·75-1·13)	0·86 (0·73-1·01)	0·83 (0·71-0·97)	0·78 (0·66-0·92)	0·81 (0·69-0·95)
Vac. 10 μ, n=32	1	0·96 (0·82-1·17)	0·90 (0·77-1·06)	1·01 (0·86-1·18)	1·00 (0·85-1·18)	1·03 (0·87-1·21)
Vac. 20 μ, n=31	1	1·00 (0·85-1·01)	0·95 (0·81-1·12)	1·08 (0·92-1·27)	1·21 (1·03-1·42)	1·29 (1·09-1·51)

GMR, Geometric mean ratio; GMFI, Geometric mean fold increase; GMFR, Geometric mean fold Ratio

Table S21 Geometric mean titer for neutralizing antibody titer the over the predefined study time schedule

	Baseline	Day 35	Day 65	Day 150
**GMT (95% CI)**				
**Placebo**	1.0 (1.00-1.00)	1.8 (0.99-3.30)	4.6 (0.52-40.69)	2.6 (0.00-618790.10)
**Vac. 5 µ**	1.1 (0.97-1.22)	10.5 (4.69-23.60)	4.4 (0.23-81.36)	2.2 (0.83-5.89)
**Vac. 10 µ**	1.0 (1.00-1.00)	20.1 (8.16-49.62)	143.1 (28.26-725.15)	17.4 (1.16-260.55)
**Vac. 20 µ**	1.0 (1.00-1.00)	37.4 (15.36-91.33)	80.3 (0.12-53885.13)	4.4 (0.75-26.42)
**GMR (95% CI)**				
**Placebo**	1	1	1	1
**Vac. 5 µ**	1.08 (0.98-1.20)	5.82 (1.46-23.13)	0.95 (0.03-27.05)	0.84 (0.02-28.22)
**Vac. 10 µ**	1.00 (0.91-1.10)	11.12 (2.74-45.09)	31.11 (1.46-663.67)	6.58 (0.17-259.11)
**Vac. 20 µ**	1.00 (0.91-1.10)	20.70 (5.05-84.76)	17.46 (0.47-652.38)	1.68 (0.03-82.75)
**Seroconversion[a] n(%)**				
**Placebo**	-	4/27 (15 %)	1/4 (25 %)	0/2 (0 %)
**Vac. 5 µ**	-	19/34 (56 %)	2/4 (50 %)	2/9 (22 %)
**Vac. 10 µ**	-	21/32 (66 %)	6/6 (100%)	3/6 (50 %)
**Vac. 20 µ**	-	24/31 (77 %)	2/3 (67 %)	1/4 (25 %)

[a] based on four-fold increase from total geometric mean value; GMT, Geometric mean titer; GMR, Geometric mean ratio

Figure S1 Geometric means of IgG antibody responses (presented as area under the curve, AUC) against S antigen in the intervention groups over the predefined study time schedule

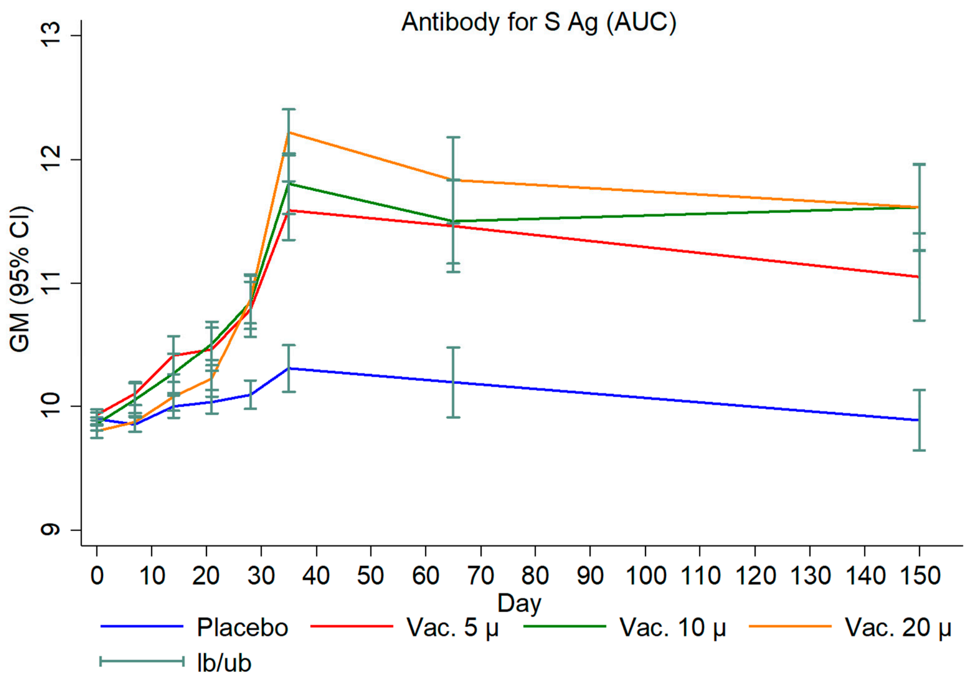

Figure S2 Geometric means of IgG antibody responses (presented as area under the curve, AUC) against S1 antigen in the intervention groups over the predefined study time schedule

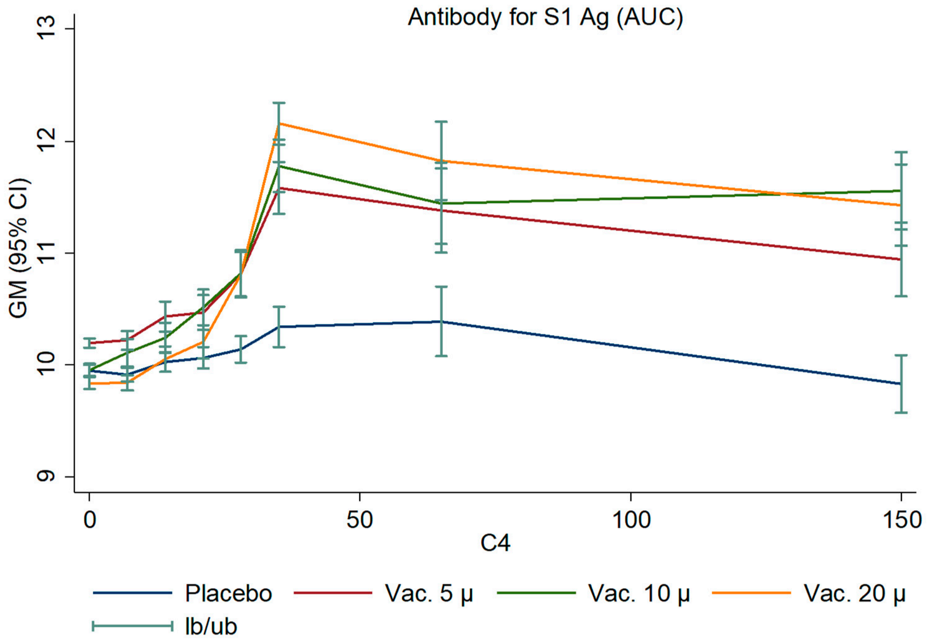

Figure S3 Geometric means of IgG antibody responses (presented as area under the curve, AUC) against S2 antigen in the intervention groups over the predefined study time schedule

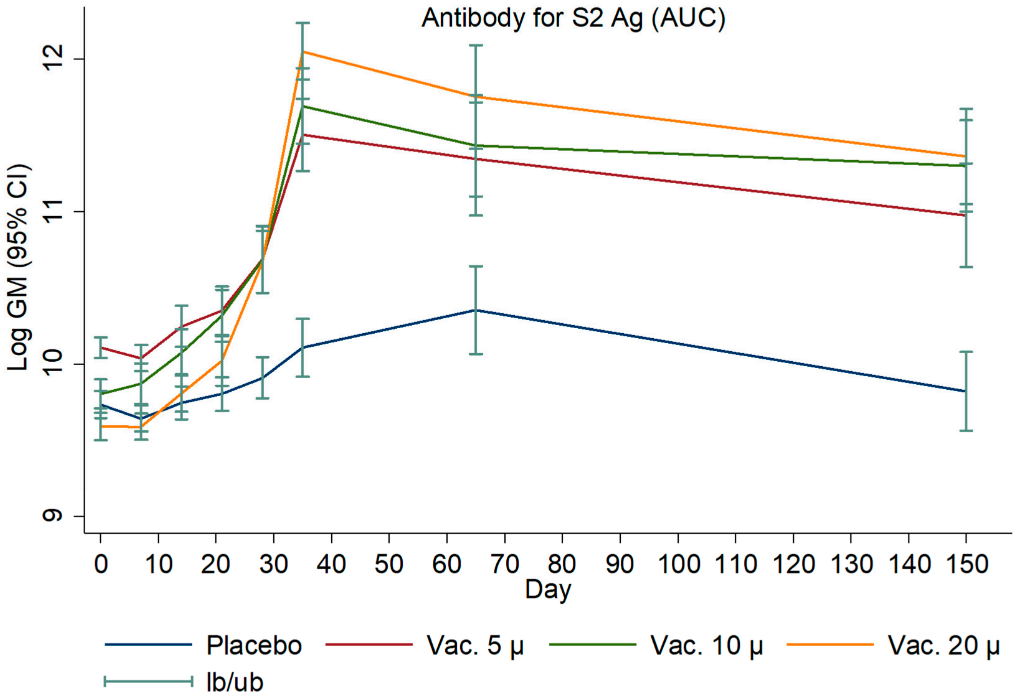

Figure S4 Geometric means of IgG antibody responses (presented as area under the curve, AUC) against RBD antigen in the intervention groups over the predefined study time schedule

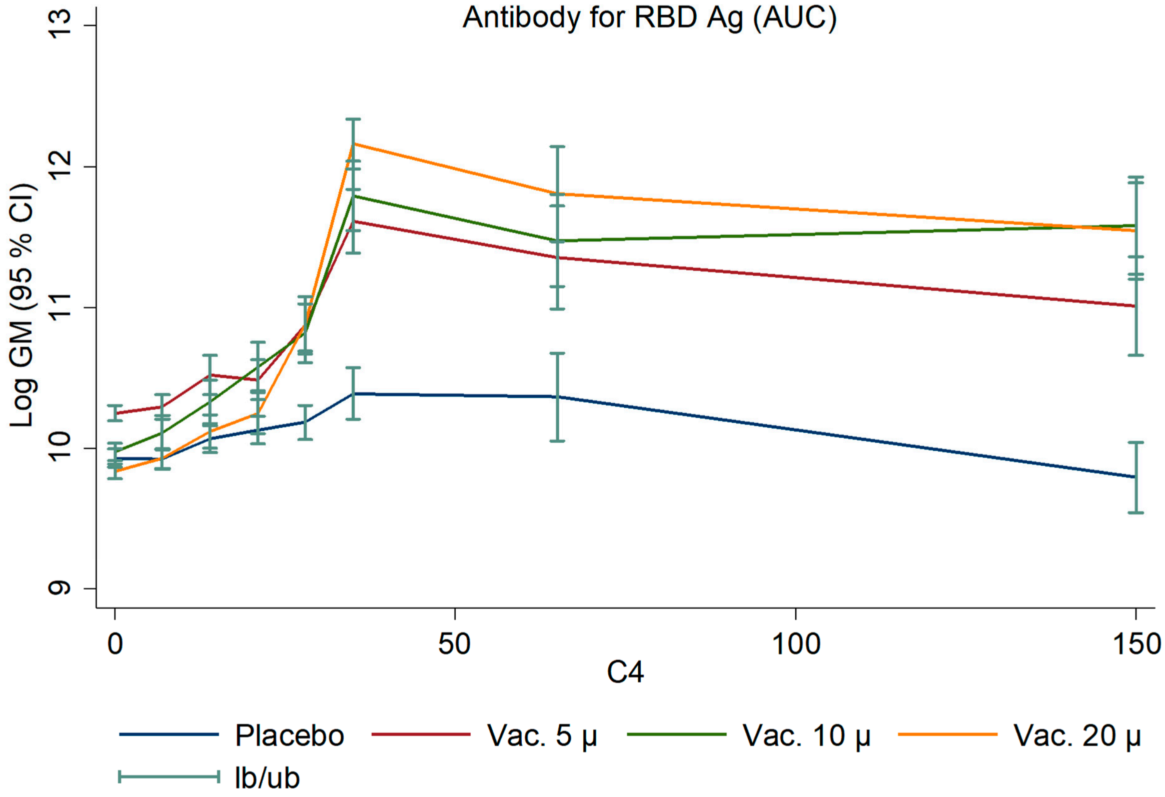

Figure S5 Geometric means of IgG antibody responses (presented as area under the curve, AUC) against NTD antigen in the intervention groups over the predefined study time schedule

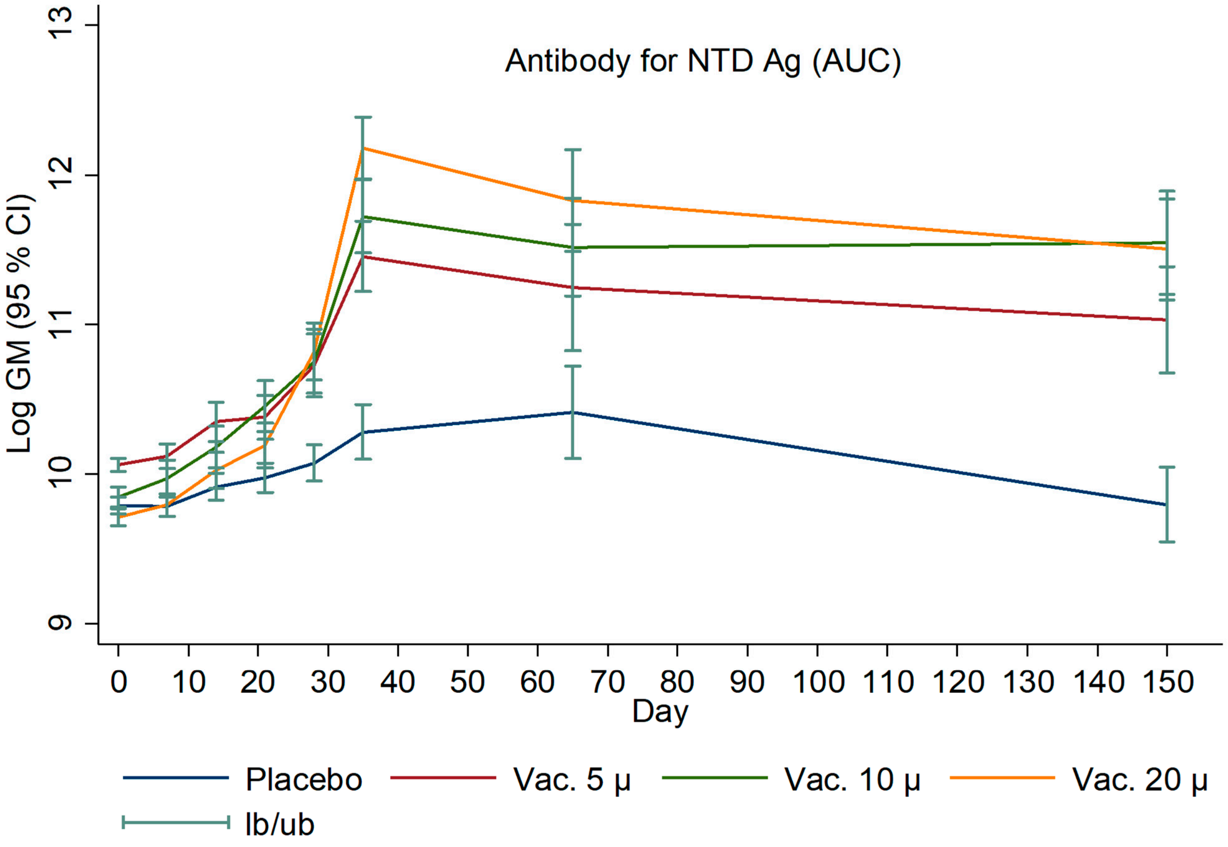

Figure S6 Scatter plots illustrating the correlation between neutralizing antibody responses and specific IgG ELISA antibody responses (AUC) at 2 weeks after the second dose (day 35) in the intervention groups. Nonparametric Spearman correlation estimates have been shown on the diagrams.

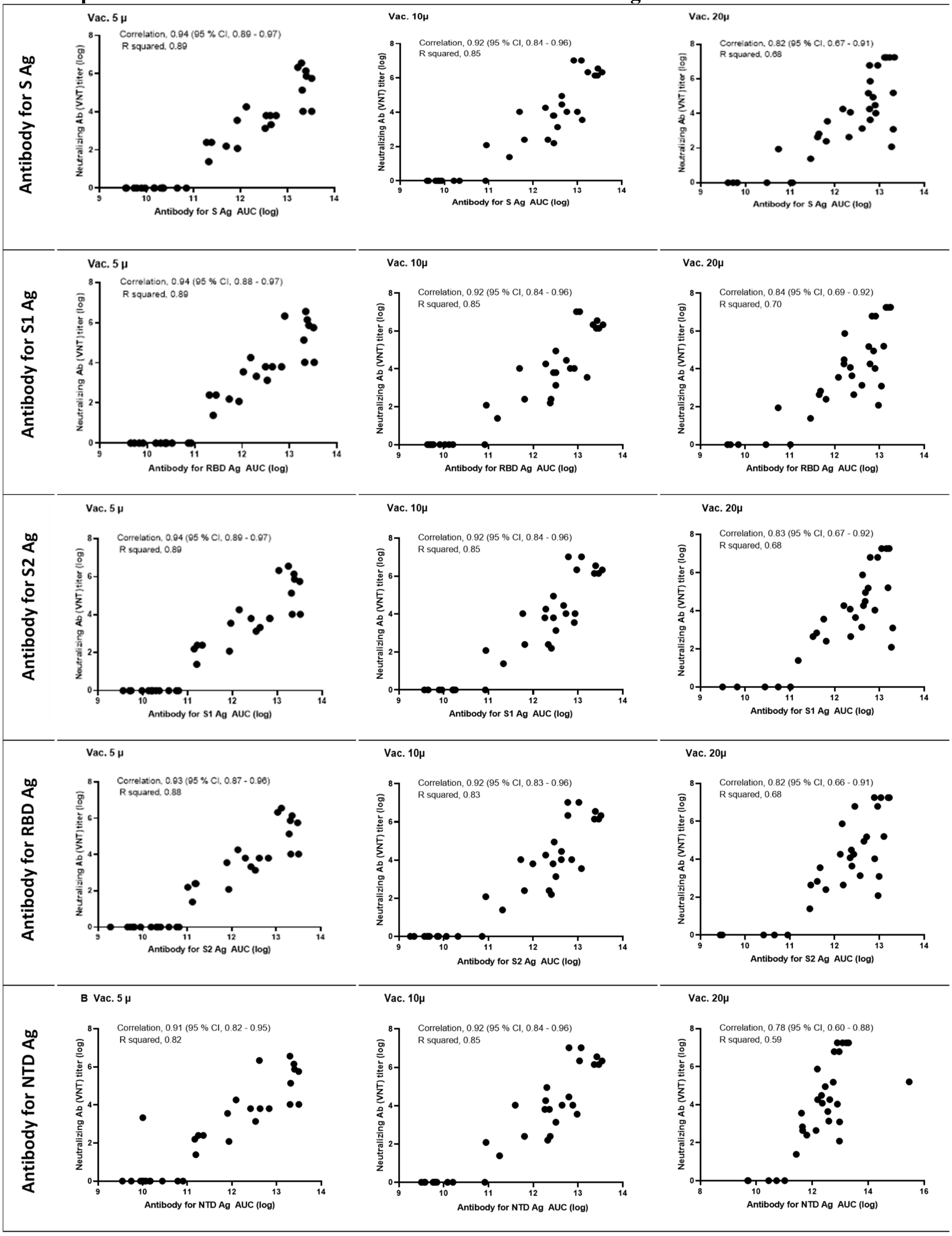

Figure S7 Scatter diagram of changes in percentage of CD3,CD4 and CD3,CD8 (cytotoxic) T cells in response to stimulation by S antigen measured by flow cytometry in peripheral blood mononuclear cell (PBMC) extract in the intervention groups at the day 35 compared to the baseline. Percentage means have been shown on the diagram.

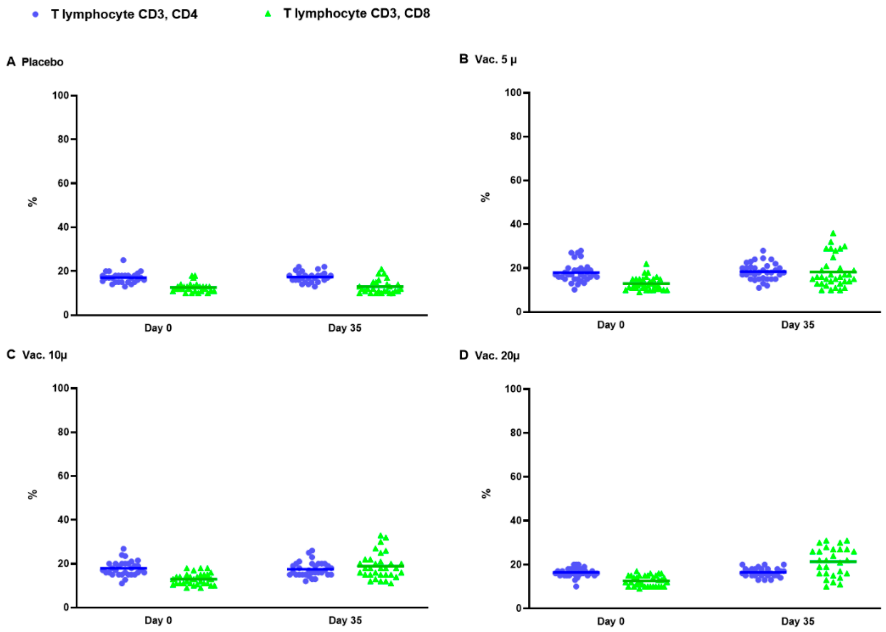

References

1. He Y, Zhou Y, Liu S, Kou Z, Li W, Farzan M, et al. Receptor-binding domain of SARS-CoV spike protein induces highly potent neutralizing antibodies: implication for developing subunit vaccine. Biochemical and biophysical research communications. 2004;324(2):773-81.
2. Du T, Yang C-L, Ge M-R, Liu Y, Zhang P, Li H, et al. M1 macrophage derived exosomes aggravate experimental autoimmune neuritis via modulating Th1 response. Frontiers in immunology. 2020:1603.
3. Miao Q, Zhang X-X, Han Q-X, Ren S-S, Sui R-X, Yu J-W, et al. The therapeutic potential of bilobalide on experimental autoimmune encephalomyelitis (EAE) mice. Metabolic Brain Disease. 2020;35(5):793-807.
